# One-Stage Pathway from Hollongdione to C17-Alkyne and Vinyl Chloride Following Mannich Bases and Carboxylic Acid

**DOI:** 10.3390/ijms25158356

**Published:** 2024-07-30

**Authors:** Zarema Galimova, Irina Smirnova, Alexander Lobov, Dmitriy Polovyanenko, Tatyana Rybalova, Oxana Kazakova

**Affiliations:** 1 Ufa Institute of Chemistry of the Ufa Federal Research Centre of the Russian Academy of Sciences, 71, pr. Oktyabrya, 450054 Ufa, Russia; chemizara@gmail.com (Z.G.); lobovan@anrb.ru (A.L.); obf@anrb.ru (O.K.); 2 N. N. Vorozhtsov Novosibirsk Institute of Organic Chemistry SB RAS, 630090 Novosibirsk, Russia; dpolo@nioch.nsc.ru (D.P.); rybalova@nioch.nsc.ru (T.R.)

**Keywords:** dammarane triterpenoids, hollongdione, alkyne, vinyl chloride, Mannich base, hollongdionoic acid

## Abstract

Hollongdione is the first recorded example of the occurrence of a dammarane hexanor-triterpene in nature possessing antiviral and cytotoxic activity. Its simple one-stage transformation into compounds with terminal alkyne and vinyl chloride fragments via the interaction with phosphorus halides is reported. The copper(I)-catalyzed Mannich reaction of 3-oxo-22,23,24,25,26,27-hexanor-dammar-20(21)-in **3** led to a series of aminomethylated products, while 17-carboxylic acid was obtained by ozone oxidation of 3-oxo-22,23,24,25,26,27-hexanor-dammar-20-chloro-20(21)-en **4**; the following direct amidation of the latter has been developed. The structures of all new molecules were established by spectroscopic studies that included 2D NMR correlation methods; the molecular structures of compounds **2**–**5** were determined by X-ray analysis.

## 1. Introduction

The unique and complex structures of natural products make them important sources for exploring novel areas of the chemical space [1,2]. The introduction of highly effective reaction centers in such molecules allows expanding the library of semi-synthetic compounds, with the aim of their future application as units in biochemical science. 

**Figure 1:**
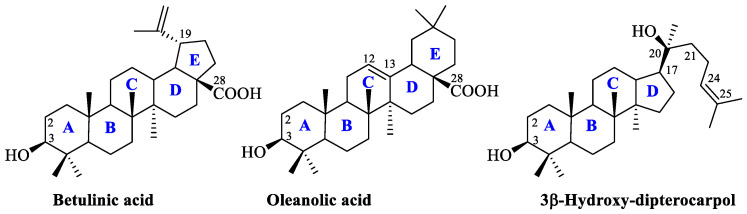
The general structures of lupane, oleanane, and dammarane type triterpenoids. The cycles in the structures of triterpenoids are marked in blue.

Pentacyclic triterpenoids comprise a diverse group that is widely present in plants and has stable skeleton and reactive sites suitable for structural transformations. Triterpenoids usually possess a secondary hydroxyl group or a carbon atom at position *C*3, and a primary hydroxyl group or carboxyl group at position *C*28 (the general structures of triterpenoids with the key sites for modification are presented in Figure 1). The most frequent modifications of the listed fragments include an acylation reaction resulting in various esters [3,4,5] and glycosides [6,7], since the addition of hydrosoluble moieties such as sugar substituents at the *C*3 and/or *C*28 positions can enhance both the water solubility and the pharmacological activity [8,9].

On the other hand, there is an oxygen function bonded to the ring *E* or *D*, a number of transformations of which have also been proposed in the literature. The most unique examples are Baeyer–Villiger oxidation of 20-oxo-30-nortaraxasteryl acetate that lead to *ε*-lactone [10]; 30-oxobetulinic acid conversion into a set of substituted dienes by the Wittig reaction with the use of triphenylphosphonium salts [11]; and an epimerization at *C*19 and a condensation reaction of several platanic carboxamides with the synthesis of novel *E*-ring *δ*−lactams [12]. Another attractive method of carbonyl group modification is a reaction of dehydration, which is a route to terminal acetylenes, which are key precursors in drug discovery [2,13]. One widely used method of accessing alkynes is an interaction of methyl ketones with phosphorous-based reagents. This approach was found to be a short-stage, simple experimental procedure with no by-product formation in the synthesis of terminal lupane-type alkynes [14,15] and was more efficient compared to other methods, such as conventional Pd-catalyzed Sonogashira coupling [16,17], the Grignard reaction [18] and propargylation of the *NH*-group [19].

**Figure 2:**
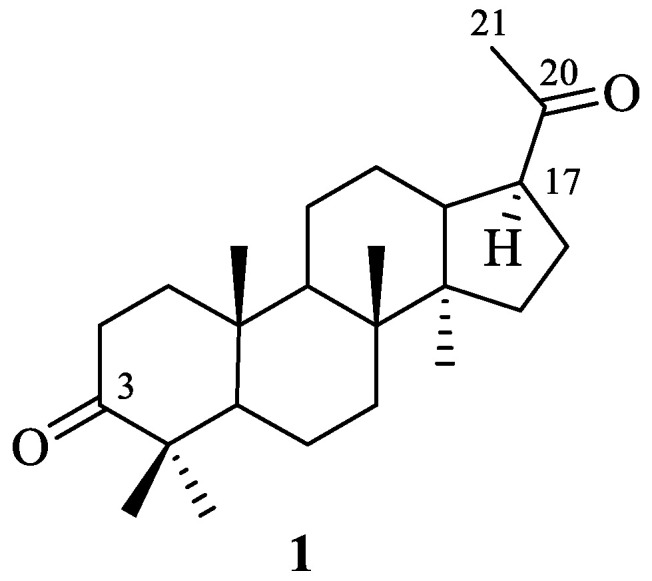
The structure of hollongdione **1**.

In this work, we decided to use dammarane-type triterpenoid hollongdione (22,23,24,25,26,27-hexanordammar-3,20-dione) **1** with a *C*17 acetyl group as an initial scaffold [20]. Hollongdione (the structure of which is presented in Figure 2) can be obtained by one-reactor synthesis from dipterocarpol, the main metabolite of *Dipterocarpus alatus* oleoresin or isolated from *Dipterocarpus pilosus*, *Gardenia aubryi*, *Chisocheton penduliflorus*, and leaves of *Euphorbia leucocephala*, which exhibits potent anti-influenza A virus activity [21,22,23]. Moreover, it is a crucial intermediate in the synthesis of the potent immunosuppressive agent 17α-23-(E)-dammara-20,23-diene-3β,25-diol and moderately active against small-cell lung cancer cells (NCI-H187) [24,25]. Arylidene derivatives of hollongdione, described before, induce antiproliferative activity against melanoma and breast cancer through pro-apoptotic and antiangiogenic mechanisms [26]. It is recognized as a hybrid molecule that contains cycle *A,* similar to triterpene, and a *C*17-acetyl group with a *β*-configuration attached to the *D* cycle, similar to the pregnane steroids. 

The choice of the initial structure can be justified by the fact that there are no attempts of acetylene fragment formation as well as its further modification in the family of dammarane triterpenoids or pregnane steroids, especially among the closest analogues like panaxadiol and protopanaxadiol. Just in a few cases, changes were made based on the protopanaxadiol ketones; different kinds of functionalities including hydroxy, fluoride, aldehyde, carboxy, ester, and enol ether were introduced for diversity of ring *D* [27,28]. 

Taking into account the above, we report herein a one-stage method of hollongdione modification into compounds with terminal alkyne and vinyl chloride fragments via the interaction with phosphorus halides. The obtained unsaturated hollongdiones were transformed to aminomethylated products and carboxamide. 

## 2. Results

Procedure of compound **2**

A solution of 3*β*-hydroxy-dipterocarpol (0.440 g; 1.0 mmol) in AcOH (30 mL) was refluxed for 2 h; the reaction mixture was cooled to 0 °C, and ozone was passed through it until the starting compound disappeared (TLC control). The mixture was then poured into H_2_O (40 mL), removed by filtration, and washed with H_2_O. The crude product was crystallized from benzene. 

3*β*-Acetoxy-22,23,24,25,26,27-hexanor-dammar-20-one (**2**)

White solid, yield: 96%, mp: 126–127 °C, [α]^20^_D_ = −60 (c 0.05, CHCl_3_). ^13^C NMR (CDCl_3_, δ ppm): 15.59 (C18); 15.88 (C30); 16.31 (C19); 16.50 (C29); 18.15 (C6); 21.26 (C11); 21.31 (C2′); 23.71 (C2); 25.59 (C12); 25.98 (C16); 27.98 (C28); 30.06 (C21); 31.57 (C15); 35.49 (C7); 37.14 (C10); 37.92 (C4); 38.81 (C1); 40.53 (C8); 45.14 (C13); 50.07 (C14); 50.65 (C9); 54.27 (C17); 55.98 (C5); 80.87 (C3); 171.00 (C1′); 212.32 (C20). ^1^H NMR (CDCl_3_, δ ppm, *J* Hz): 0.83 (dd, 1H, ^3^*J*_5-6ax_ = 11.7, ^3^*J*_5-6eq_ = 2.6, H-5); 0.85 (s, 3H, H-29); 0.86 (s, 3H, H-28); 0.87 (s, 3H, H-30); 0.88 (s, 3H, H-19); 0.98 (s, 3H, H-18); 1.05 (td, 1H, ^2^*J* = 12.2, ^3^*J*_1ax-2ax_ = 12.2, ^3^*J*_1ax-2ax_ = 5.6, H_ax_-1); 1.16 (m, 1H, H_α_-15); 1.20 (m, 1H, H_ax_-12); 1.26 (m, 1H, H_ax_-11); 1.29 (m, 1H, H_eq_-7); 1.32 (dd, 1H, ^3^*J*_9-11ax_ = 11.8, ^3^*J*_9-11eq_ = 3.3, H-9); 1.47 (m, 1H, H_ax_-6); 1.52 (m, 1H, H_eq_-11); 1.53 (m, 1H, H_eq_-6); 1.57 (m, 1H, H_ax_-7); 1.63 (m, 1H, H_ax_-2); 1.64 (m, 1H, H_eq_-12); 1.64 (m, 1H, H_eq_-2); 1.66 (m, 1H, H_β_-15); 1.70 (m, 1H, H_eq_-1); 1.71 (m, 1H, H_β_-16); 1.92 (m, 1H, H-13); 1.93 (m, 1H, H_α_-16); 2.04 (s, 3H, H-2′); 2.13 (s, 3H, H-21); 2.59 (td, 1H, ^3^*J*_17-13_ = 10.9, ^3^*J*_17-16α_ = 10.9, ^3^*J*_17-16β_ = 6.2, H-17); 4.48 (dd, 1H, ^3^*J*_3-2ax_ = 10.7, ^3^*J*_3-2eq_ = 5.6, H-3).

General procedure of compounds **3–8**

(a) Phosphoryl oxychloride (1 mL) was added to a solution of compound **1** or **2** (0.359 g or 0.403 g; 1.0 mmol) in 10 mL of anhydrous pyridine. The mixture was heated for 8 h under reflux, poured onto water, and extracted with chloroform (3 × 100 mL). The extract was washed with water (3 × 100 mL), dried over anhydrous calcium chloride, and evaporated under reduced pressure (water-jet pump). The residue was subjected to column chromatography on silica gel using eluent-petroleum ether-ethyl acetate (70:1→40:1). Compounds **6**, **7** were isolated in a mixture of products, with an overall yield of 87%. 

3-Oxo-22,23,24,25,26,27-hexanor-dammar-20(21)-in (**3**)

White solid, yield: 73%, mp: 161–162 °C, [α]^20^_D_ = +12 (c 0.05, CHCl_3_). ^13^C NMR (CDCl_3_, δ ppm): 15.33 (C30); 15.34 (C18); 16.04 (C19); 19.62 (C6); 21.03 (C29); 21.77 (C11); 24.77 (C12); 26.75 (C28); 29.93 (C16); 31.09 (C15); 31.55 (C17); 34.08 (C2); 34.71 (C7); 36.92 (C10); 39.95 (C1); 40.25 (C8); 47.39 (C4); 49.24 (C13); 49.26 (C14); 50.11 (C9); 55.34 (C5); 67.92 (C21); 89.04 (C20); 217.87 (C3). ^1^H NMR (CDCl_3_, δ ppm, *J* Hz): 0.81 (s, 3H, H-30); 0.95 (s, 3H, H-19); 1.01 (s, 3H, H-18); 1.04 (s, 3H, H-29); 1.08 (s, 3H, H-28); 1.15 (m, 1H, H_α_-15); 1.18 (qd, 1H, ^2^*J* = 12.4, ^3^*J*_12ax-13_ = 12.4, ^3^*J*_12ax-11ax_ = 12.4, ^3^*J*_12ax-11eq_ = 4.6, H_ax_-12); 1.32 (m, 1H, H_eq_-7); 1.35 (m, 1H, H_ax_-11); 1.37 (dd, 1H, ^3^*J*_5-6ax_ = 11.7, ^3^*J*_5-6eq_ = 2.0, H-5); 1.39 (dd, 1H, ^3^*J*_9-11ax_ = 12.5, ^3^*J*_9-11eq_ = 2.6, H-9); 1.46 (ddd, 1H, ^2^*J* = 13.2, ^3^*J*_1ax-2ax_ = 9.5, ^3^*J*_1ax-2eq_ = 7.8, H_ax_-1); 1.48 (m, 1H, H_eq_-6); 1.57 (m, 1H, H_ax_-6); 1.58 (m, 1H, H_eq_-11); 1.59 (m, 1H, H_ax_-7); 1.69 (m, 1H, H_β_-16); 1.71 (m, 1H, H_β_-15); 1.74 (ddd, 1H, ^3^*J*_13-12ax_ = 12.4, ^3^*J*_13-17_ = 12.2, ^3^*J*_13-12eq_ = 4.1, H-13); 1.87 (m, 1H, H_eq_-12); 1.95 (ddd, 1H, ^2^*J* = 13.2, ^3^*J*_1eq-2ax_ = 7.6, ^3^*J*_1eq-2eq_ = 4.7, H_eq_-1); 2.05 (d, 1H, ^4^*J*_21-17_ = 2.4, H-21); 2.11 (m, 1H, H_α_-16); 2.34 (dddd, 1H, ^3^*J*_17-13_ = 12.2, ^3^*J*_17-16α_ = 9.0, ^3^*J*_17-16β_ = 6.4, ^4^*J*_17-21_ = 2.4, H-17); 2.43 (ddd, 1H, ^2^*J* = 15.6, ^3^*J*_2eq-1ax_ = 7.8, ^3^*J*_2eq-1eq_ = 4.7, H_eq_-2); 2.51 (ddd, 1H, ^2^*J* = 15.6, ^3^*J*_2ax-1ax_ = 9.5, ^3^*J*_2ax-1eq_ = 7.6, H_ax_-2). 

3-Oxo-22,23,24,25,26,27-hexanor-dammar-20-chloro-20(21)-ene (**4**) 

White solid, yield: 7%, mp: 148–149 °C, [α]^20^_D_ = −30 (c 0.05, CHCl_3_). ^13^C NMR (CDCl_3_, δ ppm): 15.30 (C18); 15.96 (C30); 16.08 (C19); 19.66 (C6); 21.04 (C29); 21.80 (C11); 24.46 (C12); 26.75 (C28); 27.48 (C16); 31.28 (C15); 34.11 (C2); 34.79 (C7); 36.95 (C10); 39.99 (C1); 40.44 (C8); 45.14 (C13); 47.43 (C4); 49.40 (C14); 49.79 (C17); 50.28 (C9); 55.41 (C5); 111.59 (C21); 146.83 (C20); 218.00 (C3). ^1^H NMR (CDCl_3_, δ ppm, *J* Hz): 0.88 (s, 3H, H-30); 0.96 (s, 3H, H-19); 1.03 (s, 3H, H-18); 1.05 (s, 3H, H-29); 1.09 (s, 3H, H-28); 1.13 (m, 1H, H_ax_-12); 1.15 (m, 1H, H_α_-15); 1.31 (m, 1H, H_ax_-11); 1.33 (m, 1H, H_eq_-7); 1.38 (dd, 1H, ^3^*J*_5-6ax_ = 11.7, ^3^*J*_5-6eq_ = 2.0, H-5); 1.40 (dd, 1H, ^3^*J*_9-11ax_ = 12.6, ^3^*J*_9-11eq_ = 2.6, H-9); 1.46 (ddd, 1H, ^2^*J* = 13.2, ^3^*J*_1ax-2ax_ = 9.5, ^3^*J*_1ax-2eq_ = 7.8, H_ax_-1); 1.47 (m, 1H, H_eq_-6); 1.55 (m, 1H, H_eq_-11); 1.56 (m, 1H, H_ax_-6); 1.60 (m, 1H, H_ax_-7); 1.63 (m, 1H, H_eq_-12); 1.67 (m, 1H, H_β_-15); 1.74 (m, 1H, H_β_-16); 1.88 (ddd, 1H, ^3^*J*_13-12ax_ = 12.4, ^3^*J*_13-17_ = 10.6, ^3^*J*_13-12eq_ = 3.7, H-13); 1.89 (m, 1H, H_α_-16); 1.94 (ddd, 1H, ^2^*J* = 13.2, ^3^*J*_1eq-2ax_ = 7.6, ^3^*J*_1eq-2eq_ = 4.7, H_eq_-1); 2.43 (ddd, 1H, ^2^*J* = 15.6, ^3^*J*_2eq-1ax_ = 7.8, ^3^*J*_2eq-1eq_ = 4.7, H_eq_-2); 2.49 (td, 1H, ^3^*J*_17-13_ = 10.6, ^3^*J*_17-16α_ = 10.6, ^3^*J*_17-16β_ = 6.2, H-17); 2.51 (ddd, 1H, ^2^*J* = 15.6, ^3^*J*_2ax-1ax_ = 9.5, ^3^*J*_2ax-1eq_ = 7.6, H_ax_-2); 5.10 (d, 1H, ^2^*J* = 1.2, H_A_-21); 5.11 (d, 1H, ^2^*J* = 1.2, H_B_-21).

3*β*-Acetoxy-22,23,24,25,26,27-hexanor-dammar-20-chloro-20(21)-ene (**6**) 

White solid, yield: 80%, mp: 138–139 °C, [α]^20^_D_ = −110 (c 0.05, CHCl_3_). ^13^C NMR (CDCl_3_, δ ppm): 15.57 (C18); 16.02 (C30); 16.30 (C19); 16.50 (C29); 18.16 (C6); 21.28 (C11); 21.30 (C2′); 23.70 (C2); 24.42 (C12); 27.51 (C16); 27.98 (C28); 31.29 (C15); 35.38 (C7); 37.16 (C10); 37.90 (C4); 38.80 (C1); 40.54 (C8); 45.02 (C13); 49.41 (C14); 49.84 (C17); 50.83 (C9); 55.99 (C5); 80.86 (C3); 111.46 (C21); 146.93 (C20); 170.95 (C1′). ^1^H NMR (CDCl_3_, δ ppm, *J* Hz): 0.84 (dd, 1H, ^3^*J*_5-6ax_ = 11.5, ^3^*J*_5-6eq_ = 1.8, H-5); 0.85 (s, 3H, H-29); 0.86 (s, 3H, H-28); 0.86 (s, 3H, H-30); 0.88 (s, 3H, H-19); 0.99 (s, 3H, H-18); 1.05 (ddd, 1H, ^2^*J* = 11.8, ^3^*J*_1ax-2ax_ = 11.6, ^3^*J*_1ax-2eq_ = 4.7, H_ax_-1); 1.09 (qd, 1H, ^2^*J* = 12.4, ^3^*J*_12ax-13_ = 12.4, ^3^*J*_12ax-11ax_ = 12.4, ^3^*J*_12ax-11eq_ = 4.6, H_ax_-12); 1.12 (m, 1H, H_α_-15); 1.23 (qd, 1H, ^2^*J* = 12.7, ^3^*J*_11ax-9_ = 12.6, ^3^*J*_11ax-12ax_ = 12.6, ^3^*J*_12ax-11eq_ = 4.6, H_ax_-11); 1.29 (m, 1H, H_eq_-7); 1.32 (dd, 1H, ^3^*J*_9-11ax_ = 12.6, ^3^*J*_9-11eq_ = 2.6, H-9); 1.46 (m, 1H, H_ax_-6); 1.53 (m, 1H, H_eq_-6); 1.54 (m, 1H, H_eq_-11); 1.58 (m, 1H, H_ax_-7); 1.60 (m, 1H, H_eq_-12); 1.61 (m, 1H, H_ax_-2); 1.64 (m, 1H, H_eq_-2); 1.65 (m, 1H, H_β_-15); 1.71 (m, 1H, H_eq_-1); 1.74 (m, 1H, H_β_-16); 1.84 (ddd, 1H, ^3^*J*_13-12ax_ = 12.4, ^3^*J*_13-17_ = 10.6, ^3^*J*_13-12eq_ = 3.7, H-13); 1.88 (m, 1H, H_α_-16); 2.04 (s, 3H, H-2′); 2.47 (td, 1H, ^3^*J*_17-13_ = 10.6, ^3^*J*_17-16α_ = 10.6, ^3^*J*_17-16β_ = 6.2, H-17); 4.48 (dd, 1H, ^3^*J*_3-2ax_ = 10.7, ^3^*J*_3-2eq_ = 5.8, H-3); 5.08 (d, 1H, ^2^*J* = 1.2, H_A_-21); 5.10 (d, 1H, ^2^*J* = 1.2, H_B_-21).

3*β*-Acetoxy-22,23,24,25,26,27-hexanor-dammar-20(21)-in (**7**) 

White solid, yield: 7%, mp: 149–150 °C, [α]^20^_D_ = −61 (c 0.05, CHCl_3_). ^13^C NMR (CDCl_3_, δ ppm): 15.41 (C30); 15.65 (C18); 16.29 (C19); 16.51 (C29); 18.15 (C6); 21.27 (C11); 21.31 (C2′); 23.72 (C2); 24.75 (C12); 28.00 (C28); 29.98 (C16); 31.13 (C15); 31.61 (C17); 35.32 (C7); 37.16 (C10); 37.92 (C4); 38.82 (C1); 40.39 (C8); 49.17 (C13); 49.32 (C14); 50.71 (C9); 55.97 (C5); 67.80 (C21); 80.86 (C3); 89.24 (C20); 170.98 (C1′). ^1^H NMR (CDCl_3_, δ ppm, *J* Hz): 0.79 (s, 3H, H-30); 0.83 (dd, 1H, ^3^*J*_5-6ax_ = 11.5, ^3^*J*_5-6eq_ = 1.8, H-5); 0.85 (s, 3H, H-29); 0.85 (s, 3H, H-28); 0.88 (s, 3H, H-19); 0.97 (s, 3H, H-18); 1.05 (ddd, 1H, ^2^*J* = 11.8, ^3^*J*_1ax-2ax_ = 11.6, ^3^*J*_1ax-2eq_ = 4.7, H_ax_-1); 1.11 (m, 1H, H_eq_-15); 1.16 (m, 1H, H_ax_-12); 1.27 (m, 1H, H_eq_-7); 1.29 (m, 1H, H_ax_-11); 1.31 (dd, 1H, ^3^*J*_9-11ax_ = 12.6, ^3^*J*_9-11eq_ = 2.6, H-9); 1.46 (m, 1H, H_ax_-6); 1.53 (m, 1H, H_eq_-6); 1.56 (m, 1H, H_ax_-7); 1.58 (m, 1H, H_eq_-11); 1.61 (m, 1H, H_ax_-2); 1.64 (m, 1H, H_eq_-2); 1.68 (m, 1H, H_eq_-16); 1.70 (m, 1H, H_ax_-15); 1.71 (m, 1H, H_eq_-1); 1.73 (m, 1H, H-13); 1.85 (m, 1H, H_eq_-12); 2.03 (d, 1H, ^4^*J*_21-17_ = 2.4, H-21); 2.04 (s, 3H, H-2′); 2.10 (m, 1H, H_ax_-16); 2.32 (tdd, 1H, ^3^*J*_17-13_ = 10.6, ^3^*J*_17-16ax_ = 10.6, ^3^*J*_17-16eq_ = 6.2, ^4^*J*_17-21_ = 2.4, H-17); 4.48 (dd, 1H, ^3^*J*_3-2ax_ = 10.7, ^3^*J*_3-2eq_ = 5.8, H-3).

22,23,24,25,26,27-Hexanor-dammar-3,20-dichloro-20(21), 2(3)-diene (**8**)

White solid, yield: 8%, mp: 106–107 °C, [α]^20^_D_ = −28 (c 0.05, CHCl_3_). ^13^C NMR (CDCl_3_, δ ppm): 15.14 (C18); 15.98 (C30); 16.35 (C19); 20.01 (C29); 20.33 (C6); 21.71 (C11); 24.48 (C12); 27.54 (C16); 29.06 (C28); 31.30 (C15); 34.77 (C7); 36.42 (C10); 40.22 (C4); 40.35 (C8); 42.87 (C1); 45.17 (C13); 49.38 (C14); 49.44 (C9); 49.80 (C17); 54.37 (C5); 111.51 (C21); 122.07 (C2); 141.55 (C3); 146.94 (C20). ^1^H NMR (CDCl_3_, δ ppm, *J* Hz): 0.87 (s, 3H, H-30); 0.92 (s, 3H, H-19); 1.02 (s, 3H, H-18); 1.04 (s, 3H, H-29); 1.09 (qd, 1H, ^2^*J* = 12.4, ^3^*J*_12ax-13_ = 12.4, ^3^*J*_12ax-11ax_ = 12.4, ^3^*J*_12ax-11eq_ = 4.6, H_ax_-12); 1.14 (s, 3H, H-28); 1.15 (m, 1H, H_α_-15); 1.26 (dd, 1H, ^3^*J*_5-6ax_ = 11.0, ^3^*J*_5-6eq_ = 1.8, H-5); 1.29 (qd, 1H, ^2^*J* = 12.7, ^3^*J*_11ax-9_ = 12.6, ^3^*J*_11ax-12ax_ = 12.6, ^3^*J*_12ax-11eq_ = 4.6, H_ax_-11); 1.33 (m, 1H, H_eq_-7); 1.36 (dd, 1H, ^3^*J*_9-11ax_ = 12.6, ^3^*J*_9-11eq_ = 2.6, H-9); 1.50 (m, 1H, H_eq_-11); 1.55 (m, 1H, H_ax_-6); 1.58 (m, 1H, H_ax_-7); 1.58 (m, 1H, H_eq_-6); 1.62 (m, 1H, H_eq_-12); 1.65 (m, 1H, H_β_-15); 1.71 (dd, 1H, ^2^*J* = 16.7, ^3^*J*_1α-2_ = 2.2, H_α_-1); 1.75 (m, 1H, H_β_-16); 1.87 (ddd, 1H, ^3^*J*_13-12ax_ = 12.6, ^3^*J*_13-17_ = 10.9, ^3^*J*_13-12eq_ = 3.7, H-13); 1.89 (m, 1H, H_α_-16); 2.09 (dd, 1H, ^2^*J* = 16.7, ^3^*J*_1β-2_ = 6.8, H_β_-1); 2.49 (ddd, 1H, ^3^*J*_17-13_ = 10.9, ^3^*J*_17-16α_ = 10.1, ^3^*J*_17-16β_ = 6.3, H-17); 5.10 (d, 1H, ^2^*J* = 1.2, H_A_-21); 5.11 (d, 1H, ^2^*J* = 1.2, H_B_-21); 5.63 (dd, 1H, ^3^*J*_2-1β_ = 6.8, ^3^*J*_2-1α_ = 2.2, H-2).

(b) Phosphoryl pentachloride (1.04 g; 5.0 mmol) was added to a solution of compound **1** or **2** (0.359 g or 0.403 g; 1.0 mmol) in 10 mL of anhydrous chloroform. The mixture was heated for 8 h under reflux, poured onto water, and extracted with chloroform (3 × 100 mL). The extract was washed with water (3 × 100 mL), dried over anhydrous calcium chloride, and evaporated under reduced pressure (water-jet pump). The residue was subjected to column chromatography on silica gel using eluent-petroleum ether-ethyl acetate (70:1→40:1). Compounds **6**, **7** were isolated in a mixture of products, with an overall yield of 77%. 

3-Oxo-22,23,24,25,26,27-hexanor-dammar-20(21)-in (**3**)

White solid, yield: 14%, mp: 161–162 °C, [α]^20^_D_ = +12 (c 0.05, CHCl_3_).

3-Oxo-22,23,24,25,26,27-hexanor-dammar-20-chloro-20(21)-ene (**4**) 

White solid, yield: 71%, mp: 148–149 °C, [α]^20^_D_ = −30 (c 0.05, CHCl_3_). 

3-Chloro-22,23,24,25,26,27-hexanor-dammar-20-chloro-2(3),20(21)-diene (**5**)

White solid, yield: 8%, mp: 99–100 °C, [α]^20^_D_ = +18 (c 0.05, CHCl_3_). ^1^H NMR (CDCl_3_, δ ppm, *J* Hz): 0.87 (s, 3H, H-30); 0.92 (s, 3H, H-19); 1.02 (s, 3H, H-18); 1.04 (s, 3H, H-29); 1.09 (qd, 1H, ^2^*J* = 12.4, ^3^*J*_12ax-13_ = 12.4, ^3^*J*_12ax-11ax_ = 12.4, ^3^*J*_12ax-11eq_ = 4.6, H_ax_-12); 1.14 (s, 3H, H-28); 1.15 (m, 1H, H_eq_-15); 1.26 (dd, 1H, ^3^*J*_5-6ax_ = 11.0, ^3^*J*_5-6eq_ = 1.8, H-5); 1.29 (qd, 1H, ^2^*J* = 12.7, ^3^*J*_11ax-9_ = 12.6, ^3^*J*_11ax-12ax_ = 12.6, ^3^*J*_12ax-11eq_ = 4.6, H_ax_-11); 1.33 (m, 1H, H_eq_-7); 1.36 (dd, 1H, ^3^*J*_9-11ax_ = 12.6, ^3^*J*_9-11eq_ = 2.6, H-9); 1.50 (m, 1H, H_eq_-11); 1.55 (m, 1H, H_ax_-6); 1.58 (m, 1H, H_ax_-7); 1.58 (m, 1H, H_eq_-6); 1.62 (m, 1H, H_eq_-12); 1.65 (m, 1H, H_ax_-15); 1.71 (dd, 1H, ^2^*J* = 16.7, ^3^*J*_1α-2_ = 2.2, H_α_-1); 1.75 (m, 1H, H_eq_-16); 1.87 (ddd, 1H, ^3^*J*_13-12ax_ = 12.6, ^3^*J*_13-17_ = 10.9, ^3^*J*_13-12eq_ = 3.7, H-13); 1.89 (m, 1H, H_ax_-16); 2.09 (dd, 1H, ^2^*J* = 16.7, ^3^*J*_1β-2_ = 6.8, H_β_-1); 2.49 (ddd, 1H, ^3^*J*_17-13_ = 10.9, ^3^*J*_17-16ax_ = 10.1, ^3^*J*_17-16eq_ = 6.3, H-17); 5.10 (d, 1H, ^2^*J* = 1.2, H_A_-21); 5.11 (d, 1H, ^2^*J* = 1.2, H_B_-21); 5.63 (dd, 1H, ^3^*J*_2-1β_ = 6.8, ^3^*J*_2-1α_ = 2.2, H-2). ^13^C NMR (CDCl_3_, δ ppm): 15.14 (C18); 15.98 (C30); 16.35 (C19); 20.01 (C29); 20.33 (C6); 21.71 (C11); 24.48 (C12); 27.54 (C16); 29.06 (C28); 31.30 (C15); 34.77 (C7); 36.42 (C10); 40.22 (C4); 40.35 (C8); 42.87 (C1); 45.17 (C13); 49.38 (C14); 49.44 (C9); 49.80 (C17); 54.37 (C5); 111.51 (C21); 122.07 (C2); 141.55 (C3); 146.94 (C20).

(c) A mixture of **2** (0.403 g; 1.0 mmol), PCl_5_ (0.210 g; 1.0 mmol), and a catalytic amount of DMAP in dry pyridine (5 mL) was refluxed for 2 h. After completion of the reaction, the mixture was poured onto water. The precipitate that formed was collected by filtration and washed with water. The residue was purified by SiO_2_ column chromatography (eluent petroleum ether-ethyl acetate (70:1→40:1)).

3*β*-Acetoxy-22,23,24,25,26,27-hexanor-dammar-20(21)-in (**7**)

White solid, yield: 89%, mp: 149–150 °C, [α]^20^_D_ = −61 (c 0.05, CHCl_3_).

General procedure of compounds **9a–c**

To a solution of compound **3** (0.204 g; 0.6 mmol) in 12 mL of dry dioxane was added paraformaldehyde (0.180 g, 6 mmol), 0.72 mmol of corresponding amine (diethylamine, *N*-methylpiperazine, or morpholine), NaOAc (0.250 g; 3 mmol), and CuI (6 mg; 0.03 mmol). The reaction mixture was stirred under argon for 10 h at 60 °C. After the reaction was completed by TLC, the mixture was diluted with water and extracted with CHCl_3_ (3 × 20 mL), and the combined organic layer was washed with water, dried over CaCl_2_, and evaporated under reduced pressure. The residue was purified by SiO_2_ column chromatography (eluent petroleum ether-ethyl acetate (40:1→1:1), chloroform).

17-[3-(Diethylamino)-prop-1-yn-1-yl]-22,23,24,25,26,27-hexanor-dammar-3-one (**9a**)

White solid, yield: 78%, mp: 98–99 °C, [α]^20^_D_ = −15 (c 0.05, CHCl_3_). ^13^C NMR (CDCl_3_, δ ppm): 12.21 (C3”); 15.39 (C18); 15.39 (C30); 16.08 (C19); 19.64 (C6); 21.03 (C29); 21.85 (C11); 24.92 (C12); 26.77 (C28); 30.19 (C16); 31.06 (C15); 31.90 (C17); 34.10 (C2); 34.70 (C7); 36.94 (C10); 39.98 (C1); 40.26 (C8); 41.07 (C1′); 47.14 (C2″); 47.41 (C4); 49.19 (C14); 49.42 (C13); 50.17 (C9); 55.35 (C5); 73.42 (C21); 89.88 (C20); 217.94 (C3).^1^H NMR (CDCl_3_, δ ppm, *J* Hz): 0.80 (s, 3H, H-30); 0.95 (s, 3H, H-19); 1.01 (s, 3H, H-18); 1.04 (s, 3H, H-29); 1.08 (s, 3H, H-28); 1.11 (t, 6H, ^3^*J* = 7.1, H-3″); 1.12 (m, 1H, H_α_-15); 1.16 (m, 1H, H_ax_-12); 1.32 (m, 1H, H_ax_-11); 1.32 (m, 1H, H_eq_-7); 1.37 (dd, 1H, ^3^*J*_5-6ax_ = 11.7, ^3^*J*_5-6eq_ = 2.6, H-5); 1.39 (dd, 1H, ^3^*J*_9-11ax_ = 12.6, ^3^*J*_9-11eq_ = 2.6, H-9); 1.46 (ddd, 1H, ^2^*J* = 13.2, ^3^*J*_1ax-2ax_ = 9.6, ^3^*J*_1ax-2eq_ = 7.8, H_ax_-1); 1.48 (m, 1H, H_eq_-6); 1.56 (m, 1H, H_ax_-6); 1.57 (m, 1H, H_eq_-11); 1.59 (m, 1H, H_ax_-7); 1.63 (m, 1H, H_β_-16); 1.68 (m, 1H, H_β_-15); 1.69 (ddd, 1H, ^3^*J*_13-12ax_ = 11.9, ^3^*J*_13-17_ = 11.4, ^3^*J*_13-12eq_ = 3.7, H-13); 1.83 (m, 1H, H_eq_-12); 1.94 (ddd, 1H, ^2^*J* = 13.2, ^3^*J*_1eq-2ax_ = 7.6, ^3^*J*_1eq-2eq_ = 4.7, H_eq_-1); 2.07 (m, 1H, H_α_-16); 2.35 (ddd, 1H, ^3^*J*_17-13_ = 11.4, ^3^*J*_17-16α_ = 10.5, ^3^*J*_17-16β_ = 6.4, H-17); 2.43 (ddd, 1H, ^2^*J* = 15.6, ^3^*J*_2eq-1ax_ = 7.8, ^3^*J*_2eq-1eq_ = 4.7, H_eq_-2); 2.51 (ddd, 1H, ^2^*J* = 15.6, ^3^*J*_2ax-1ax_ = 9.6, ^3^*J*_2ax-1eq_ = 7.6, H_ax_-2); 2.60 (q, 4H, ^3^*J* = 7.1, H-2″); 3.45 (s, 2H, H-1′). ^15^N NMR (CDCl_3_, δ ppm): 46.35 (N1″).

17-[3-(Pyrrolidin-1-yl)-prop-1-yn-1-yl]-22,23,24,25,26,27-hexanor-dammar-3-one (**9b**)

White solid, yield: 87%, mp: 100–102 °C, [α]^20^_D_ = +67 (c 0.05, CHCl_3_). ^13^C NMR (CDCl_3_, δ ppm): 15.36 (C30); 15.39 (C18); 16.07 (C19); 19.63 (C6); 21.03 (C29); 21.79 (C11); 24.09 (C3″(4″)); 24.94 (C12); 26.75 (C28); 30.02 (C16); 31.09 (C15); 31.86 (C17); 34.08 (C2); 34.71 (C7); 36.94 (C10); 39.97 (C1); 40.29 (C8); 43.45 (C1′); 47.41 (C4); 49.27 (C13); 49.29 (C14); 50.12 (C9); 52.14 (C2″(5″)); 55.36 (C5); 73.53 (C21); 89.50 (C20); 217.83 (C3). ^1^H NMR (CDCl_3_, δ ppm, *J* Hz): 0.81 (s, 3H, H-30); 0.95 (s, 3H, H-19); 1.01 (s, 3H, H-18); 1.04 (s, 3H, H-29); 1.08 (s, 3H, H-28); 1.15 (m, 1H, H_α_-15); 1.19 (m, 1H, H_ax_-12); 1.32 (m, 1H, H_ax_-11); 1.32 (m, 1H, H_eq_-7); 1.38 (dd, 1H, ^3^*J*_5-6ax_ = 11.8, ^3^*J*_5-6eq_ = 2.7, H-5); 1.39 (dd, 1H, ^3^*J*_9-11ax_ = 12.7, ^3^*J*_9-11eq_ = 2.7, H-9); 1.47 (m, 1H, H_ax_-1); 1.48 (m, 1H, H_eq_-6); 1.56 (m, 1H, H_ax_-6); 1.57 (m, 1H, H_eq_-11); 1.59 (m, 1H, H_ax_-7); 1.65 (m, 1H, H_β_-16); 1.71 (m, 1H, H_β_-15); 1.74 (ddd, 1H, ^3^*J*_13-12ax_ = 11.9, ^3^*J*_13-17_ = 11.4, ^3^*J*_13-12eq_ = 3.7, H-13); 1.82 (m, 1H, H_eq_-12); 1.94 (ddd, 1H, ^2^*J* = 13.2, ^3^*J*_1eq-2ax_ = 7.6, ^3^*J*_1eq-2eq_ = 4.7, H_eq_-1); 1.96 (m, 4H, H-3″(4″)); 2.08 (m, 1H, H_α_-16); 2.36 (ddd, 1H, ^3^*J*_17-13_ = 11.4, ^3^*J*_17-16α_ = 10.5, ^3^*J*_17-16β_ = 6.4, H-17); 2.43 (ddd, 1H, ^2^*J* = 15.6, ^3^*J*_2eq-1ax_ = 7.8, ^3^*J*_2eq-1eq_ = 4.7, H_eq_-2); 2.51 (ddd, 1H, ^2^*J* = 15.6, ^3^*J*_2ax-1ax_ = 9.6, ^3^*J*_2ax-1eq_ = 7.6, H_ax_-2); 2.97 (m, 4H, H-2″(5″)); 3.63 (br.s, 2H, H-1′). 

17-[(3-Morpholino-prop-1-yn-1-yl]-22,23,24,25,26,27-hexanor-dammar-3-one (**9C**)

White solid, yield: 82%, mp: 105–106 °C, [α]^20^_D_ = −32 (c 0.05, CHCl_3_). ^13^C NMR (CDCl_3_, δ ppm): 15.38 (C30); 15.40 (C18); 16.07 (C19); 19.64 (C6); 21.04 (C29); 21.80 (C11); 24.94 (C12); 26.77 (C28); 30.12 (C16); 31.08 (C15); 31.91 (C17); 34.10 (C2); 34.71 (C7); 36.95 (C10); 39.98 (C1); 40.29 (C8); 47.41 (C4); 47.74 (C1′); 49.23 (C13); 49.24 (C14); 50.14 (C9); 52.05 (C2″(4″)); 55.37 (C5); 66.48 (C3″(5″)); 74.83 (C21); 89.30 (C20); 217.87 (C3). ^1^H NMR (CDCl_3_, δ ppm, *J* Hz): 0.81 (s, 3H, H-30); 0.95 (s, 3H, H-19); 1.00 (s, 3H, H-18); 1.04 (s, 3H, H-29); 1.08 (s, 3H, H-28); 1.13 (m, 1H, H_α_-15); 1.17 (m, 1H, H_ax_-12); 1.29 (m, 1H, H_ax_-11); 1.32 (m, 1H, H_eq_-7); 1.37 (dd, 1H, ^3^*J*_5-6ax_ = 11.7, ^3^*J*_5-6eq_ = 2.7, H-5); 1.39 (dd, 1H, ^3^*J*_9-11ax_ = 12.7, ^3^*J*_9-11eq_ = 2.7, H-9); 1.46 (m, 1H, H_ax_-1); 1.48 (m, 1H, H_eq_-6); 1.55 (m, 1H, H_ax_-6); 1.57 (m, 1H, H_eq_-11); 1.59 (m, 1H, H_ax_-7); 1.66 (m, 1H, H_β_-16); 1.70 (m, 1H, H_β_-15); 1.71 (ddd, 1H, ^3^*J*_13-12ax_ = 11.9, ^3^*J*_13-17_ = 11.4, ^3^*J*_13-12eq_ = 3.7, H-13); 1.83 (m, 1H, H_eq_-12); 1.94 (ddd, 1H, ^2^*J* = 13.2, ^3^*J*_1eq-2ax_ = 7.6, ^3^*J*_1eq-2eq_ = 4.7, H_eq_-1); 2.08 (m, 1H, H_α_-16); 2.34 (ddd, 1H, ^3^*J*_17-13_ = 11.4, ^3^*J*_17-16α_ = 10.5, ^3^*J*_17-16β_ = 6.4, H-17); 2.43 (ddd, 1H, ^2^*J* = 15.6, ^3^*J*_2eq-1ax_ = 7.8, ^3^*J*_2eq-1eq_ = 4.7, H_eq_-2); 2.51 (ddd, 1H, ^2^*J* = 15.6, ^3^*J*_2ax-1ax_ = 9.6, ^3^*J*_2ax-1eq_ = 7.6, H_ax_-2); 2.65 (m, 4H, H-2″(6″)); 3.36 (br.s, 2H, H-1′); 3.81 (m, 4H, H-3″(5″)).

General procedure of compounds **10** and **11**

Through a solution of compound **4** or **6** (0.377 g or 0.421 g; 1 mmol) in 50 mL of (CH_3_)_2_CO:H_2_O (20:1) solution, 2 eq. of ozone at room temperature was passed until the starting substance disappeared (TLC-control). The residue was subjected to column chromatography on SiO_2_ using eluent-petroleum ether-ethyl acetate (10:1→1:1), chloroform.

3-Oxo-22,23,24,25,26,27-hexanor-dammar-17-carboxylic acid (**10**)

White solid, yield: 94%, mp: 135-137 °C, [α]^20^_D_ = −48 (c 0.05, CHCl_3_). ^13^C NMR (CDCl_3_, δ ppm): 15.29 (C18); 15.55 (C30); 16.08 (C19); 19.64 (C6); 21.03 (C29); 21.71 (C11); 25.34 (C12); 26.26 (C16); 26.76 (C28); 31.53 (C15); 34.08 (C2); 34.87 (C7); 36.92 (C10); 39.97 (C1); 40.39 (C8); 45.56 (C17); 46.59 (C13); 47.42 (C4); 50.05 (C9); 50.06 (C14); 55.36 (C5); 181.26 (C20); 217.92 (C3). ^1^H NMR (CDCl_3_, δ ppm, *J* Hz): 0.87 (s, 3H, H-30); 0.95 (s, 3H, H-19); 1.03 (s, 3H, H-18); 1.04 (s, 3H, H-29); 1.09 (s, 3H, H-28); 1.20 (ddd, 1H, ^2^*J* = 12.1, ^3^*J*_15α-16β_ = 8.6, ^3^*J*_15α-16α_ = 2.4, H_α_-15); 1.25 (qd, 1H, ^2^*J* = 12.6, ^3^*J*_12ax-13_ = 12.6, ^3^*J*_12ax-11ax_ = 12.6, ^3^*J*_12ax-11eq_ = 4.6, H_ax_-12); 1.32 (qd, 1H, ^2^*J* = 12.2, ^3^*J*_11ax-9_ = 12.2, ^3^*J*_11ax-12ax_ = 12.2, ^3^*J*_11ax-12eq_ = 3.7, H_ax_-11); 1.35 (m, 1H, H_eq_-7); 1.38 (dd, 1H, ^3^*J*_5-6ax_ = 11.7, ^3^*J*_5-6eq_ = 2.8, H-5); 1.41 (dd, 1H, ^3^*J*_9-11ax_ = 12.5, ^3^*J*_9-11eq_ = 2.6, H-9); 1.46 (ddd, 1H, ^2^*J* = 13.2, ^3^*J*_1ax-2ax_ = 9.6, ^3^*J*_1ax-2eq_ = 7.8, H_ax_-1); 1.48 (m, 1H, H_eq_-6); 1.56 (m, 1H, H_eq_-11); 1.57 (m, 1H, H_ax_-6); 1.61 (m, 1H, H_ax_-7); 1.72 (ddd, 1H, ^2^*J* = 12.1, ^3^*J*_15β-16β_ = 10.8, ^3^*J*_15β-16α_ = 8.8, H_β_-15); 1.81 (m, 1H, H_eq_-12); 1.92 (m, 1H, H_β_-16); 1.94 (ddd, 1H, ^2^*J* = 13.2, ^3^*J*_1eq-2ax_ = 7.6, ^3^*J*_1eq-2eq_ = 4.7, H_eq_-1); 1.98 (ddd, 1H, ^3^*J*_13-12ax_ = 12.6, ^3^*J*_13-17_ = 11.5, ^3^*J*_13-12eq_ = 4.1, H-13); 2.00 (m, 1H, H_α_-16); 2.43 (ddd, 1H, ^2^*J* = 15.6, ^3^*J*_2eq-1ax_ = 7.8, ^3^*J*_2eq-1eq_ = 4.7, H_eq_-2); 2.47 (ddd, 1H, ^3^*J*_17-13_ = 11.5, ^3^*J*_17-16α_ = 10.5, ^3^*J*_17-16β_ = 6.5, H-17); 2.51 (ddd, 1H, ^2^*J* = 15.6, ^3^*J*_2ax-1ax_ = 9.6, ^3^*J*_2ax-1eq_ = 7.6, H_ax_-2).

3β-Acetoxy-22,23,24,25,26,27-hexanor-dammar-17-carboxylic acid (**11**)

White solid, yield: 91%, mp: 147–148 °C, [α]^20^_D_ = +5 (c 0.05, CHCl_3_). ^13^C NMR (CDCl_3_, δ ppm): 15.56 (C18); 15.61 (C30); 16.30 (C19); 16.49 (C29); 18.14 (C6); 21.19 (C11); 21.29 (C2′); 23.69 (C2); 25.28 (C12); 26.25 (C16); 27.97 (C28); 31.54 (C15); 31.92 (C2); 35.46 (C7); 37.13 (C10); 37.90 (C4); 38.80 (C1); 40.50 (C8); 45.61 (C13); 46.51 (C14); 50.07 (C17); 50.63 (C9); 55.99 (C5); 80.85 (C3); 170.99 (C1′). ^1^H NMR (CDCl_3_, δ ppm, *J* Hz): 0.81 (dd, 1H, ^3^*J*_5-6ax_ = 11.5, ^3^*J*_5-6eq_ = 1.8, H-5); 0.84 (s, 3H, H-29); 0.87 (s, 3H, H-28); 0.90 (s, 3H, H-30); 0.92 (s, 3H, H-19); 0.99 (s, 3H, H-18); 1.02 (ddd, 1H, ^2^*J* = 11.8, ^3^*J*_1ax-2ax_ = 11.6, ^3^*J*_1ax-2eq_ = 4.7, H_ax_-1); 1.09 (qd, 1H, ^2^*J* = 12.4, ^3^*J*_12ax-13_ = 12.4, ^3^*J*_12ax-11ax_ = 12.4, ^3^*J*_12ax-11eq_ = 4.6, H_ax_-12); 1.12 (m, 1H, H_α_-15); 1.23 (qd, 1H, ^2^*J* = 12.7, ^3^*J*_11ax-9_ = 12.6, ^3^*J*_11ax-12ax_ = 12.6, ^3^*J*_12ax-11eq_ = 4.6, H_ax_-11); 1.29 (m, 1H, H_eq_-7); 1.32 (dd, 1H, ^3^*J*_9-11ax_ = 12.6, ^3^*J*_9-11eq_ = 2.6, H-9); 1.46 (m, 1H, H_ax_-6); 1.53 (m, 1H, H_eq_-6); 1.54 (m, 1H, H_eq_-11); 1.58 (m, 1H, H_ax_-7); 1.60 (m, 1H, H_eq_-12); 1.61 (m, 1H, H_ax_-2); 1.64 (m, 1H, H_eq_-2); 1.65 (m, 1H, H_β_-15); 1.71 (m, 1H, H_eq_-1); 1.74 (m, 1H, H_β_-16); 1.84 (ddd, 1H, ^3^*J*_13-12ax_ = 12.4, ^3^*J*_13-17_ = 10.6, ^3^*J*_13-12eq_ = 3.7, H-13); 1.88 (m, 1H, H_α_-16); 2.04 (s, 3H, H-2′); 2.48 (td, 1H, ^3^*J*_17-13_ = 10.6, ^3^*J*_17-16α_ = 10.6, ^3^*J*_17-16β_ = 6.2, H-17); 4.50 (dd, 1H, ^3^*J*_3-2ax_ = 10.7, ^3^*J*_3-2eq_ = 5.8, H-3).

Procedure of compound **12**

Through a solution of the acid **11** (0.406 g; 1 mmol) in 50 mL of anhydrous CH_2_Cl_2_, 2 eq. of ozone at –40 °C was passed until the starting substance disappeared (TLC-control). The resulting acid chloride was dissolved in anhydrous CH_2_Cl_2_ (30 mL) and treated with pyrrolidine (0.150 g; 1.5 mmol) and three drops of Et_3_N. The mixture was stirred at room temperature for 3 h, washed with 5% HCl solution (2 × 100 mL) and H_2_O (100 mL), dried over CaCl_2_, and the solvent was removed under reduced pressure. The product was purified by column chromatography with SiO_2_ using CHCl_3_ and a mixture of CHCl_3_–EtOH (100:1) as eluents.

3β-Acetoxy-22,23,24,25,26,27-hexanor-dammar-17-*N*-pyrrolidine amide (**12**)

White solid, yield: 89%, mp: 114–115 °C, [α]^20^_D_ = +23 (c 0.05, CHCl_3_). ^13^C NMR (CDCl_3_, δ ppm): 15.69 (C18); 16.01 (C30); 16.34 (C19); 16.50 (C29); 18.15 (C6); 21.26 (C11); 21.34 (C2′); 23.70 (C2); 24.36 (C4″); 25.54 (C12); 26.11 (C3″); 26.60 (C16); 27.97 (C28); 31.66 (C15); 35.53 (C7); 37.12 (C10); 37.89 (C4); 38.80 (C1); 40.53 (C8); 45.06 (C17); 45.81 (C5″); 45.89 (C13); 46.48 (C2″); 49.63 (C14); 50.77 (C9); 55.99 (C5); 80.94 (C3); 171.08 (C1′); 175.10 (C20). ^15^N NMR (CDCl_3_, δ ppm): 127.29 (N1″). ^1^H NMR (CDCl_3_, δ ppm, *J* Hz): 0.83 (dd, 1H, ^3^*J*_5-6ax_ = 11.7, ^3^*J*_5-6eq_ = 2.6, H-5); 0.84 (s, 3H, H-29); 0.85 (s, 3H, H-28); 0.86 (s, 3H, H-19); 0.87 (s, 3H, H-30); 1.01 (s, 3H, H-18); 1.04 (td, 1H, ^2^*J* = 12.2, ^3^*J*_1ax-2ax_ = 12.2, ^3^*J*_1ax-2ax_ = 5.6, H_ax_-1); 1.15 (m, 1H, H_α_-15); 1.17 (m, 1H, H_ax_-12); 1.27 (m, 1H, H_ax_-11); 1.30 (m, 1H, H_eq_-7); 1.57 (m, 1H, H_ax_-7); 1.31 (dd, 1H, ^3^*J*_9-11ax_ = 11.8, ^3^*J*_9-11eq_ = 3.3, H-9); 1.47 (m, 1H, H_ax_-6); 1.49 (m, 1H, H_eq_-11); 1.53 (m, 1H, H_eq_-6); 1.57 (m, 1H, H_ax_-7); 1.62 (m, 1H, H_ax_-2); 1.64 (m, 1H, H_eq_-12); 1.64 (m, 1H, H_eq_-2); 1.71 (m, 1H, H_eq_-1); 1.74 (m, 1H, H_β_-15); 1.77 (m, 1H, H_β_-16); 1.84 (m, 2H, H-4″); 1.92 (m, 1H, H_α_-16); 1.92 (m, 2H, H-3″); 3.44 (s, 3H, H-2″); 2.16 (m, 1H, H-13); 2.52 (td, 1H, ^3^*J*_17-13_ = 10.9, ^3^*J*_17-16α_ = 10.9, ^3^*J*_17-16β_ = 6.2, H-17); 3.44 (s, 3H, H-2″); 3.47 (m, 1H, H-5″); 4.47 (dd, 1H, ^3^*J*_3-2ax_ = 10.7, ^3^*J*_3-2eq_ = 5.6, H-3).

## 3. Discussion

We used hollongdiones **1** and **2** (for X-ray crystal structure, see Figure 3, for NMR data see Supplementary Material Figures S1–S8) in the reactions with POCl_3_ and PCl_5_ (Scheme 1). As a result, a series of derivatives **3–8** was obtained and identified. 

Alkyne **3** was synthesized by the interaction of hollongdione **1** with POCl_3_ in pyridine, with the yield of 73% after purification by column chromatography, while the vinyl chloride **4** was also isolated as the minor (7%) product. The alkyne synthesis can be explained by dehydrohalogenation of a *gem*-dihalogen intermediate formation during thermal activation under reaction conditions; vinyl chloride’s mechanism includes the cleavage of one HCl molecule step. It is worth noting that the composition of the products was different when lupane triterpenoids were used as the initial scaffolds: the presence of a molecule with a double C=C bond and tetrahydrofuran ring and a compound with a trichloroacetyl group besides the main alkyne derivative were confirmed, which differs from previous results [15]. Moreover, the *abeo*-lupene fragment was obtained under the action of POCl_3_ in pyridine [29,30]. 

At the same time, the reaction of **1** with PCl_5_ in CHCl_3_ led to the vinyl chloride **4** as the main product (71%); alkyne **3** was obtained among the by-products (14%), and ring A was transformed into a 3-chloro-2(3)-ene derivative **5** (8%), which could be explained as a result of the action of the Vilsmeyer–Haack reagent on the C3-oxo-group. The application of phosphorus oxychloride along with DMF to friedelin as the substrate with chlorofriedel-2 and 3-chlorofriedel-3-enes as the products was demonstrated [31]. 

**Scheme 1:**
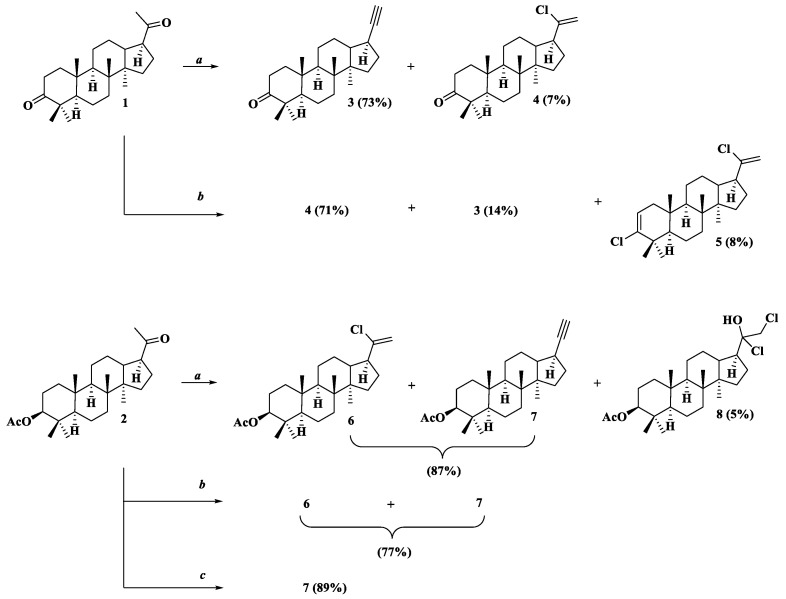
Reagents and conditions: a. POCl_3_/Py, ∆, 8 h; b. PCl_5_/CHCl_3_, ∆, 8 h; c. PCl_5_/Py, DMAP (cat.), ∆, 2 h.

The protection of the C3 position by the acetoxy group (compound **2**) made it possible to avoid the formation of *A*-ring by-products. In the case of reaction with POCl_3_, compounds **6** and **7** were isolated only as the mixture of products, with the overall yield of 87%, and **8** became a new by-product (5%). The overall yield of compounds **6** and **7** reached 77% when the halogenating reagent was PCl_5_ (Conditions *b* at Scheme 1). Previously, the optimization of the synthetic strategy for the lup-20(30)-yne preparation by adding a catalytic amount of DMAP was proposed [30]. An attempt to streamline the synthesis in this way was also successful, yielding only compound **7** with 89% after purification by column chromatography.

**Figure 4:**
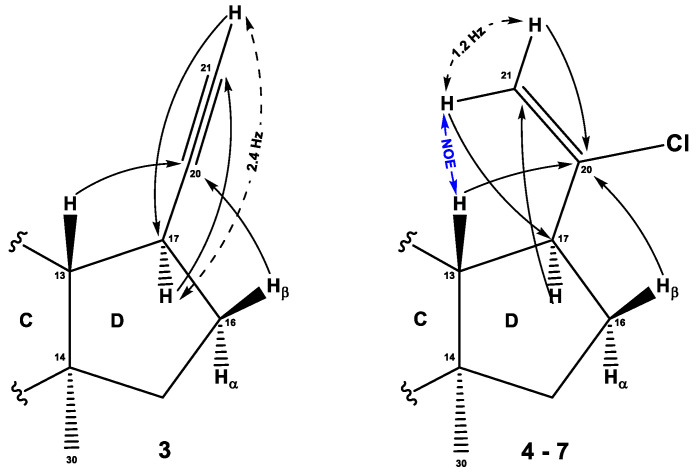
The key NMR assignments and significant {^1^H, ^13^C} HMBC, {^1^H, ^1^H} COSY, and NOESY correlations of compounds **3**–**7**.

The structure of all compounds was determined by ^1^H and ^13^C NMR spectroscopy data. For compound **3**, the presence of the oxo-group at the *C*3 position was confirmed by HMBC cross-peaks of *gem*-dimethyl groups *C*28 (δ_H_ 1.08 ppm) and *C*29 (δ_H_ 1.04 ppm) along with δ_C_ 217.87 ppm. Also, HMBC cross-peaks of methylene protons at *C*1 and *C*2 were observed for the *C*3-oxo-group. For the ethynyl substituent at *C*17 with δ_C_ 89.04 ppm of the quaternary carbon atom and δ_C_ 67.33 ppm and δ_H_ 2.05 ppm of the methine group, a number of interactions were observed, confirming the position of substitution. Thus, HMBC proton cross-peaks of *H*-13, *H*-17, and *H_β_*-16, with quaternary position and cross-peak *H*-17/*C*21 were confirmed. In addition, the acetylene proton signal has a doublet with the value *^4^J* = 2.4 Hz, with a methine proton *H*-17, which is confirmed in the spectrum COSY-DQF and its response splitting (Figure 4), (Supplementary Material Figures S9–S16). 

For the vinyl chloride group in compounds **4**–**7** with characteristic values δ_C_ ~147 ppm of the quaternary carbon atom *C*20 and δ_C_ ~112 ppm for the terminal methylene group, HMBC interactions with cycle *D* protons were observed, which confirms the position of substitution *C*17. In particular, HMBC cross-peaks of proton *H_α_*-16, *H_β_*-16, and *H*-13 with quaternary carbon atom *C*20 and HMBC cross-peaks of terminal protons with carbon signal *C*17 at δ_C_ ~50 ppm were observed. Additionally, in the NOESY spectrum, cross-peaks were observed between one of the terminal protons with *H_eq_*-12, *H*-13 and *H*-17. The presence of acetate in the *C*3 position of cycle *A* for compound **4** is confirmed by the HMBC cross peak of the doublet-doublet signal *H*-3 (δ_H_ 4.48 ppm) with a carboxyl group signal at δ_C_ 170.95 ppm (Figure 4).

**Figure 5:**
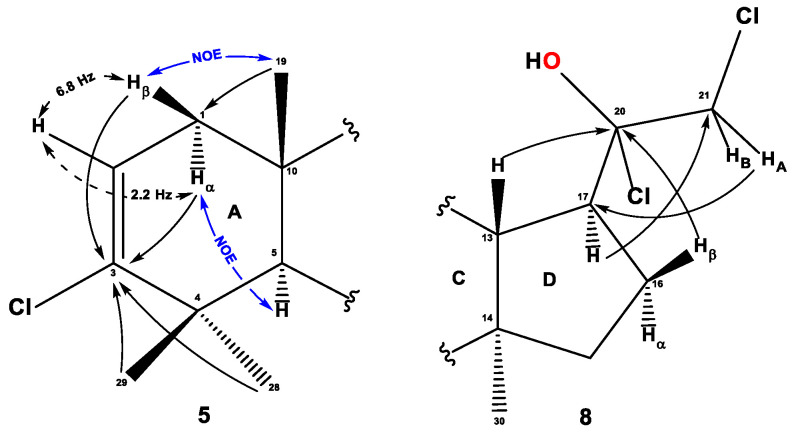
The key NMR assignments and significant {^1^H, ^13^C} HMBC, {^1^H, ^1^H} COSY, and NOESY correlations of compounds **5**, **8**. The oxygen atom is highlighted in red color in the structure of compound **8**.

For compound **5**, the presence of *C*2 = C3 double bond in cycle *A* was confirmed on the basis of HMBC cross-peaks *gem*-dimethyl *C*28 and *C*29 groups (δ_H_ 1.14 and 1.04 ppm, respectively) with a quaternary *Cl*-bearing position at δ_C_ 141.55 ppm, and *H*-2 protons (δ_H_ 5.63 ppm) and *α,β*-protons of the methylene group *C*1 (δ_H_ 1.71, 2.09 ppm). A double bond methine proton *H*-2 (δ_H_ 5.63 ppm) was represented by doublet-doublet splitting with *^3^J* = 6.8 and 2.2 Hz on neighboring methylene protons at *C*1. The position of the carbon signal *C*1 at δ_C_ 42.87 ppm was localized on the basis of HMBC cross-peaks with methine protons *H*-5 (δ_H_ 1.26 ppm) and *H*-9 (δ_H_ 1.36 ppm) and methyl group protons at *C*19 (δ_H_ 0.92 ppm). The *α*-orientation of protons at *C*1 (δ_H_ 1.71 ppm) was confirmed by NOESY cross-peaks with *H*-5 and *H*-9; for the protons *H_β_*-1, NOE interactions with *H_3_*-19 and *H_eq_*-11 (δ_H_ 1.50 ppm) protons were also observed (Figure 5), (Supplementary Material Figures S17–S40).

The formation of a 1,2-dichloro-1-hydroxyethyl group at the *C*17 of compound **8** is confirmed by the characteristic ^13^C NMR values: δ_C_ 97.32 ppm for the quaternary position *C*20 and δ_C_ 54.62 ppm for the methylene group *C*21. For *OH, Cl*-bearing quaternary carbon, three-bond HMBC correlations with protons *H_α_*-16, *H_β_*-16 (δ_H_ 2.00 and 1.76 ppm, respectively), and *H*-13 (δ_H_ 1.95 ppm) are observed. The HMBC spectrum also contains cross peaks of diastereotopic methylene protons at *C*21 (δ_H_ 4.01 and 4.05 ppm) with carbons *C*20 and *C*17 (δ_C_ 51.18 ppm), and the protons themselves in the ^1^H NMR spectrum are presented as two doublets with a geminal constant of 12.2 Hz.

**Figure 6:**
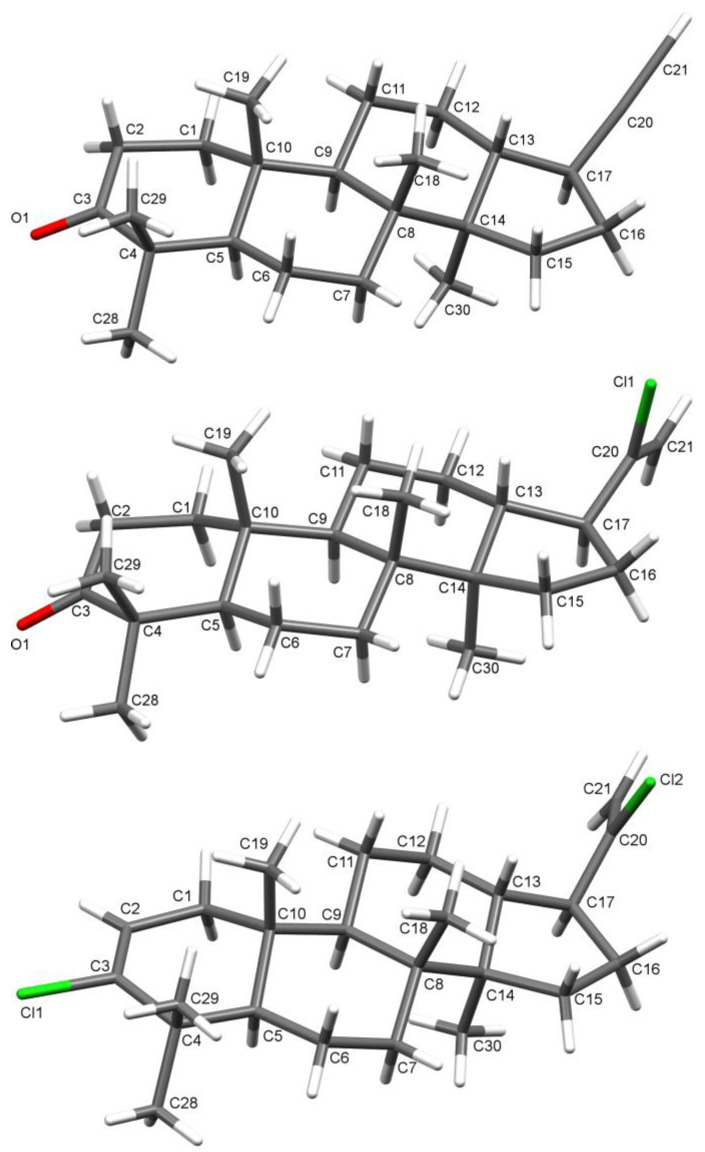
Molecular structures of compounds **3**, **4**, and **5**.

The molecular structures of 3-oxo-22,23,24,25,26,27-hexanor-dammar-20(21)-in **3**, 3-oxo-22,23,24,25,26,27-hexanor-dammar-20-chloro-20(21)-en **4,** and 22,23,24,25,26,27-hexanor-dammar-3,20-dichloro-20(21), 2(3)-dien **5** were determined by X-ray analysis (Figure 6). All the compounds have typical four-cycle moiety as in hollongdione [20], including three six- and one five-membered trans-fused cycles. All the six-membered cycles in **3**, **4**, and **5** adopt chair conformation, except the chlorine substituted one in **5** with double bond C2 = C3 equaling 1.307(4) Å, which has a distorted half-chair conformation. The five-membered cycles show twist conformation in all the compounds. The length of the C≡C bond in **3** slightly differs, being the same within the limit of experimental accuracy (Table 1) and very close to the average literature value of 1.174(11) [32]. The alkynyl substituent on C17 in **3** is not ideally linear, so the angle C17C20C21 is not the 180° that is usual for such moieties. The bond lengths and orientation of the vinyl chloride group on C17 in **4** and **5**, describing by torsion angle C16C17C20Cl1(Cl2), are the same in both structures (Table 1). There are not any specific intermolecular interactions in the crystals under consideration. The crystal structure of compounds is stabilized by van der Waals interactions. The main XRD data and experimental details for compounds **2**–**5** are presented in Table 2.

It is known that alkynes are simple and valuable precursors that act as electrophiles due to the presence of π-bonds and can be successfully utilized for the construction of various classes of carbocycles, heterocycles, complex molecular architectures, and natural products [33]. For example, the terminal carbon–carbon triple bond is an activating functional group that is required to render the substrate active in the Mannich reaction. In the last decade, new propargylamines were synthesized by the interaction of 19- and 28-alkynyltriterpenoids with *N*-methylpiperazine [34]. Propargylated oleanolic and glycyrrhetic acids were involved in aminomethylation by the Mannich reaction [35]. Moreover, this type of modification has been studied on the basis of steroid alkynes, and the interest keeps growing [36]. 

**Scheme 2:**
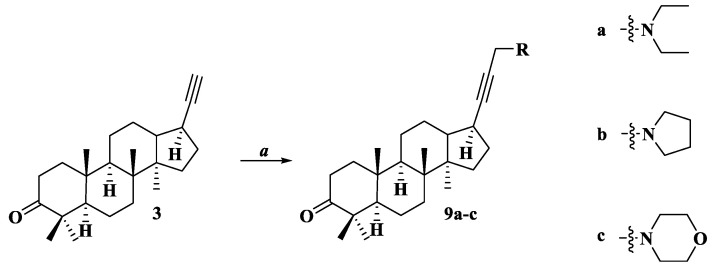
Reagents and conditions: a. diethylamine, pyrrolidine, or morpholine, paraformaldehyde, NaOAc, CuI, 1,4-dioxane, 10 h, 60 °C.

Taking into account the above, we involved alkyne **3** in the Mannich reaction with diethylamine, pyrrolidine, or morpholine, giving the conjugates **9a**–**c** in the yields of 78–87% (Scheme 2). 

The formation of aminomethylated products **9a**–**c** is confirmed by NMR spectra due to the presence of methylene group signals in the range δ_C_ 41.07 ÷ 47.74 ppm (δ_H_ 3.36 ÷ 3.62 ppm), for which HMBC cross-peaks with carbon signals of the acetylene group (δ_C_ 73.42÷74.89 and δ_C_ 89.28 ÷ 89.88 ppm) are observed. In addition, cross-peaks of protons of the *α*-position of amines (δ_H_ 2.60 ÷ 2.69 ppm) with the carbon signal of the newly formed methylene group *C*1′ are observed in the HMBC spectrum (Supplementary Material Figures S41–S63).

In the next stage of our research, we decided to involve the hollongdione vinyl chloride for transformation to carboxylic acid. It is known that androstane methylketones are transformed to carboxylic acids by a haloform reaction [37,38]. On the other hand, dichlorovinyl insecticides by ozone oxidation in water-containing solvents afford carboxy-derivatives [39].

**Scheme 3:**
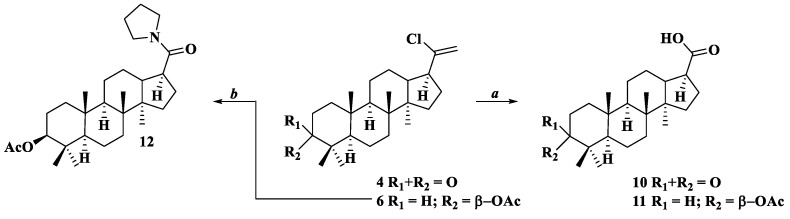
Reagents and conditions*:* a. O_3,_ (CH_3_)_2_C(O):H_2_O (20:1), rt; b. *i* O_3_, CH_2_Cl_2_, −40 °C, *ii* pyrrolidine, CH_2_Cl_2_, rt, 3 h.

Vinyl chloride is a well-known monomer for the synthesis of copolymers [40], but no instances of its oxidation to acid have been found in the case of triterpenoid or steroid scaffolds. We propose here a new approach for the synthesis of acids from C17-methylketones. For this, vinyl chlorides **4** and **6** were oxidized with ozone in acetone-water medium to give acids **10** and **11**. So, these acids could be called “hollongdionoic acid”-like, androstane-17-carboxylic acids were given the trivial name “etianic acids” [41]. One-pot synthesis of amide **12** in the yield of 89% was realized by the oxidation of vinyl chloride **6** in CH_2_Cl_2_ followed by the addition of pyrrolidine (Scheme 3).

The formation of a carboxyl group at the *C*17 position of compound **10** was established based on the presence of a signal at δ_C_ 181.26 ppm in the ^13^C NMR spectrum. The HMBC spectrum showed cross-peaks of the *H*-13, *H_β_*-16, and *H*-17 (δ_H_ 1.98, 1.92, and 2.47 ppm, respectively) protons of cycle *D* with a quaternary carbon signal at δ_C_ 181.26 ppm, (Supplementary Material Figures S64–S79).

## 4. Materials and Methods

NMR spectra were recorded on a Bruker “*Avance-III*” 500 MHz spectrometer (Bruker, Billerica, MA, USA), (500, 125, and 50 MHz for ^1^H, ^13^C, and ^15^N, respectively, δ, ppm, *J*, Hz) in CDCl_3_ with tetramethylsilane as the internal standard. Mass spectra were obtained on a liquid chromatograph–mass spectrometer LCMS-2010 EV (Shimadzu, Kyoto, Japan). Melting points were detected on the micro table “Rapido PHMK05” (Nagema, Dresden, Germany). Optical rotations were measured on the polarimeter “Perkin-E lmer 241 MC” (Perkin Elmer, Waltham, MA, USA) in a tube length of 1 dm. Thin-layer chromatography analyses were performed on Sorbfil plates (Sorbpolimer, Krasnodar, Russian Federation) using the solvent system chloroform-ethyl acetate, 40:1. Substances were detected by 10% H_2_SO_4_ with subsequent heating to 100–120 °C for 2–3 min. The X-ray diffraction experiments for compounds **2**–**5** were carried out at 296(2) K on a Bruker KAPPA APEX II diffractometer (graphite-monochromated Mo Kα radiation). Reflection intensities were corrected for absorption by the SADABS-2016 program [42]. The structure of compounds was solved by direct methods using the SHELXT-2014 program [43] and refined by the anisotropic (isotropic for all H atoms) full-matrix least-squares method against *F*^2^ of all reflections by SHELXL-2018 [44]. The positions of the hydrogen atoms were calculated geometrically and refined in the riding model. The asymmetric unit of **2** contained four molecules, the unit of **3**—two molecules, and structure of this compound was refined as a twin. Crystallographic data for **2**–**5** have been deposited at the Cambridge Crystallographic Data Centre as supplementary publication Nos. CCDC 2315012, 2315013, 2315014, and 2350691. A copy of the data can be obtained, free of charge, on application to the CCDC, 12 Union Road, Cambridge CB21EZ, UK (fax: +44 122 3336033 or e-mail: deposit@ccdc.cam.ac.uk; internet: www.ccdc.cam.ac.uk, accessed on 14 October 2023). Compound **1** was obtained according to the method described previously [20]. 

## 5. Conclusions

Hollongdione is the naturally occurring “triterpenoid-steroid” hybrid. Its design and synthesis is deemed to be very interesting from the perspective of potent activities. Its reactivity with an emphasis on dehydrohalogenation reactions such as phosphorus-chlorine derivatives (POCl_3_, PCl_5_) was studied for the first time. As a result, compounds with *C*17 alkynyl and vinyl chloride substituents were synthesized, the structures of which were confirmed by NMR spectra and X-ray analysis. The choice of a dehydrohalogenating agent made it possible to regulate the structure, composition, and the yield of the products. Hollongdione C17-alkyne was successfully transformed into aminomethylated products by the Mannich reaction. An opportunity for a one-stage pathway from hollongdione vinyl chloride to the carboxylic acid with the following amide preparation is also demonstrated. 

## Figures and Tables

**Figure 3:**
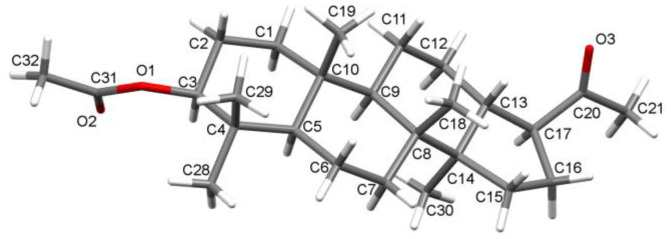
The molecular structure of compound **2**.

**Table 1:** Selected geometric parameters of compounds **3**–**5**.

	3	4	5
C20-C21	1.157 (13)/1.136 (16) *	1.312 (4)	1.315 (5)
C17C20C21	175.3 (11)/175.2 (15)	−	−
C20-Cl1(Cl2)		1.750 (3)	1.748 (4)
C20-C17		1.493 (4)	1.496 (4)
C16C17C20Cl1 (Cl2)		−55.9 (3)	−53.1 (3)

* geometrical parameters for two independent molecules in **3**.

**Table 2:** XRD data and experimental details for compounds **2**–**5**.

	2	3	4	5
Empirical formula	C_26_H_42_O_3_	C_24_H_35_O	C_24_H_37_ClO	C_24_H_36_Cl_2_
Formula weight	402.59	339.52	376.98	395.43
Crystal system	Monoclinic	Monoclinic	Triclinic	Monoclinic
Space group	*P*2_1_	*P*2_1_	*P*1	*C*2
Unit cell dimensions *a*, *b*, *c* (Å)	25.332(2), 7.6508(5), 25.543(2)	7.5834(6), 20.548(2), 12.9331(9)	6.3333(3), 7.4257(5), 11.8452(8)	13.880(1), 6.3288(4), 24.409(2)
α, β, γ (°)	90, 107.232(3), 90	90, 90.020(4), 90	89.026(2), 84.321(2), 69.960(2)	90, 97.004(4), 90
Volume (Å^3^)	4728.1(6)	2015.3(3)	520.69(6)	2128.1(3)
*Z*	8	4	1	4
Density (calcd.) (Mg.mm^−3^)	1.131	1.119	1.202	1.234
F(000)	1776	748	206	856
Absorption coefficient (mm^−1^)	0.07	0.07	0.19	0.311
Crystal size (mm^3^)	1.00 × 0.08 × 0.05	1.00 × 0.45 × 0.01	1.00 × 0.72 × 0.20	1.00 × 0.30 × 0.02
*T*_min_, *T*_max_	0.832, 0.928	0.770, 0.928	0.764, 0.813	0.762, 0.801
Θ range for data collection	0.8–26.5	1.0–26.1	2.9–33.5	0.8–27.1
Reflections collected	101,280	19,801	14,660	35,210
Independent reflections [*R*_int_]	19,379 [0.088]	7303 [0.095]	6349 [0.024]	4471 [0.049]
Observed [*I* > 2 *σ* (*I*)] reflections	11,585	4090	5142	4097
Completeness to θ = 25° (%)	99.9	99.7	99.9	98.8
Data/restraints/parameters	193,79/1/1073	7303/2/462	6349/3/240	4471/1/240
Goodness-of-fit on *F*^2^	1.01	0.99	1.02	1.107
Final R1, [*I >* 2*σ(I)*]	0.061	0.064	0.046	0.041
Final wR2 (all data)	0.155	0.148	0.138	0.109
Largest diff. peak/hole e.Å^−3^	0.20, −0.21	0.18, −0.17	0.36, −0.27	0.27, −0.201
Absolute structure parameter	−0.1(5)	0.5(10)	0.039(17)	0.029(17)
CCDC	2,315,012	2,315,013	2,315,014	2,350,691

## Data Availability

Data available within the article or its Supplementary Materials.

## Supplementary Materials

The following supporting information can be downloaded at: https://www.mdpi.com/article/10.3390/ijms25158356/s1.
