# Supplementary material for: One-Stage Pathway from Hollongdione to C17-Alkyne and Vinyl Chloride Following Mannich Bases and Carboxylic Acid
Source: Int J Mol Sci. 2024 Jul 30;25(15):8356. doi: 10.3390/ijms25158356 (PMC11313329; doi:10.3390/ijms25158356)
Supplement: Supplementary file 1 [file ijms-25-08356-s001.zip › ijms-3108439-supplementary.pdf]

Supplementary Material

One stage pathway from hollongdione to C17-Alkyne and Vinyl Chloride followed Mannich bases and carboxylic acid

Zarema Galimova[1], Irina Smirnova[1,*], Alexander Lobov[1], Dmitriy Polovyanenko[2], Tatyana Rybalova[2], Oxana Kazakova[1]

[1] **Ufa Institute of Chemistry of the Ufa Federal Research Centre of the Russian Academy of Sciences, 71, pr. Oktyabrya, 450054 Ufa, Russian Federation; si8081@yandex.ru (I.S.); chemizara@gmail.com (Z.G.); lobovan@anrb.ru (A.L.); obf@anrb.ru (O.K.)**

[2] **N. N. Vorozhtsov Novosibirsk Institute of Organic Chemistry SB RAS, 630090 Novosibirsk, Russia; rybalova@nioch.nsc.ru (T.R.); dpolo@nioch.nsc.ru (D.P.)**

* **Correspondence: si8081@yandex.ru**

Abstract: A simple one-stage transformation of hollongdione, the first recorded example of the occurrence of a dammarane hexanor-triterpene in nature possessing antiviral and cytotoxic activity, into compounds with terminal alkyne and vinyl chloride fragments via the interaction with phosphorus halides is reported. The copper(I)-catalysed Mannich reaction of 3-oxo-22,23,24,25,26,27-hexanor-dammar-20(21)-in **3** led to a series of aminomethylated products, while 17-carboxylic acid was obtained by ozone oxidation of 3-oxo-22,23,24,25,26,27-hexanor-dammar-20-chloro-20(21)-en's **4**, the following direct amidation of the latter has been developed. The structures of all new molecules were established by spectroscopic studies that included 2D NMR correlation methods, the molecular structures of compounds **2** - **5** were determined by X-ray analysis.

Keywords: dammarane triterpenoids; hollongdione; alkyne; vinyl chloride; Mannich base; hollongdionoic acid

NMR data

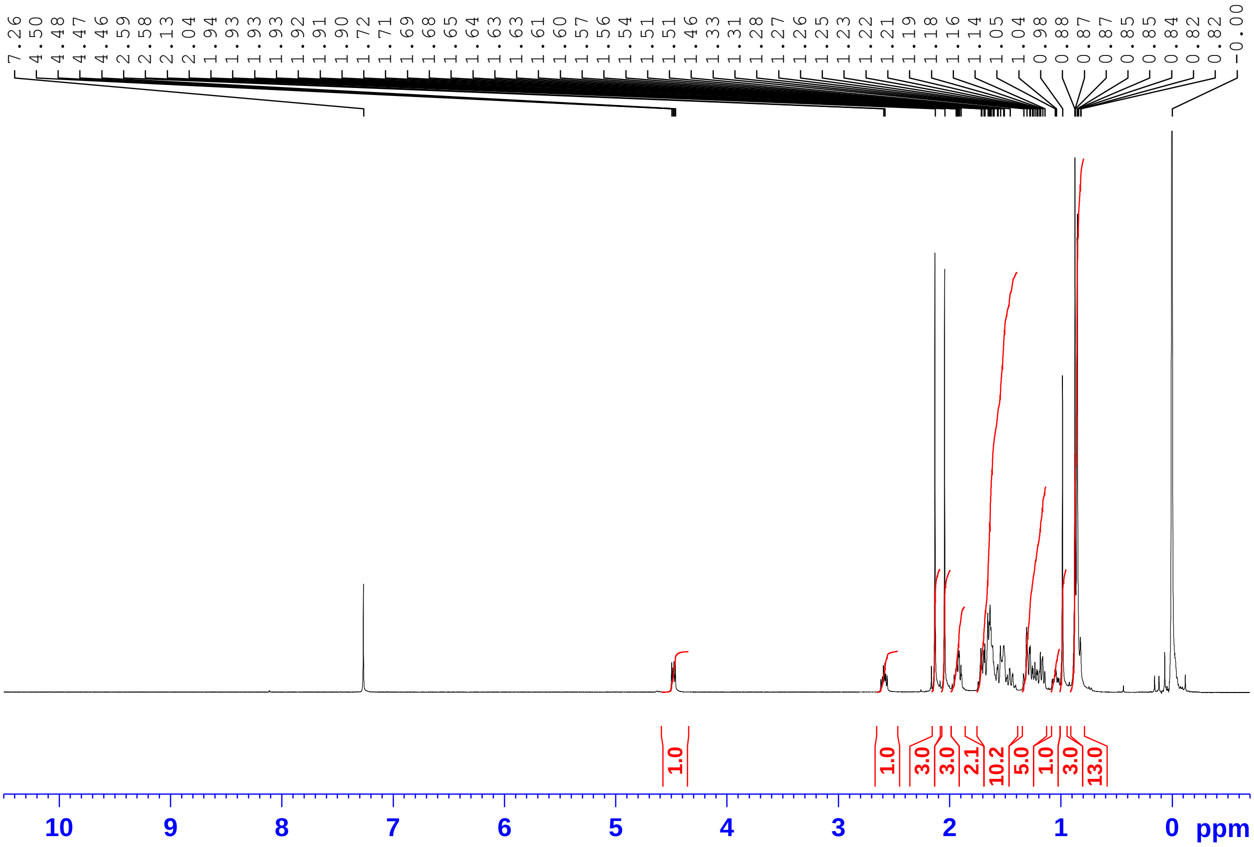

Figure S1. Complete ^{1}H NMR spectrum of compound **2** in $CDCl_3$, 500MHz.

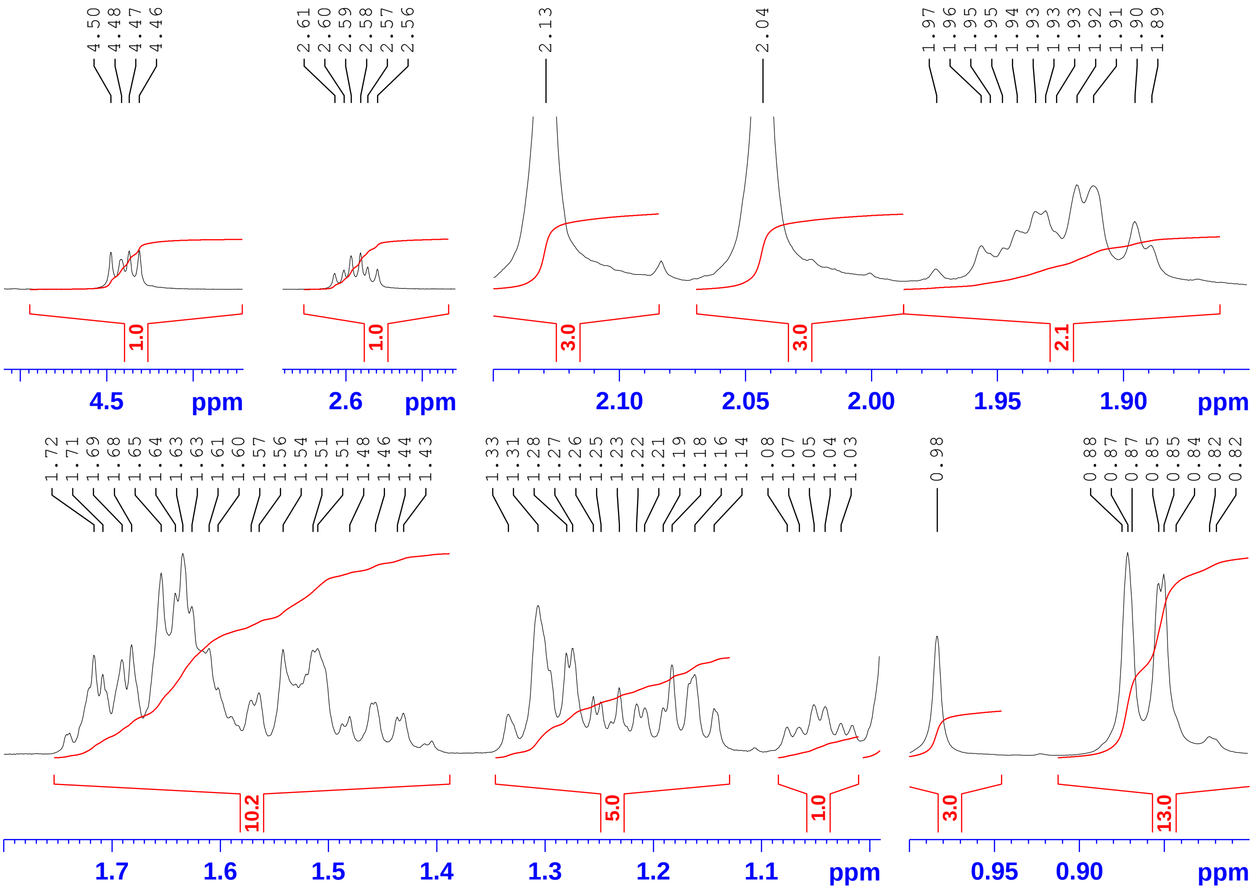

Figure S2. Expanded ^{1}H NMR spectrum of compound **2** in $CDCl_3$, 500MHz.

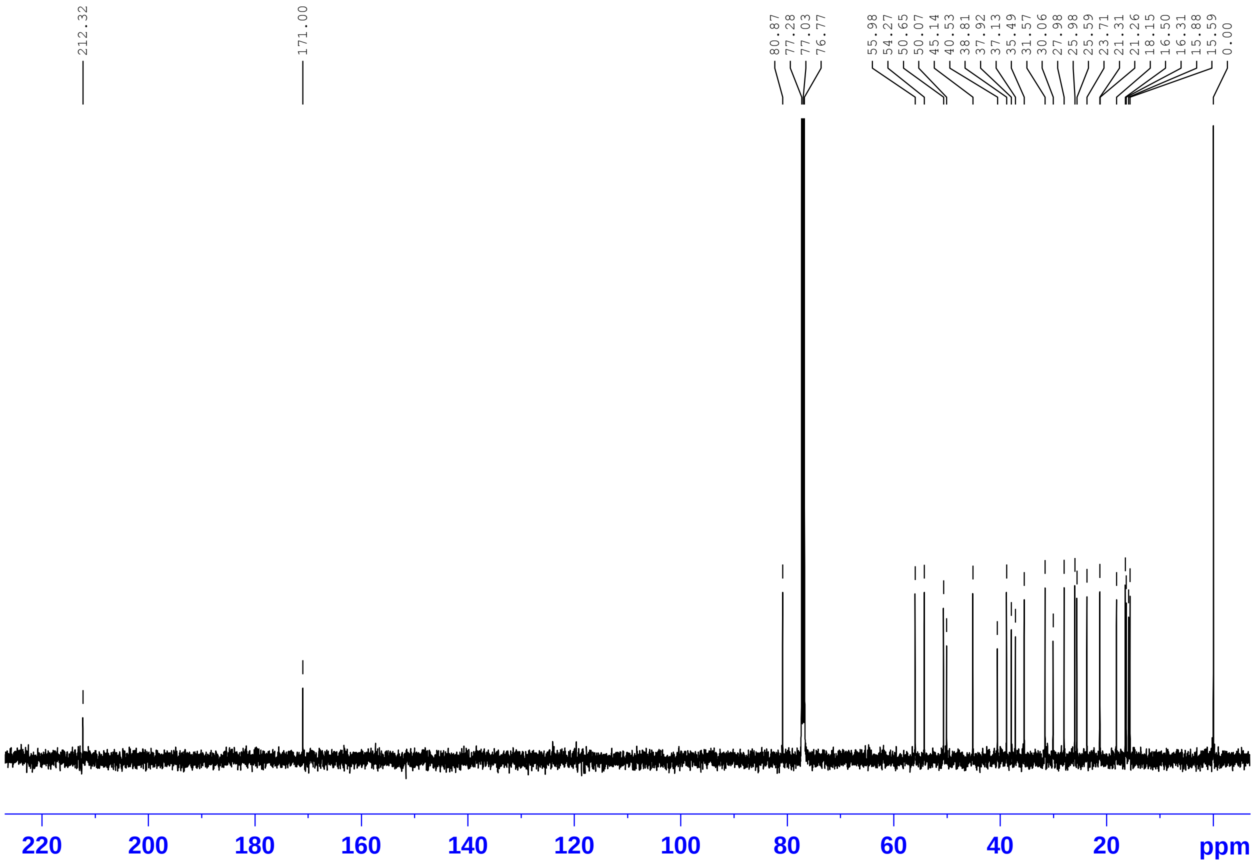

Figure S3. Complete $^{13}C\{^{1}H\}$ spectrum of compound **2** in $CDCl_3$, 125 MHz.

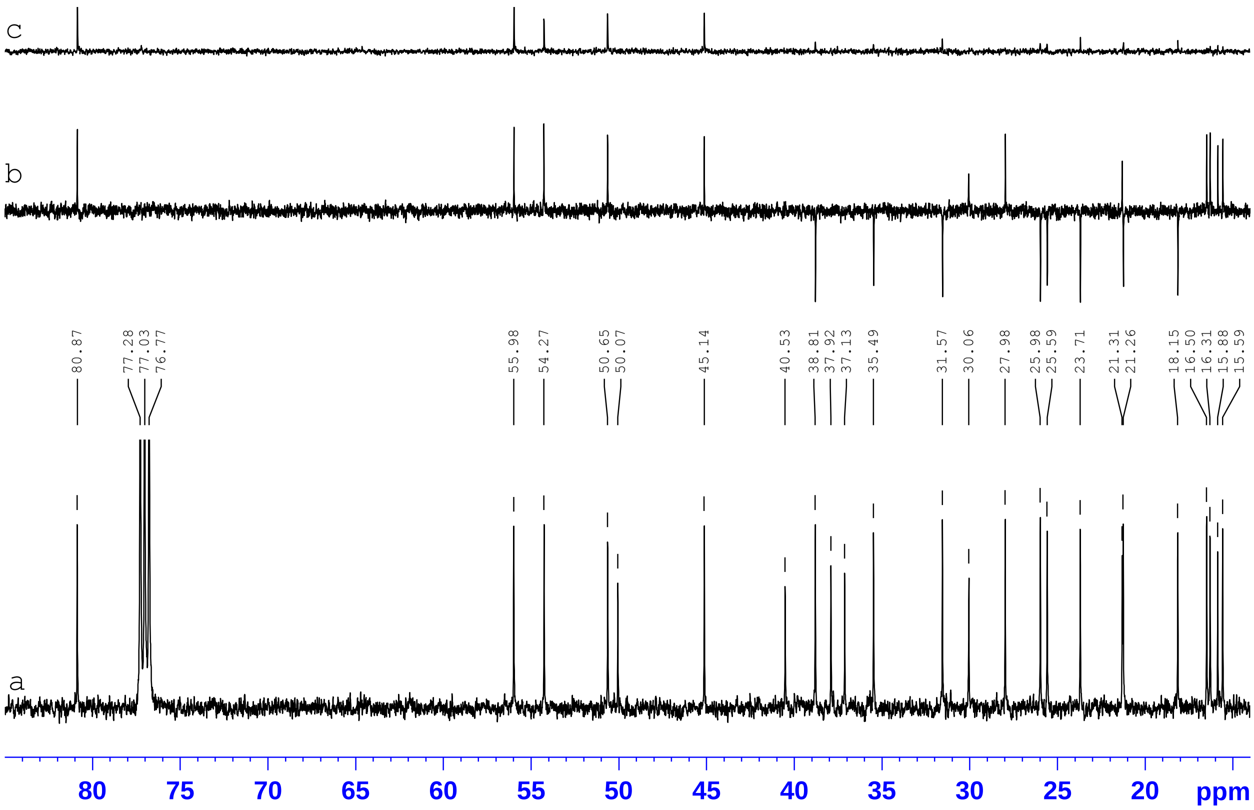

Figure S4. $^{13}C\{^{1}H\}$(a) NMR, DEPT-135 (b) and DEPT-90(c) spectra of compound **2** in $CDCl_3$, 125 MHz.

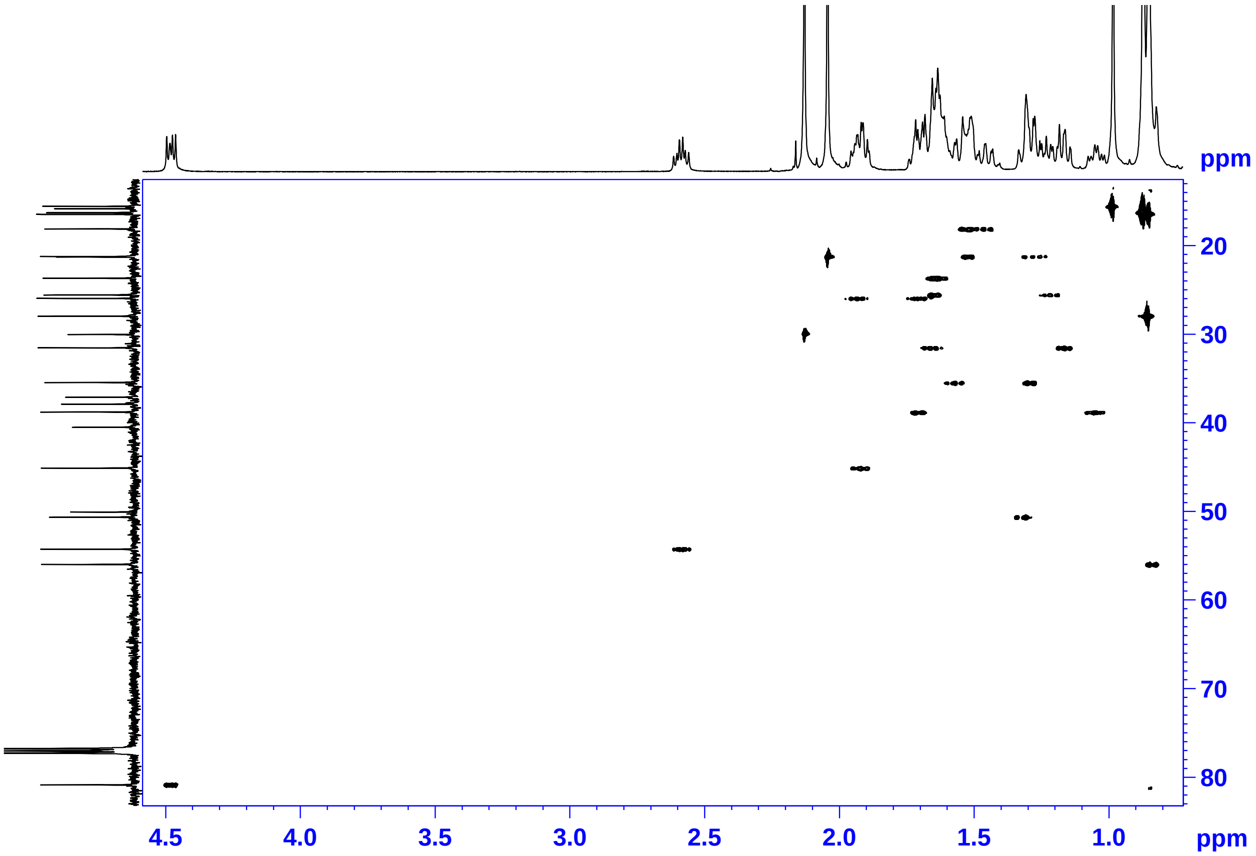

Figure S5. {¹H, ¹³C} HSQC spectrum of compound **2** in $CDCl_3$, 500 MHz.

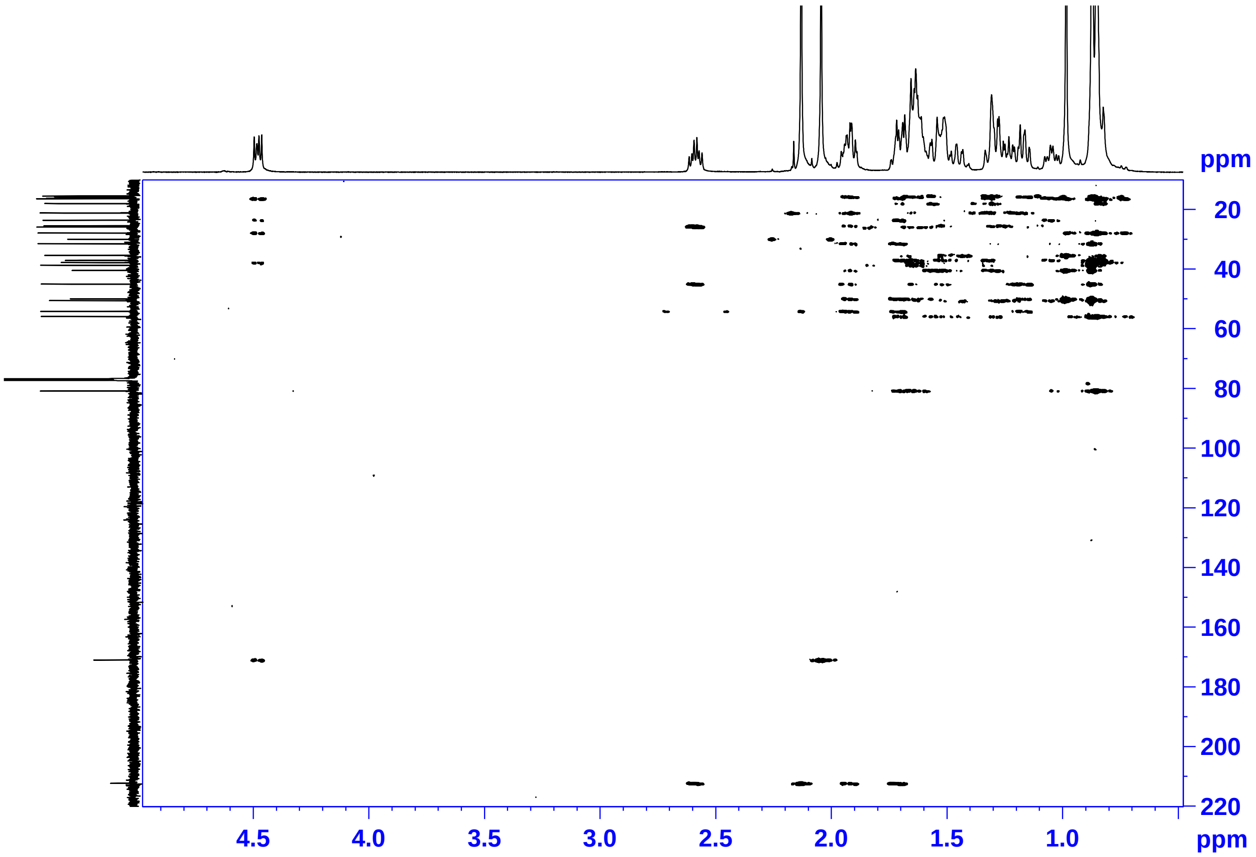

Figure S6. {¹H, ¹³C} HMBC spectrum of compound **2** in $CDCl_3$, 500 MHz.

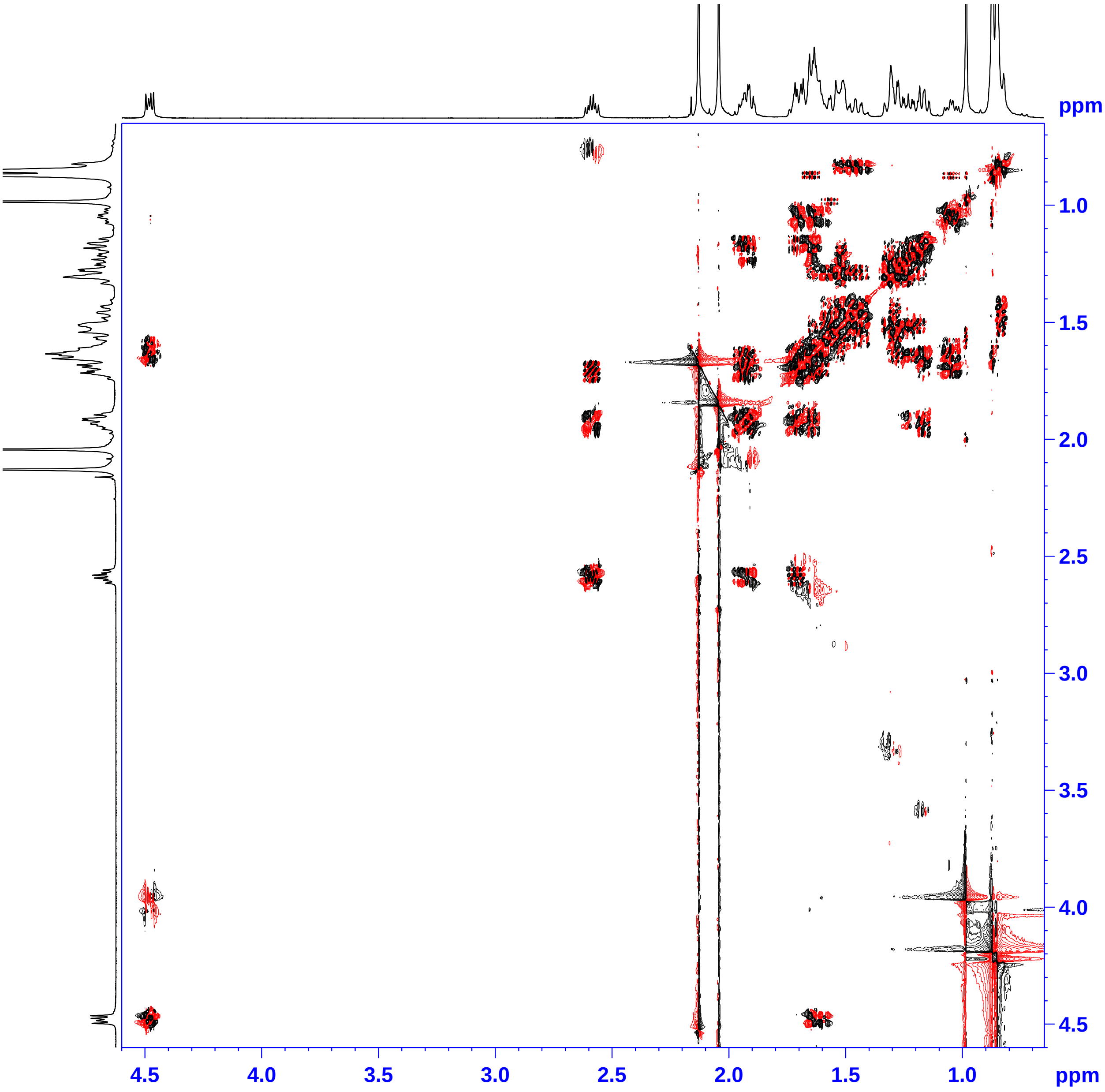

Figure S7. {¹H, ¹H} COSY-DQF spectrum of compound **2** in $CDCl_3$, 500 MHz.

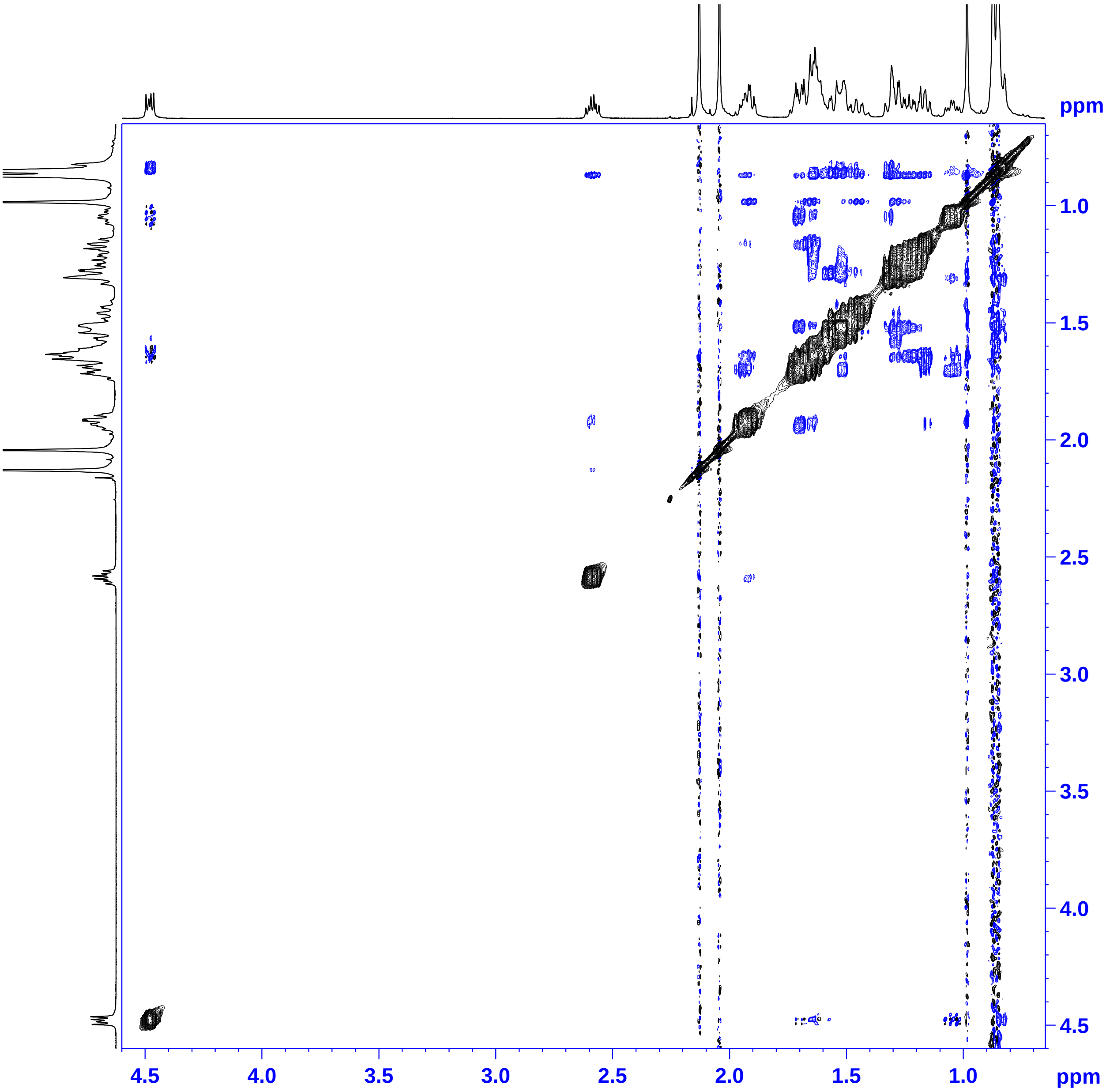

Figure S8. {^{1}H, ^{1}H} NOESY spectrum of compound **2** in $CDCl_3$, 500 MHz.

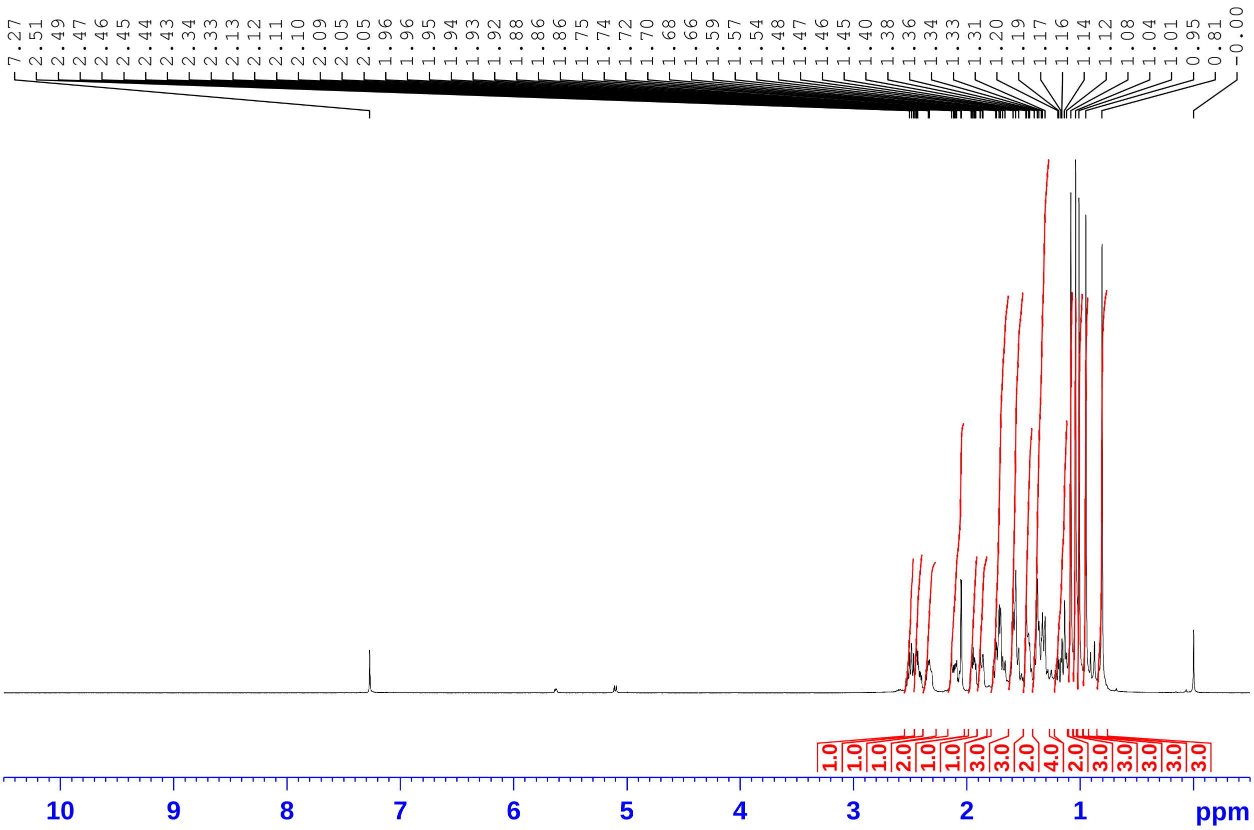

Figure S9. Complete ^{1}H NMR spectrum of compound **3** in $CDCl_3$, 500MHz.

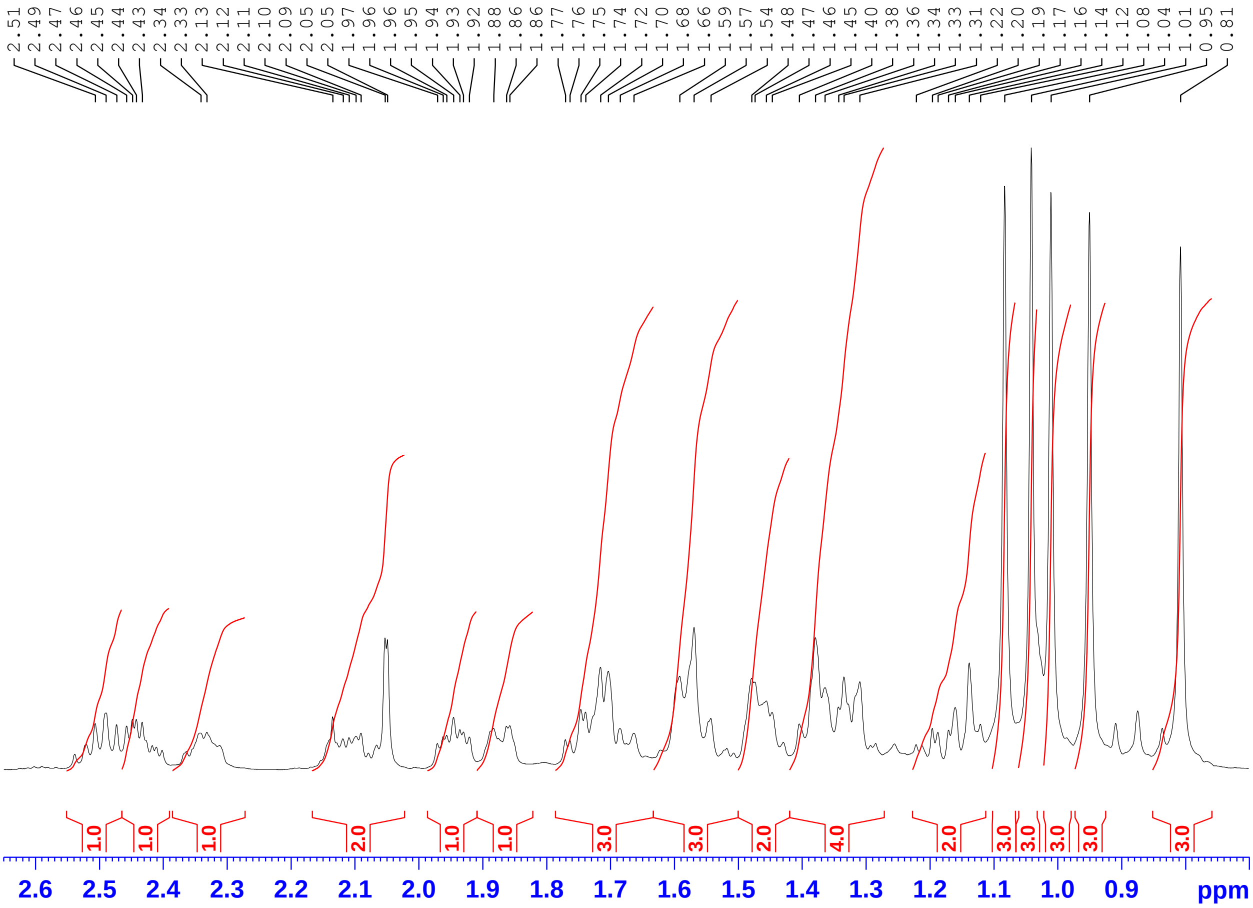

Figure S10. Expanded ^{1}H NMR spectrum of compound **3** in $CDCl_3$, 500MHz.

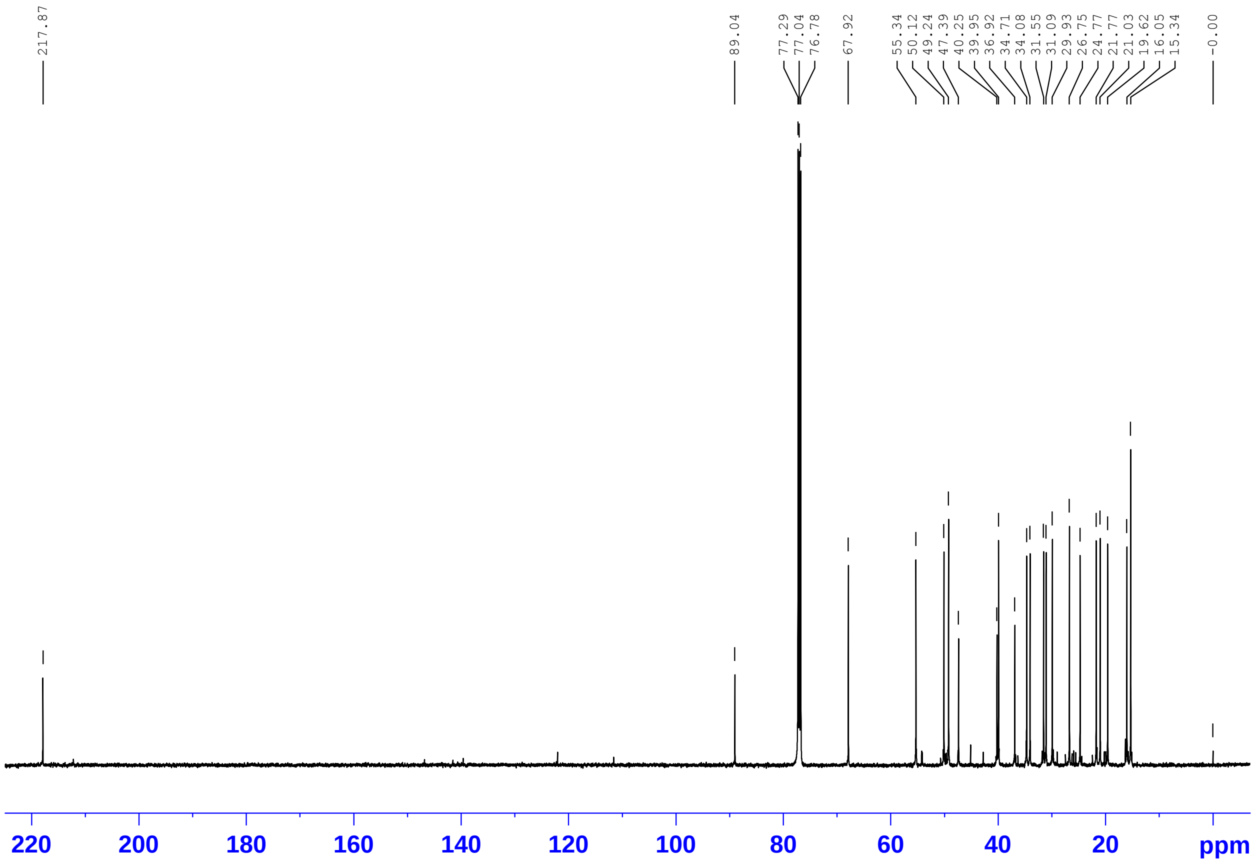

Figure S11. Complete ^{13}C{^{1}H} spectrum of compound **3** in $CDCl_3$, 125 MHz.

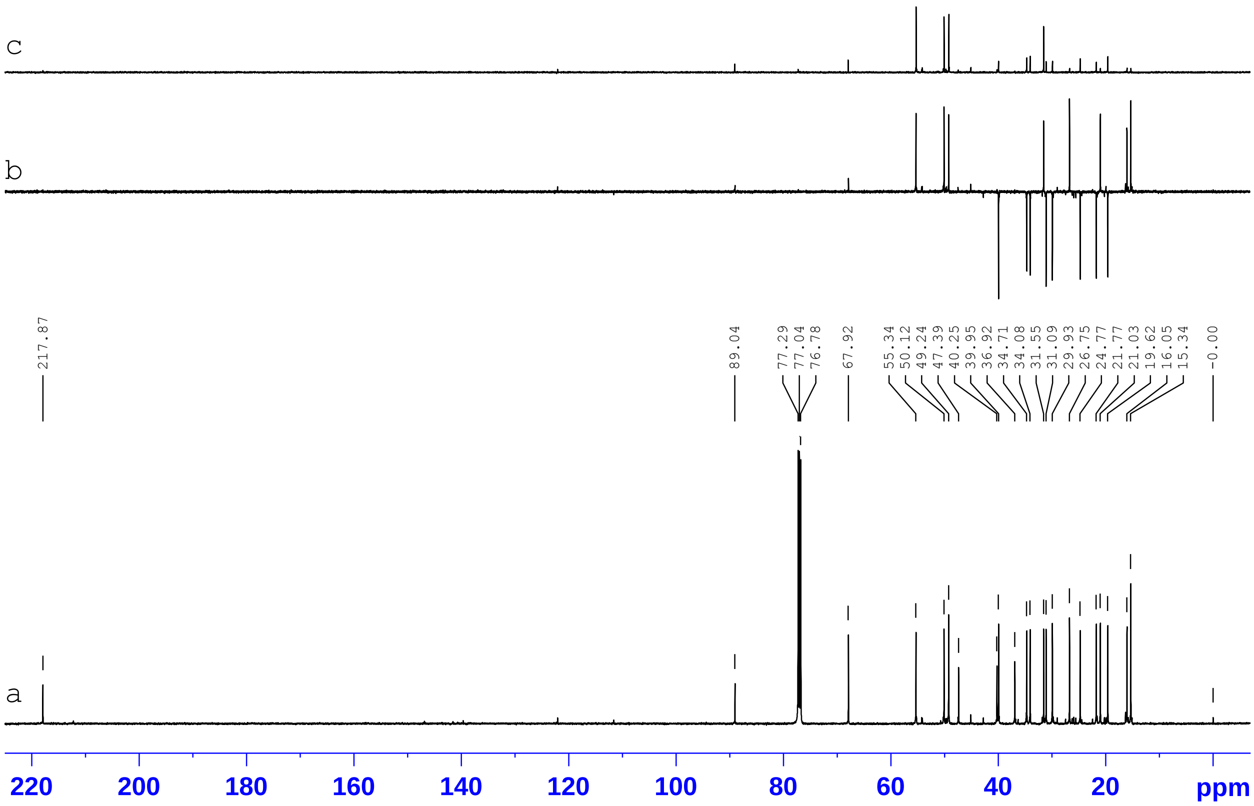

Figure S12. ^{13}C{^{1}H}(a) NMR, DEPT-135 (b) and DEPT-90(c) spectra of compound **3** in $CDCl_3$, 125 MHz.

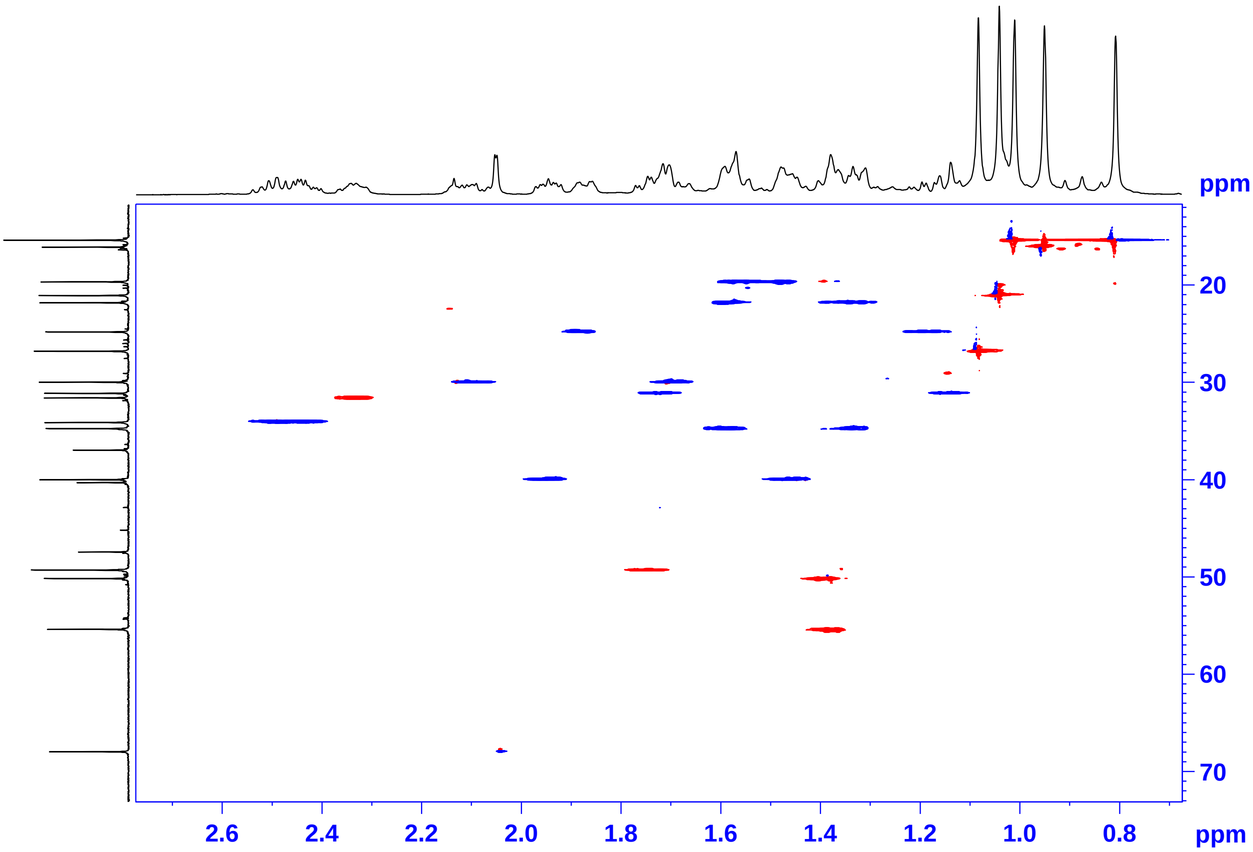

Figure S13. {^{1}H, ^{13}C} HSQCed spectrum of compound **3** in $CDCl_3$, 500 MHz.

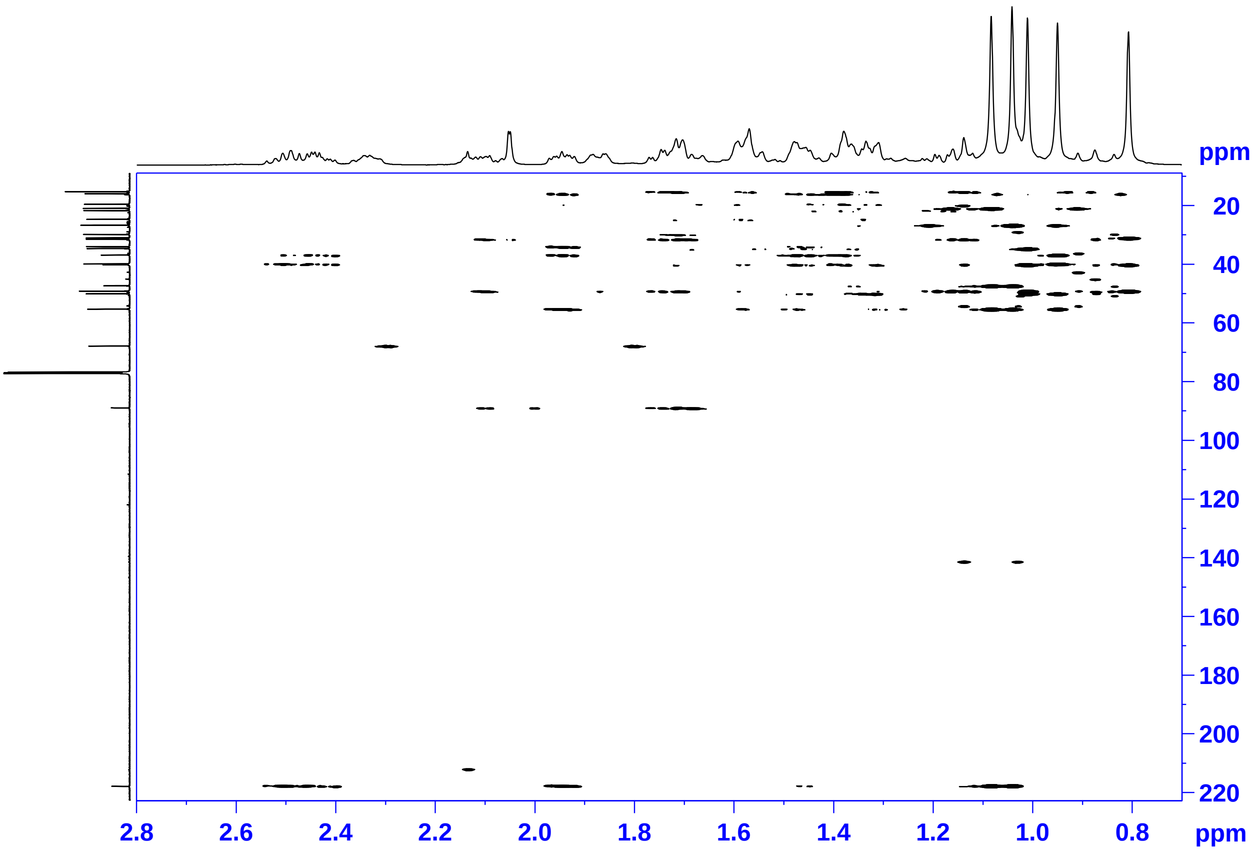

Figure S14. {^{1}H, ^{13}C} HMBC spectrum of compound **3** in $CDCl_3$, 500 MHz.

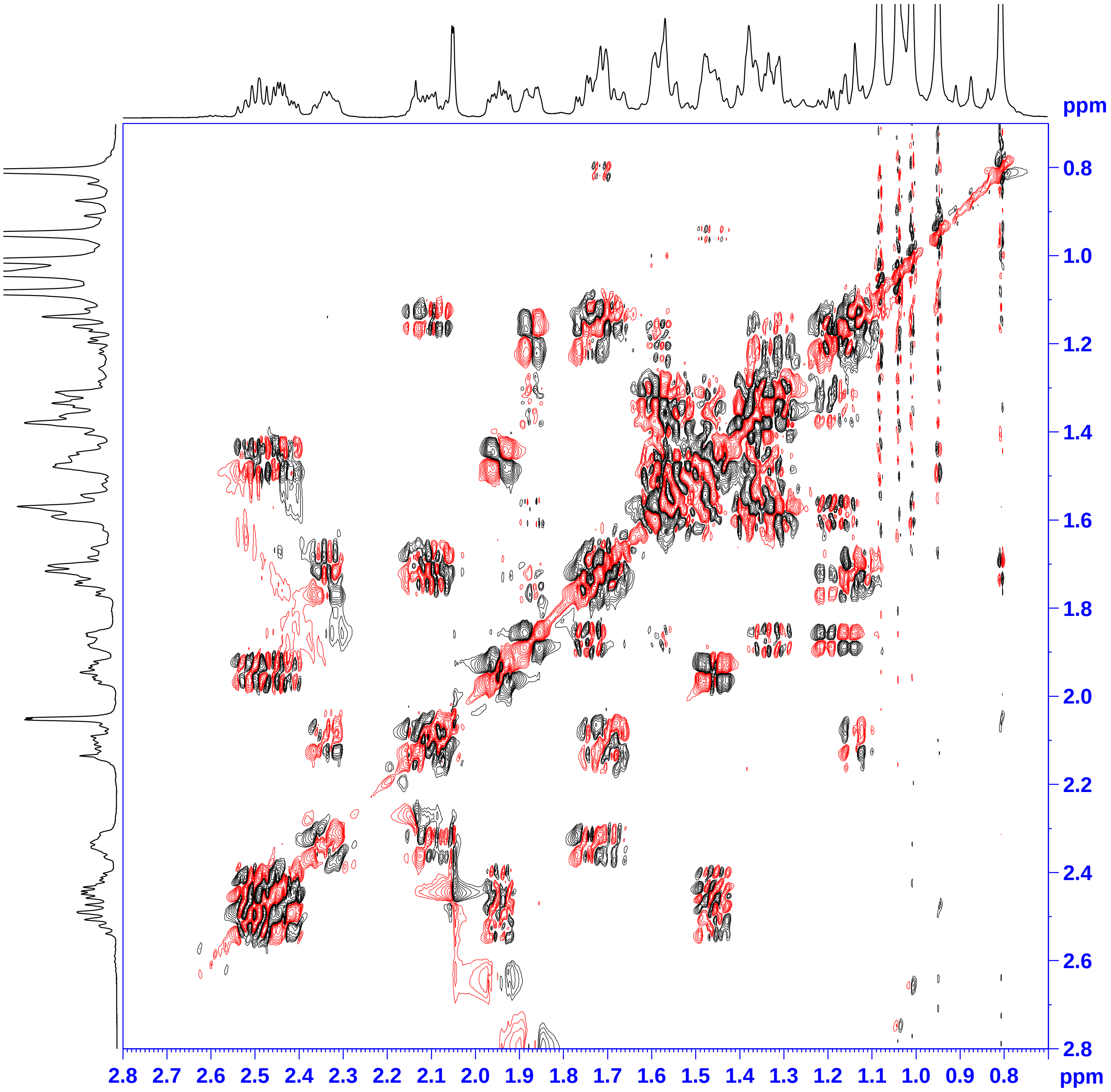

Figure S15. {^1H, ^1H} COSY-DQF spectrum of compound **3** in $CDCl_3$, 500 MHz.

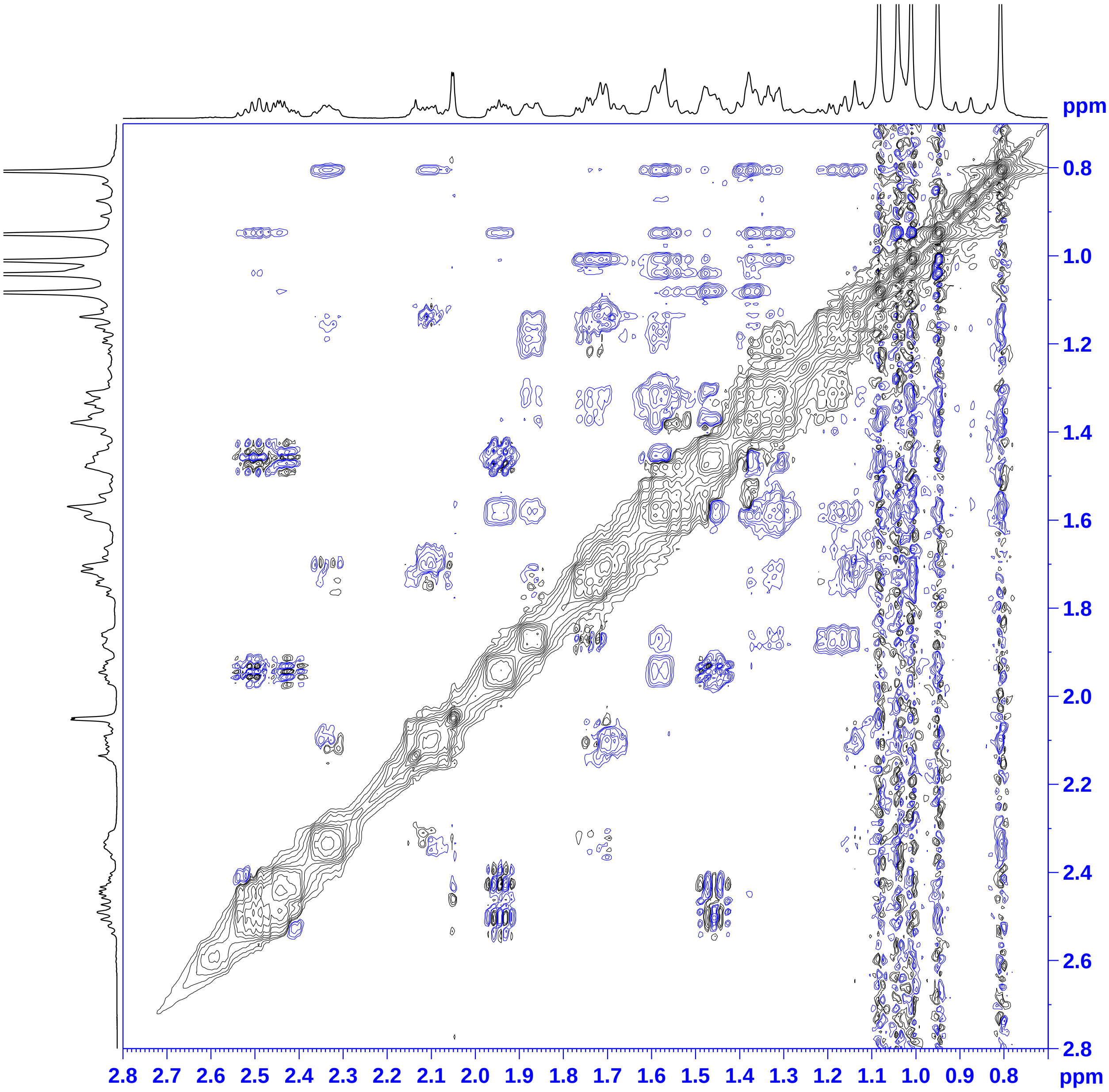

Figure S16. {^{1}H, ^{1}H} NOESY spectrum of compound **3** in $CDCl_3$, 500 MHz.

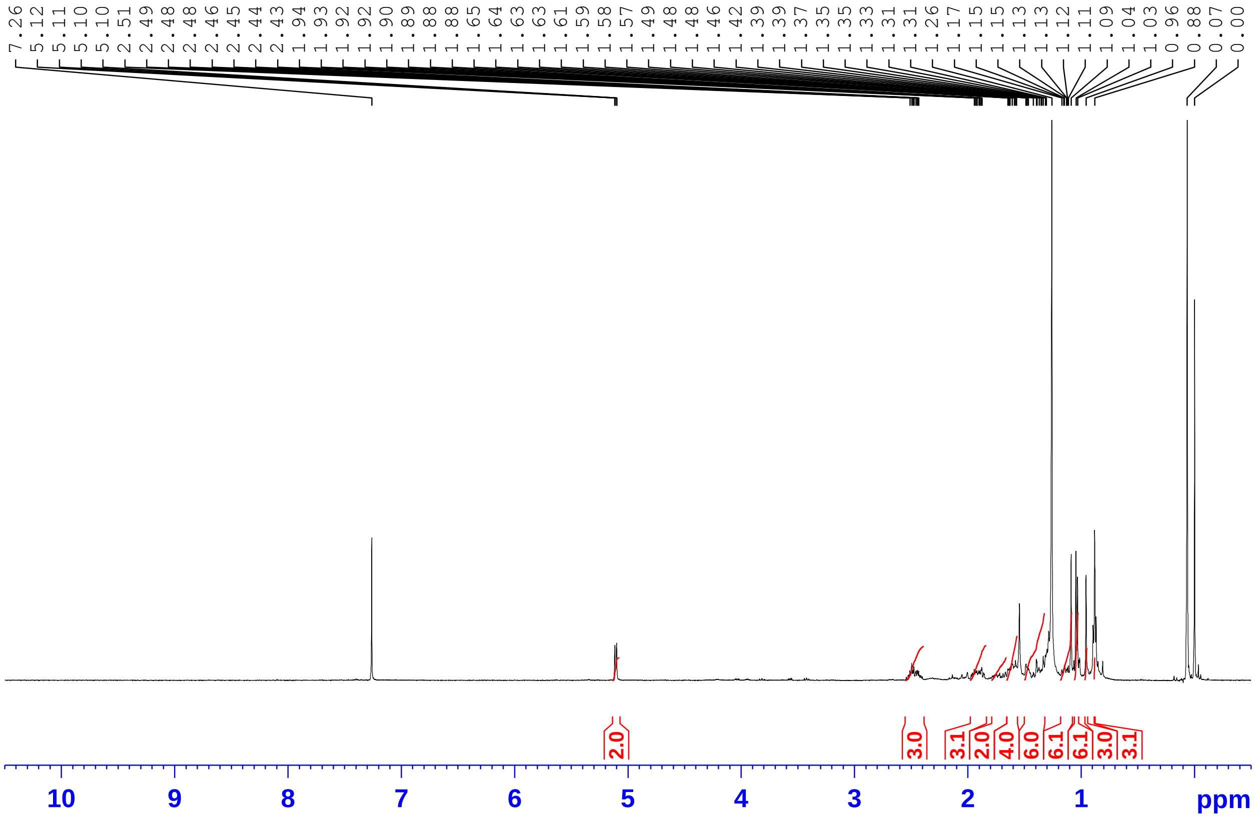

Figure S17. Complete ^{1}H NMR spectrum of compound **4** in $CDCl_3$, 500MHz.

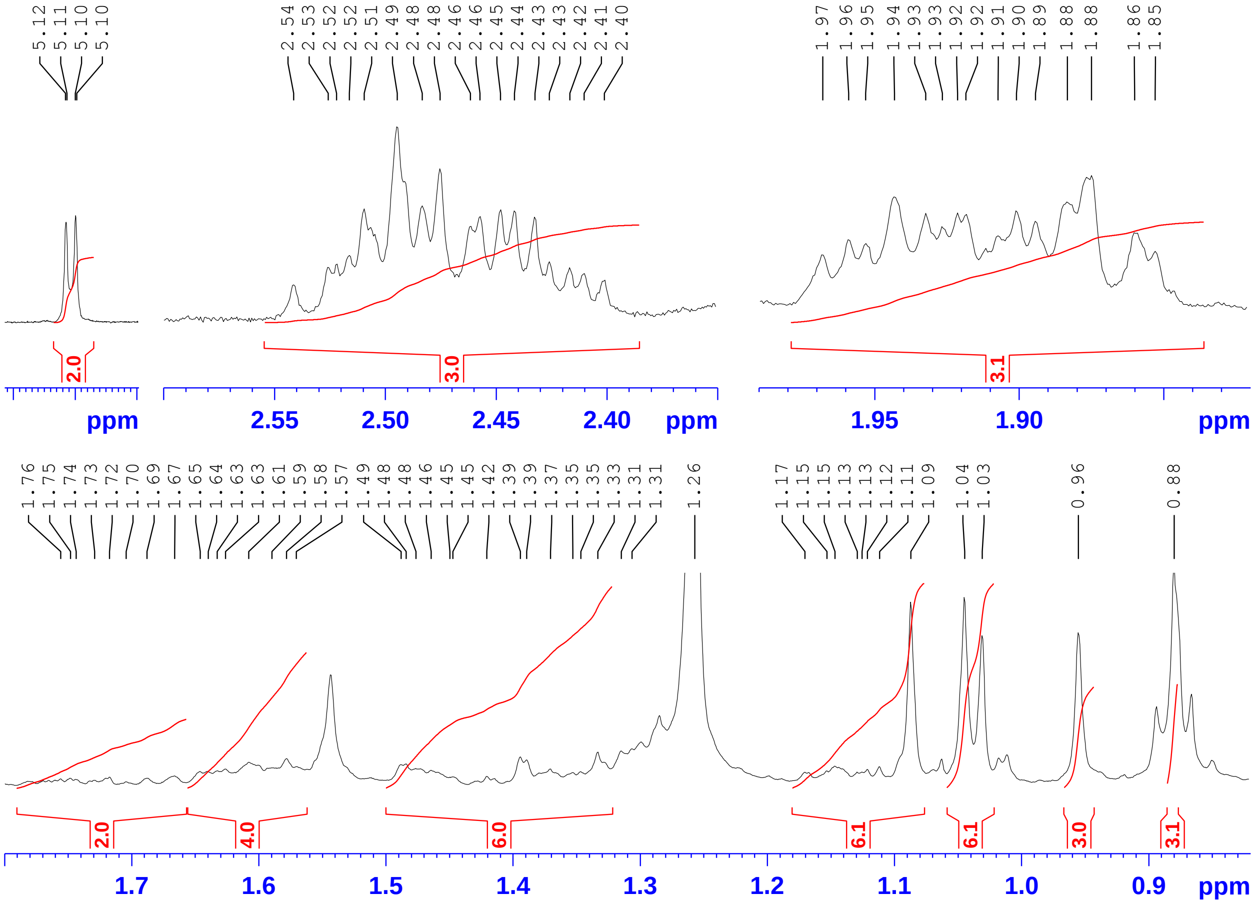

Figure S18. Expanded ^{1}H NMR spectrum of compound **4** in $CDCl_3$, 500MHz.

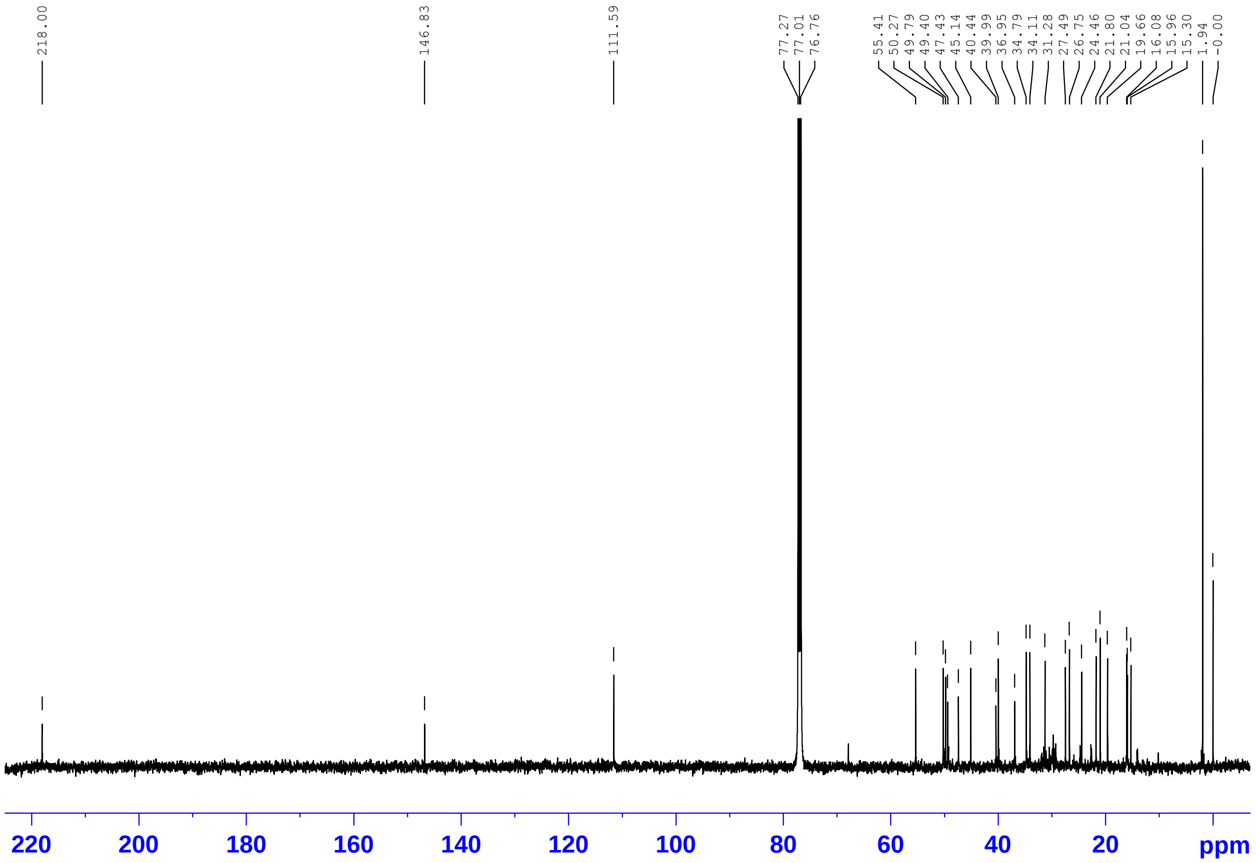

Figure S19. Complete $^{13}C\{^{1}H\}$ spectrum of compound **4** in $CDCl_3$, 125 MHz.

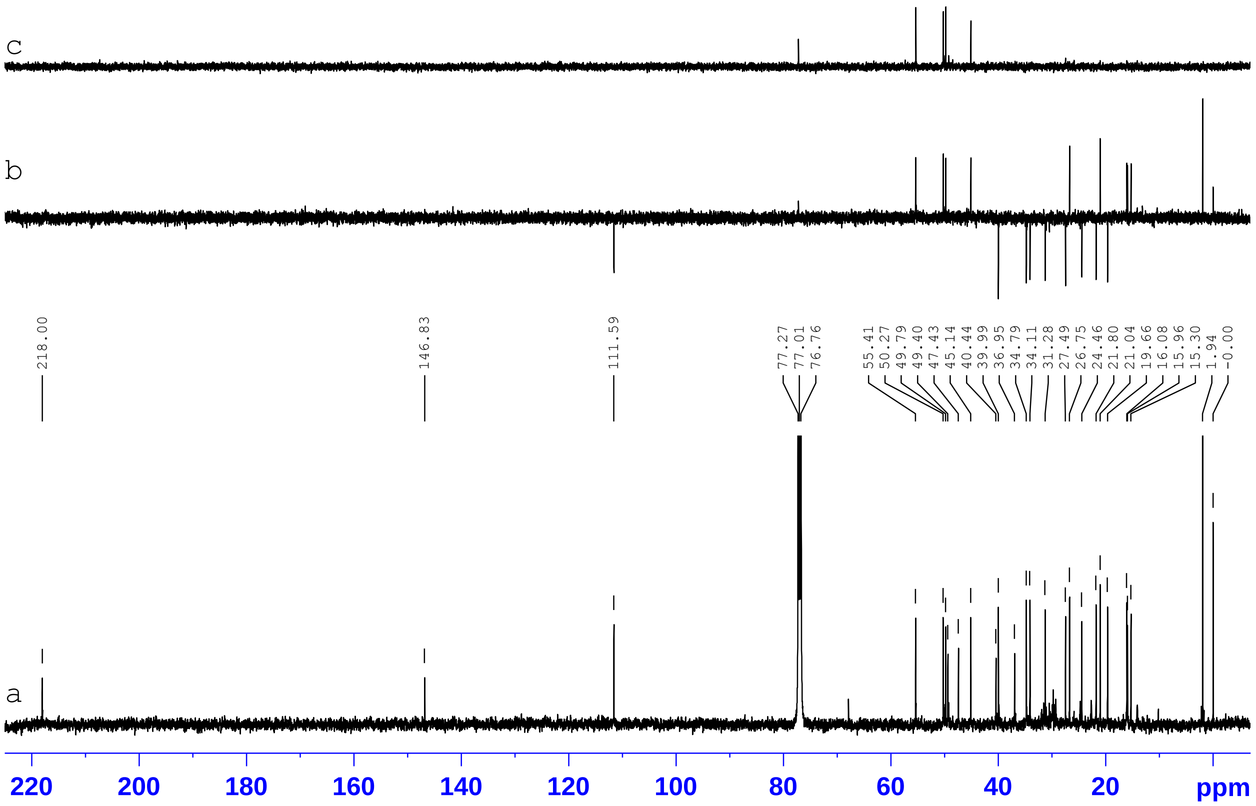

Figure S20. $^{13}C\{^{1}H\}$(a) NMR, DEPT-135 (b) and DEPT-90(c) spectra of compound **4** in $CDCl_3$, 125 MHz.

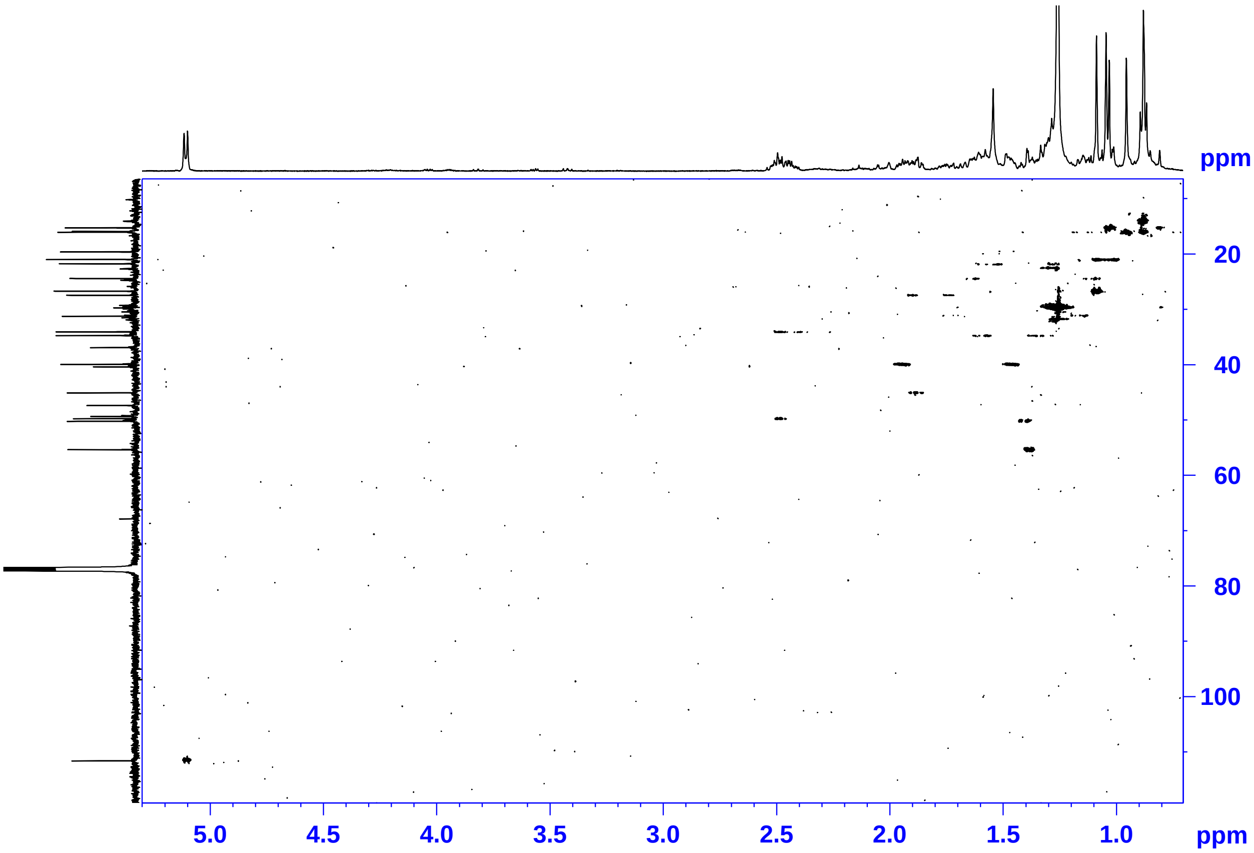

Figure S21. {¹H, ¹³C} HSQC spectrum of compound **4** in $CDCl_3$, 500 MHz.

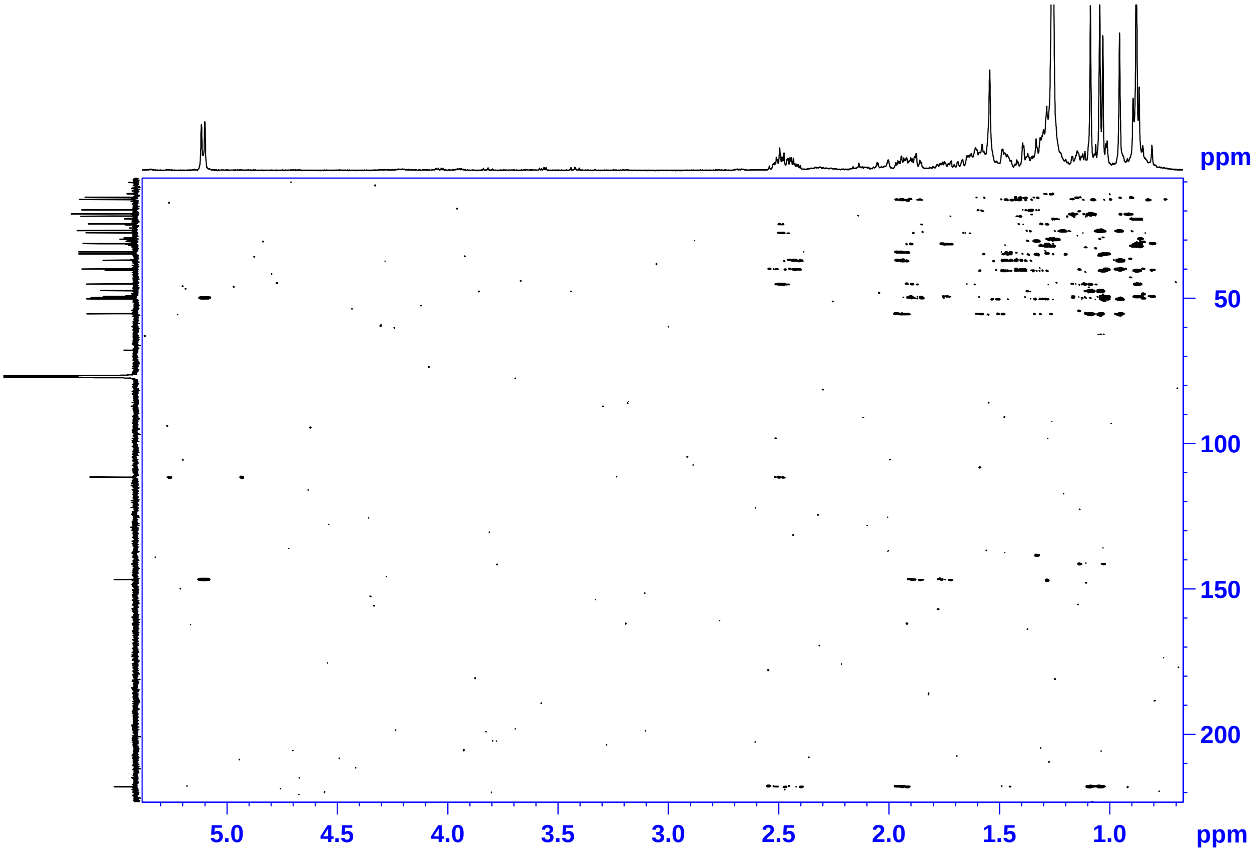

Figure S22. {¹H, ¹³C} HMBC spectrum of compound **4** in $CDCl_3$, 500 MHz.

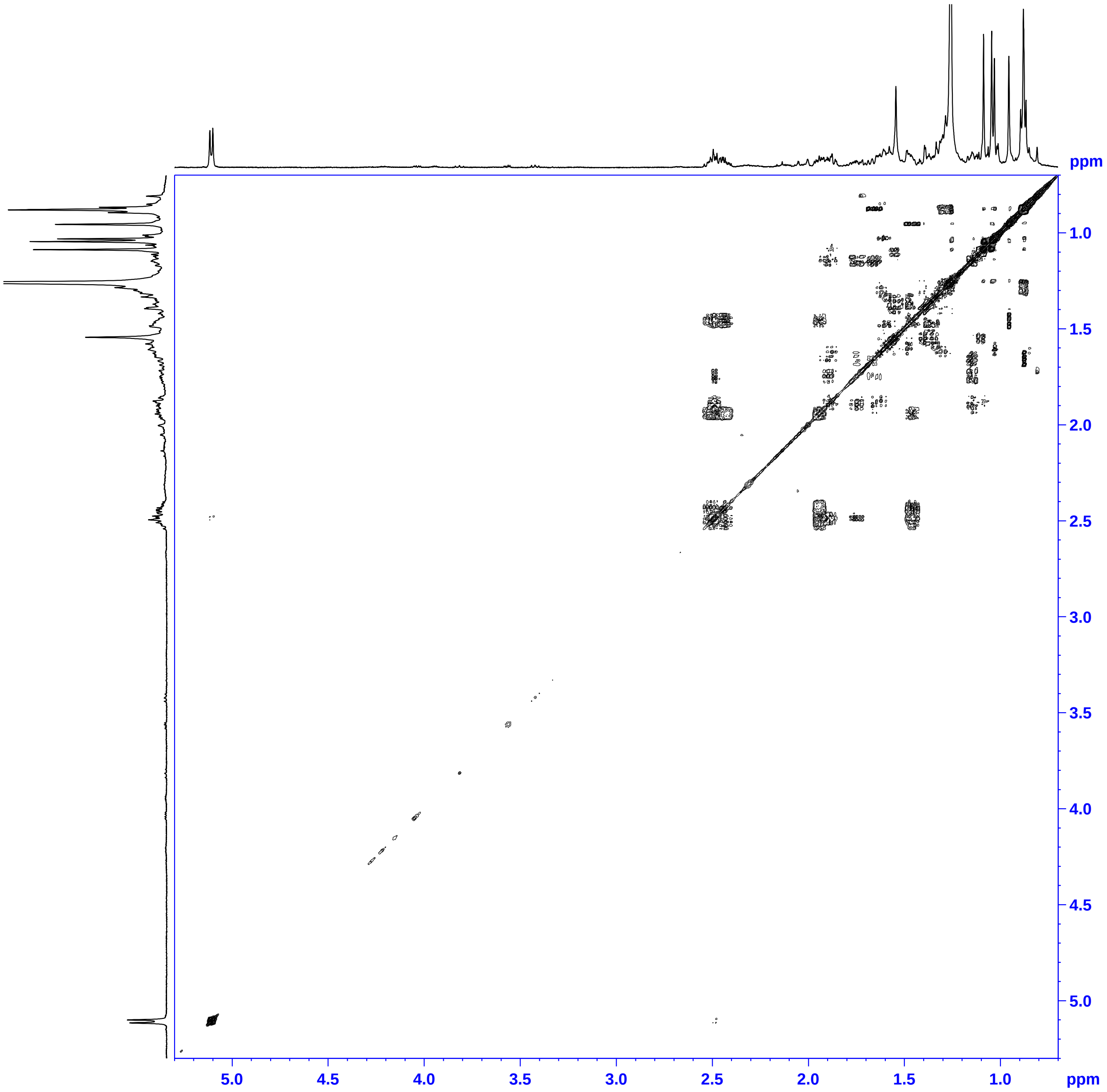

Figure S23. {^{1}H, ^{1}H} COSY spectrum of compound **4** in $CDCl_3$, 500 MHz.

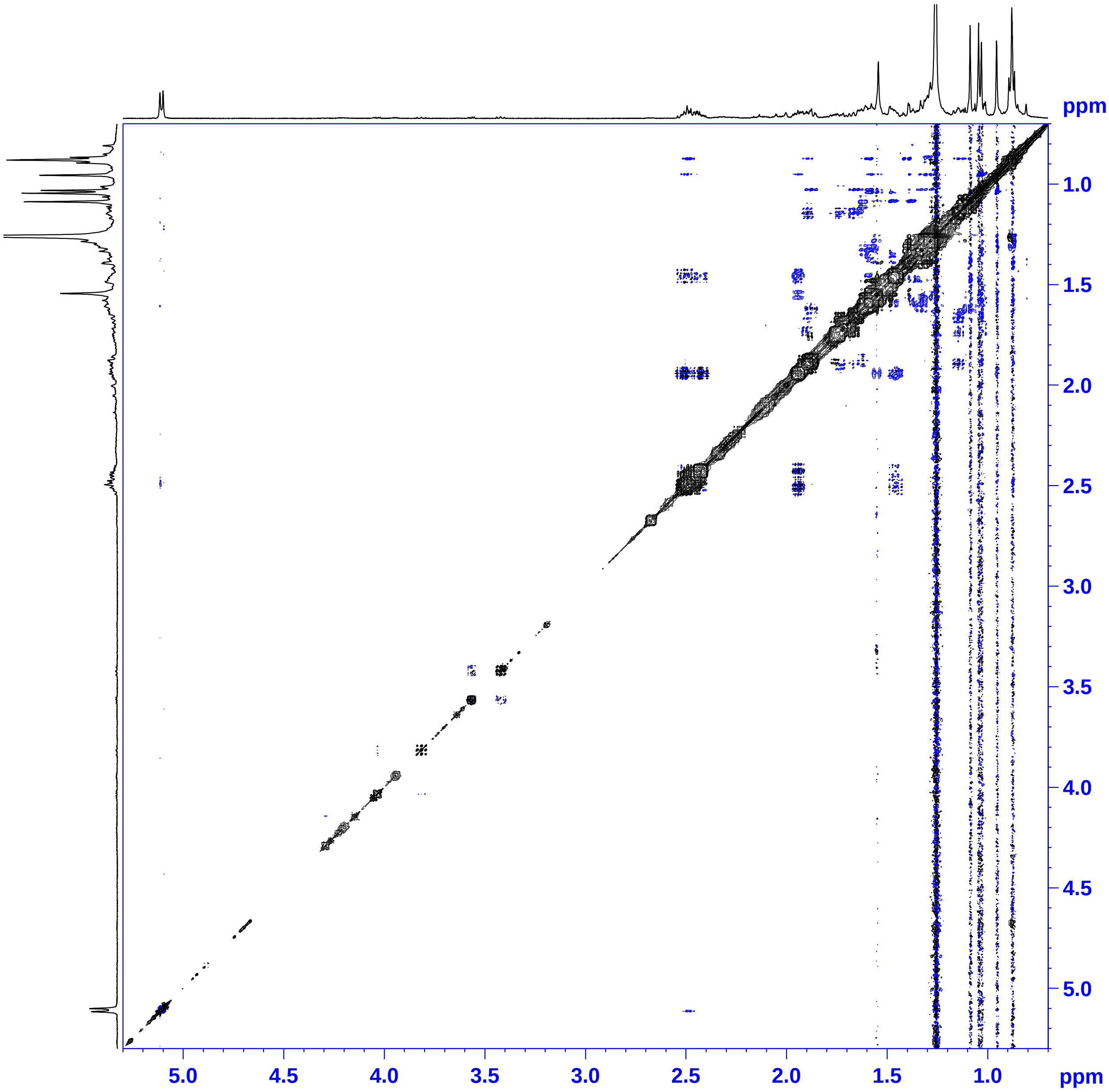

Figure S24. {^{1}H, ^{1}H} NOESY spectrum of compound **4** in $CDCl_3$, 500 MHz.

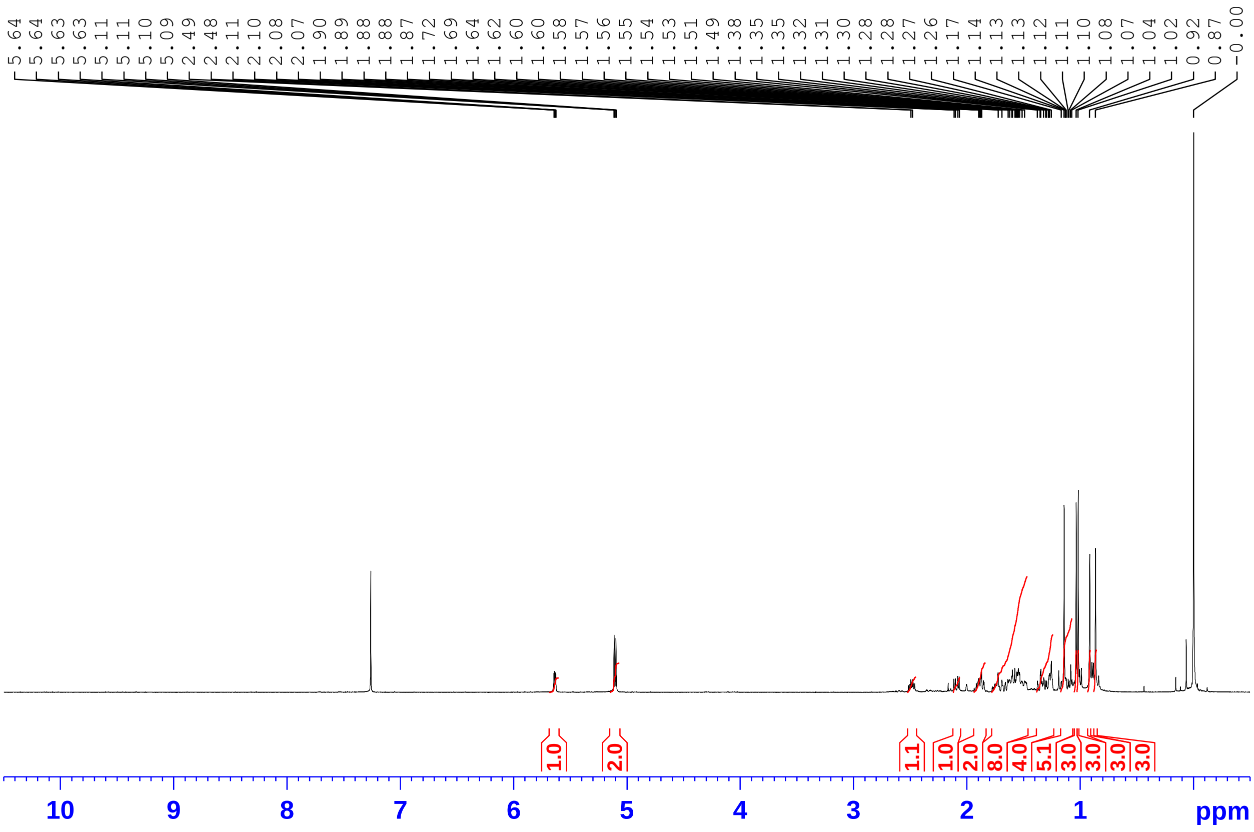

Figure S25. Complete ^{1}H NMR spectrum of compound **5** in $CDCl_3$, 500MHz.

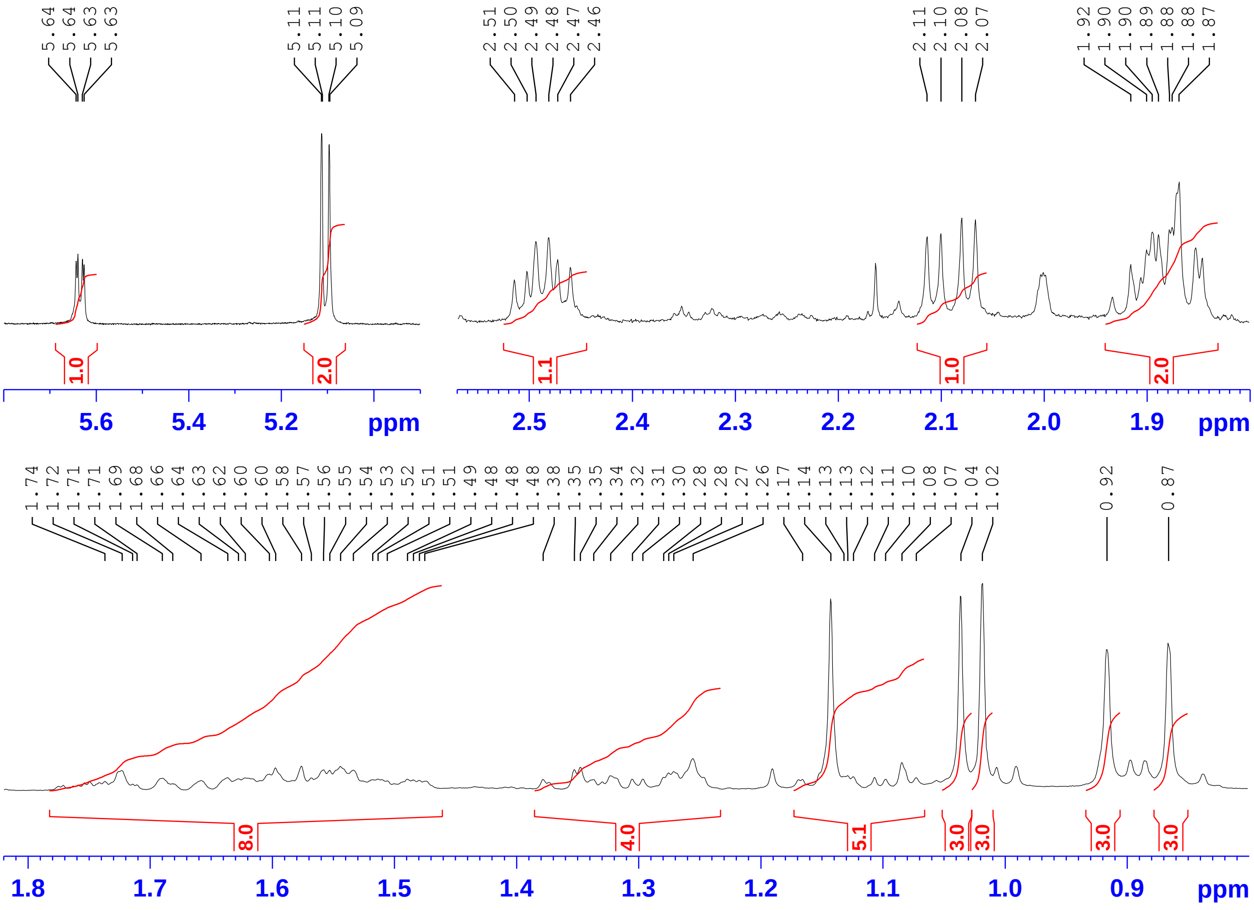

Figure S26. Expanded ^{1}H NMR spectrum of compound **5** in $CDCl_3$, 500MHz.

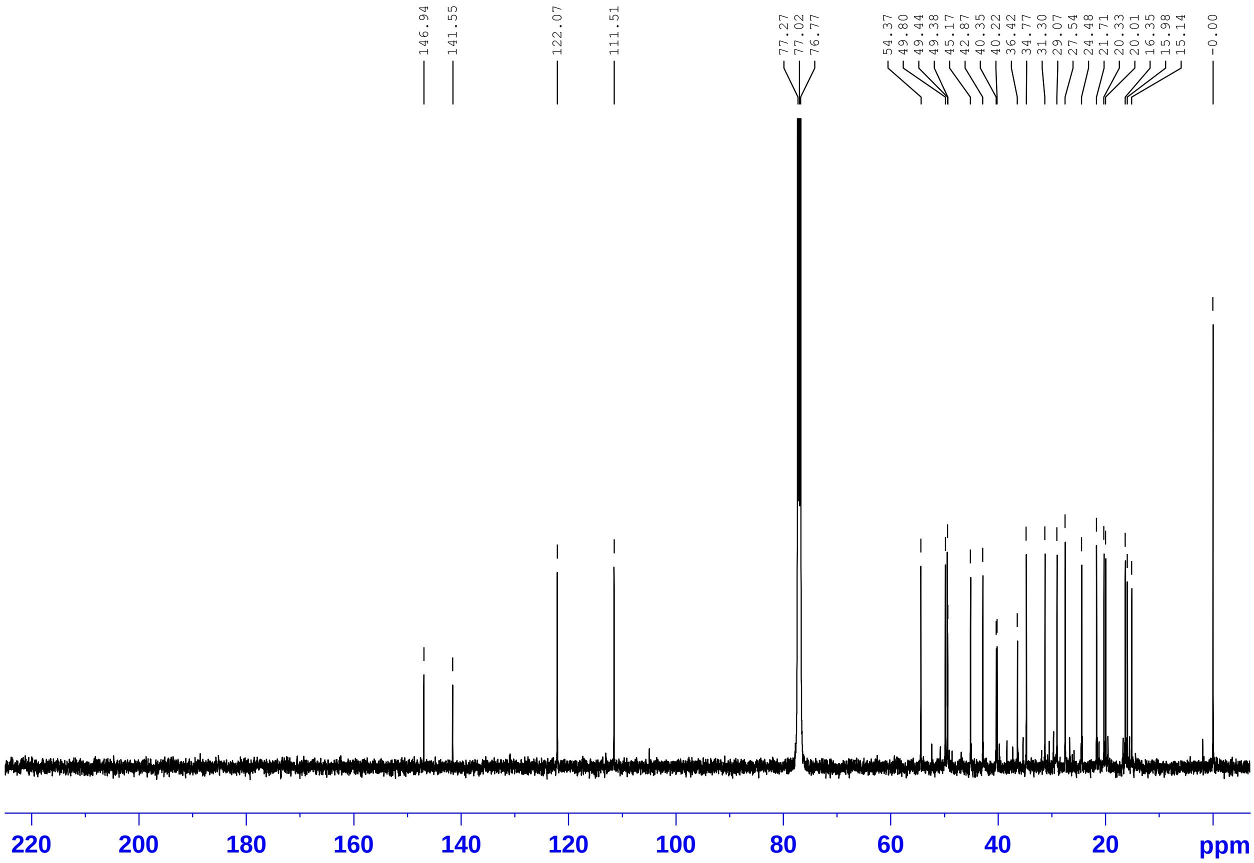

Figure S27. Complete $^{13}C\{^{1}H\}$ spectrum of compound **5** in $CDCl_3$, 125 MHz.

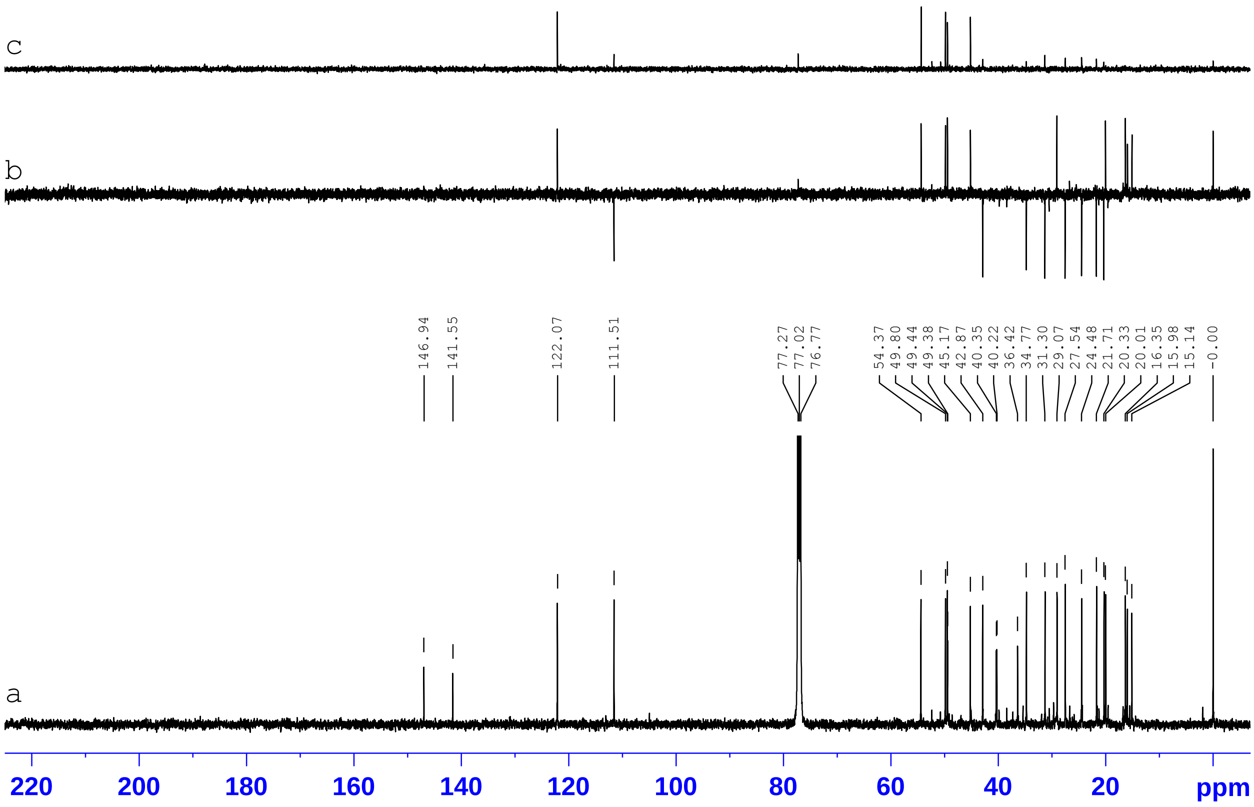

Figure S28. $^{13}C\{^{1}H\}$ and DEPT-135, DEPT-90 spectra of compound **5** in $CDCl_3$, 125 MHz.

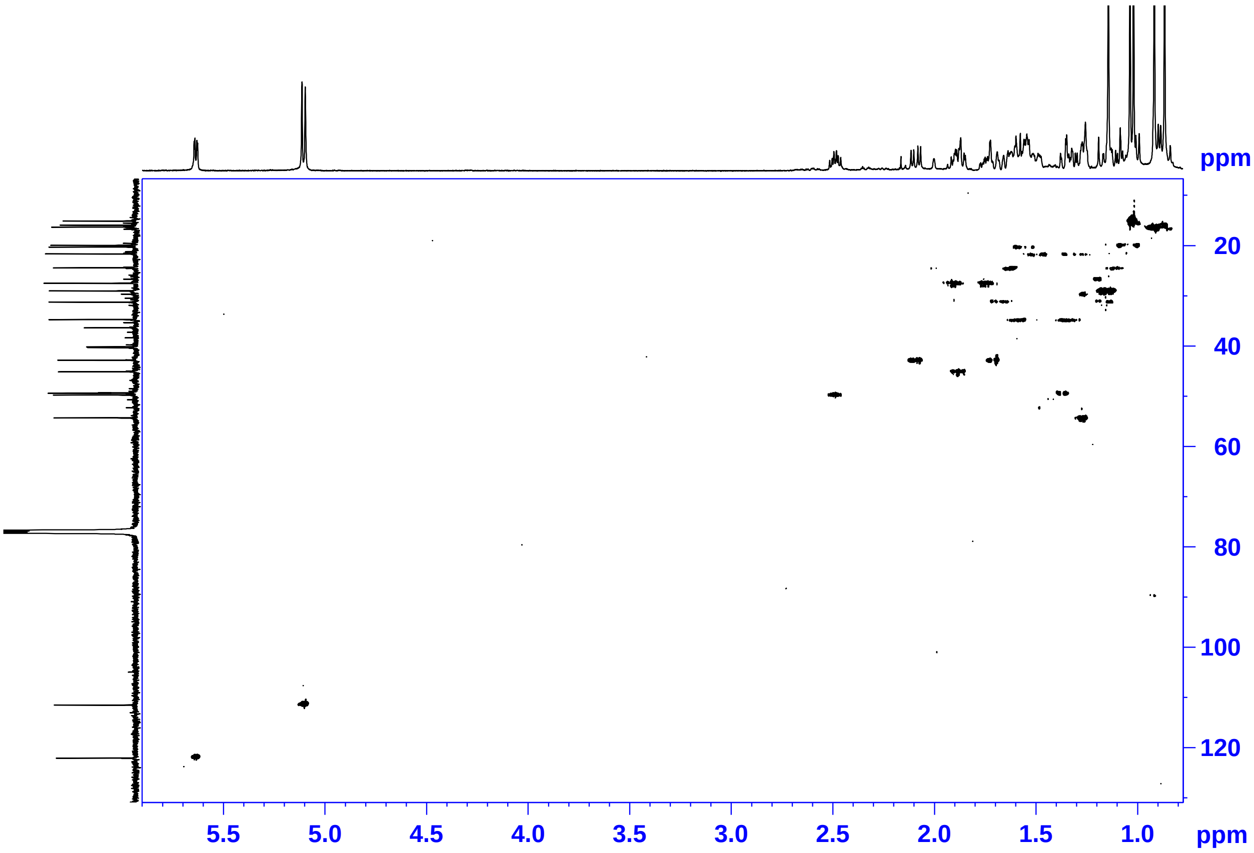

Figure S29. {^{1}H, ^{13}C} HSQC spectrum of compound **5** in $CDCl_3$, 500 MHz.

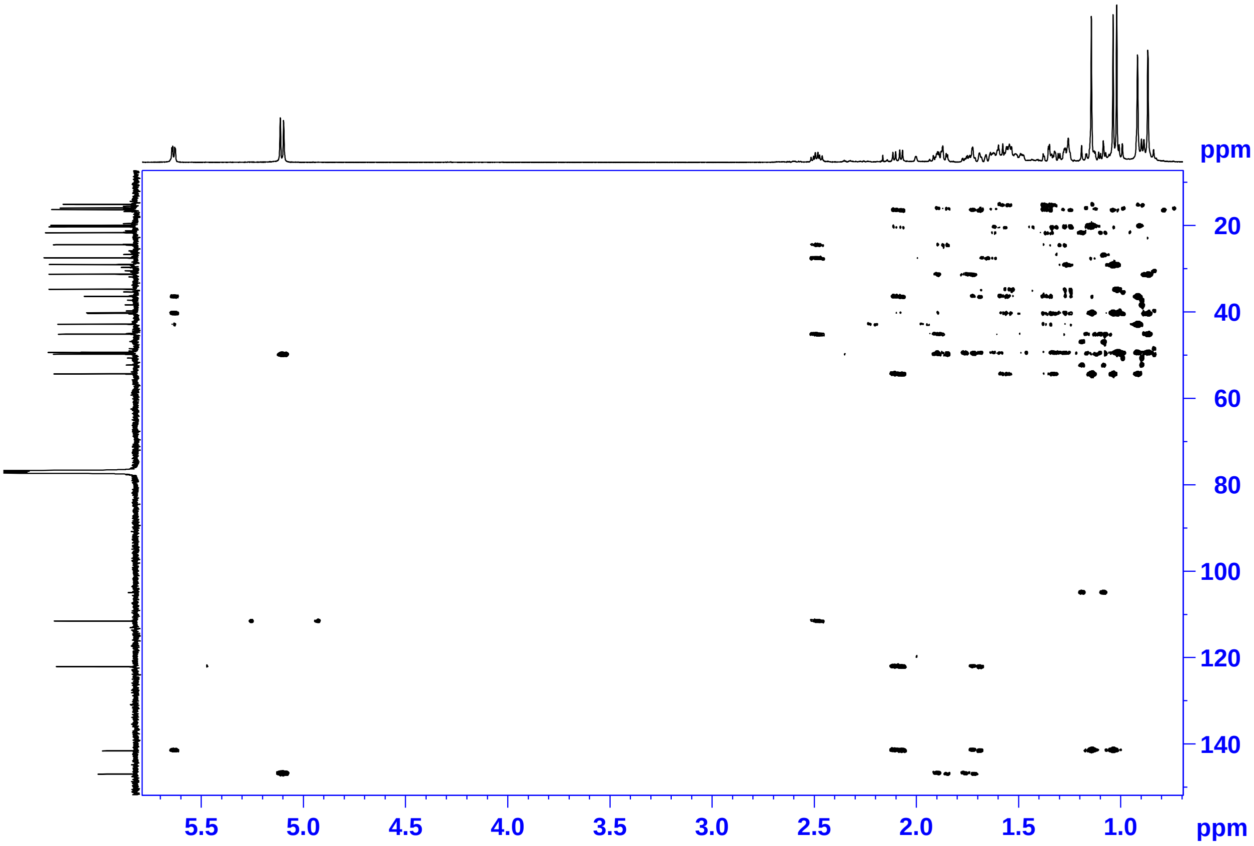

Figure S30. {^{1}H, ^{13}C} HMBC spectrum of compound **5** in $CDCl_3$, 500 MHz.

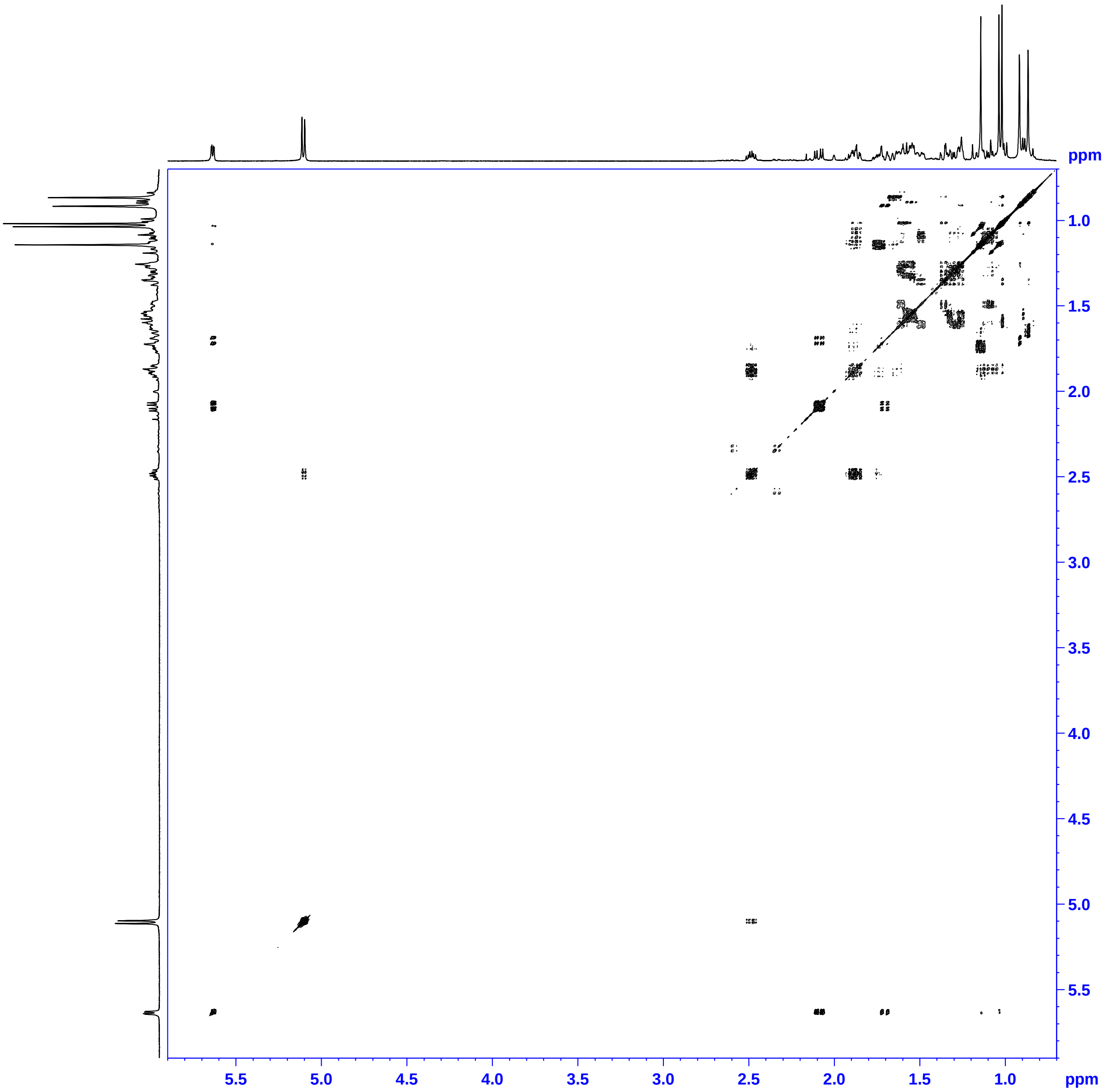

Figure S31. {^{1}H, ^{1}H} COSY spectrum of compound **5** in $CDCl_3$, 500 MHz.

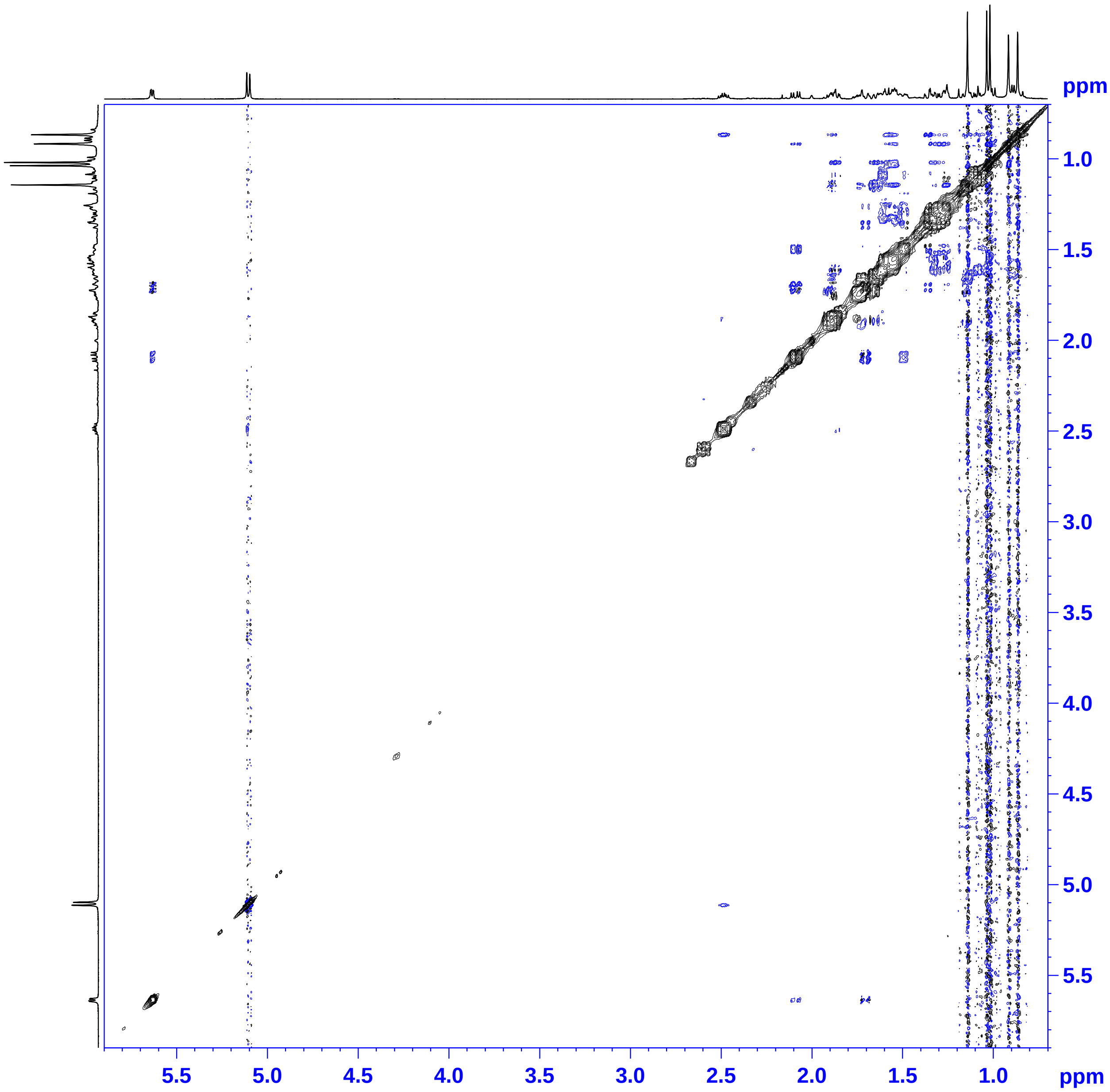

Figure S32. {^{1}H, ^{1}H} NOESY spectrum of compound **5** in $CDCl_3$, 500 MHz.

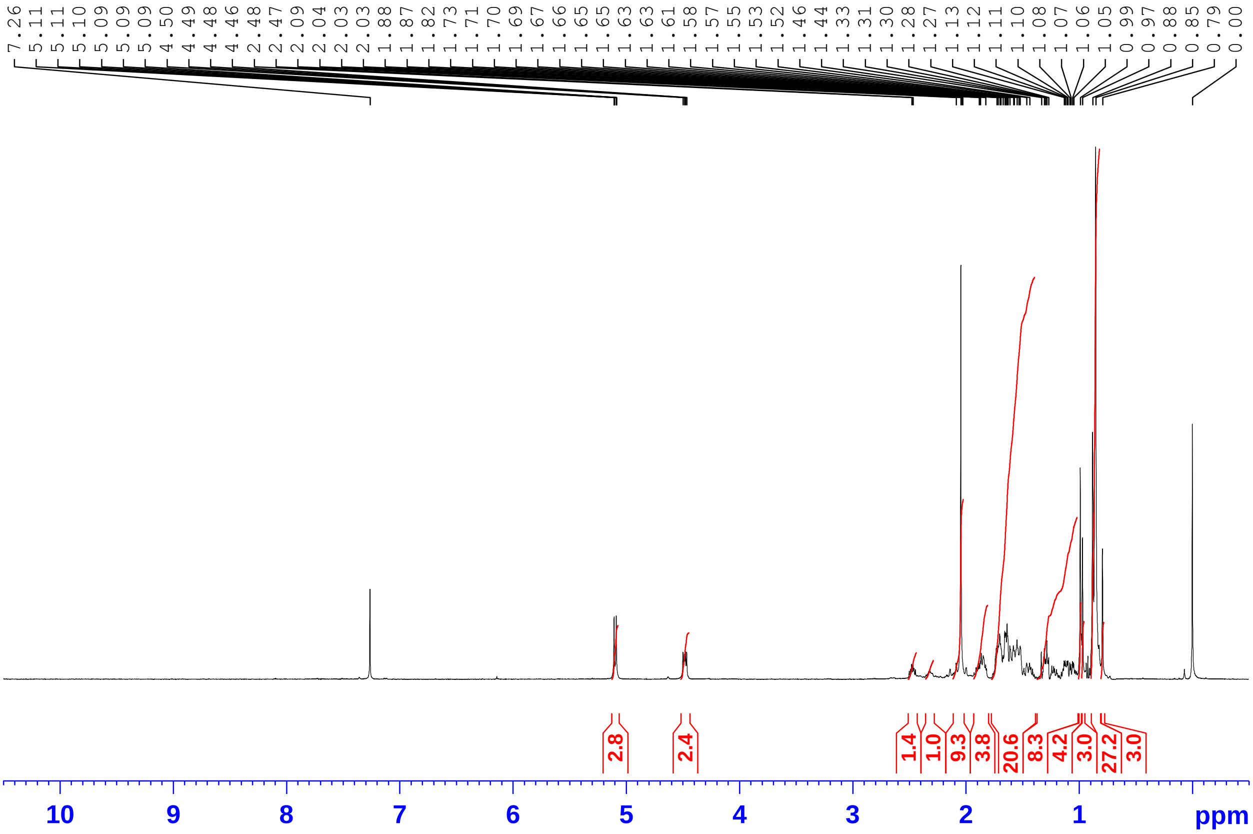

Figure S33. Complete ^{1}H NMR spectrum of mixture of compounds **6 and 7** in CDCl3, 500MHz.

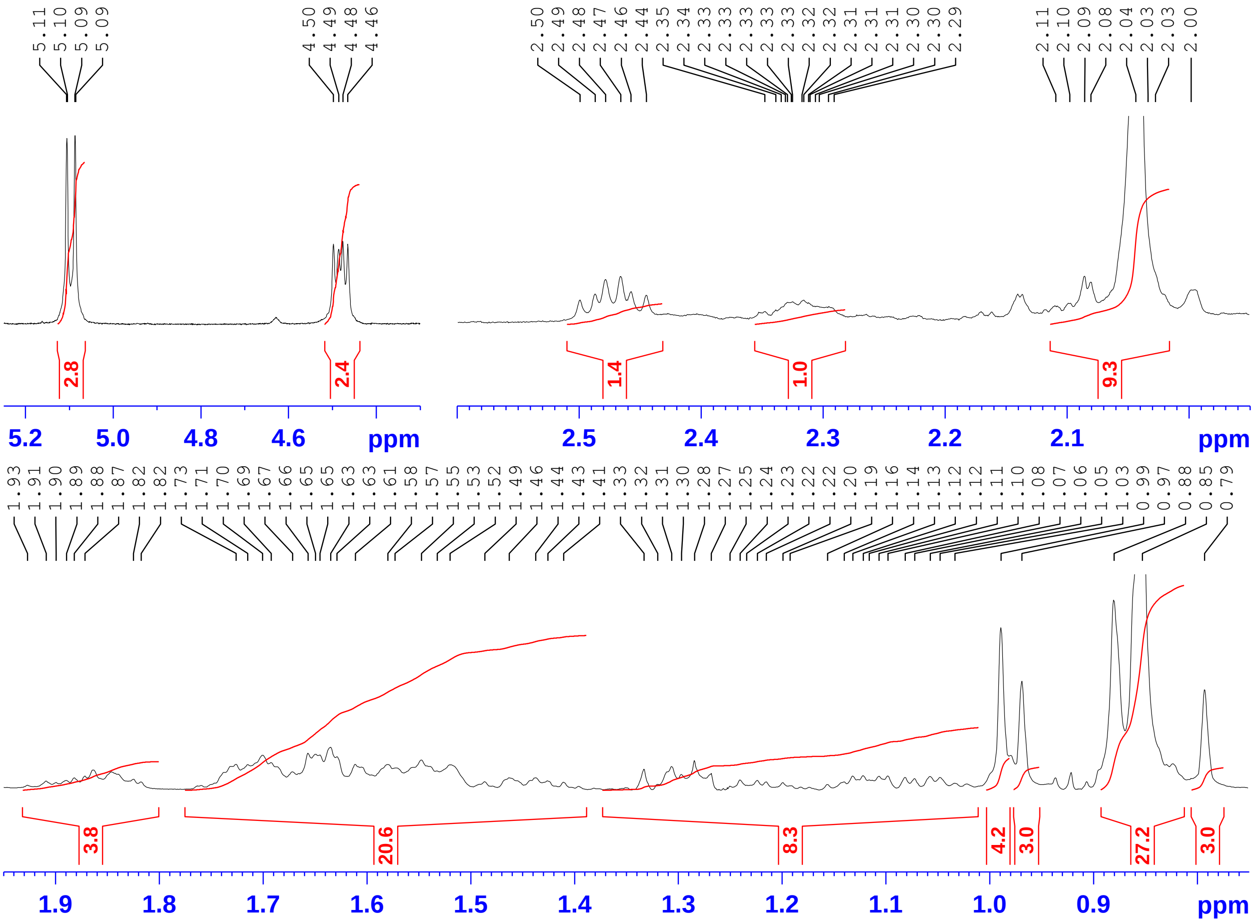

Figure S34. Expanded ^{1}H NMR spectrum of mixture of compounds **6 and 7** in $CDCl_3$, 500MHz.

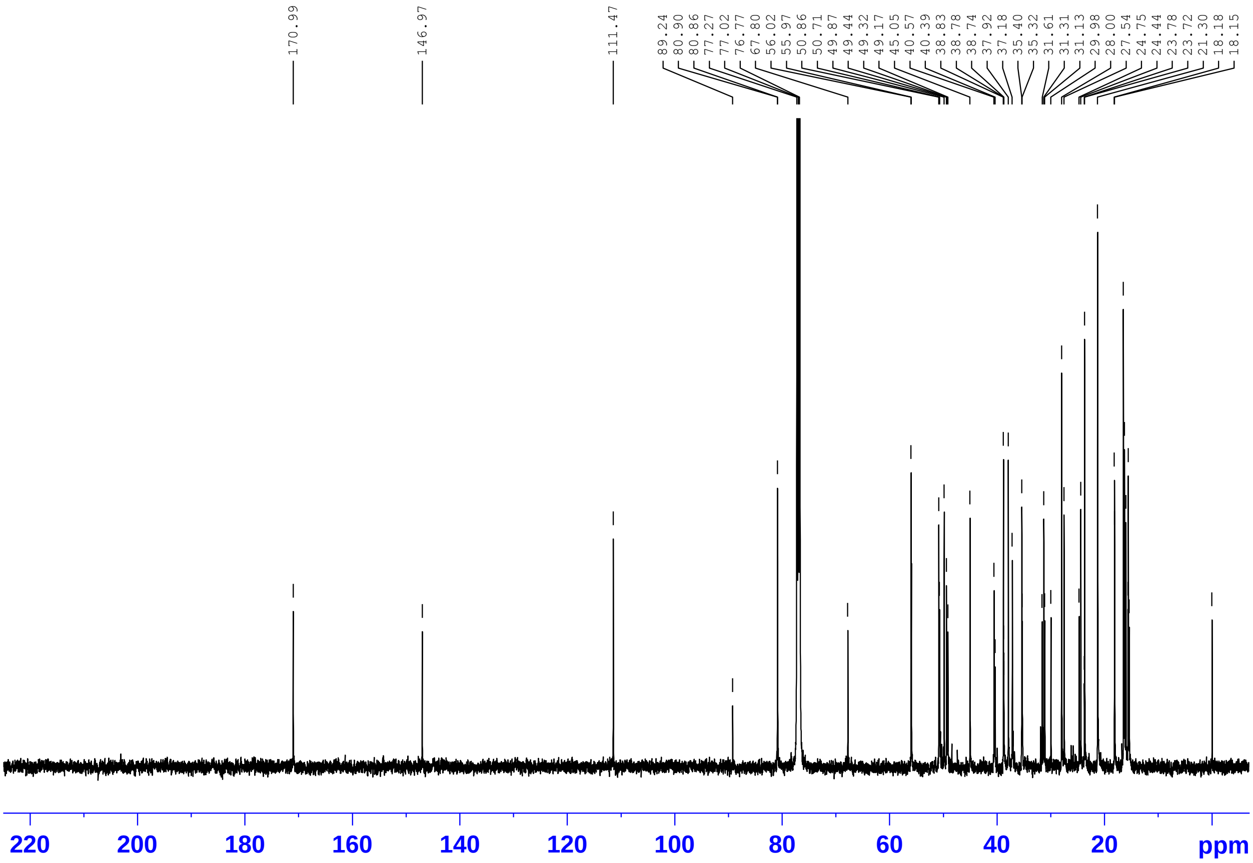

Figure S35. Complete $^{13}C\{^{1}H\}$ spectrum of mixture of compounds **6 and 7** in $CDCl_3$, 125 MHz.

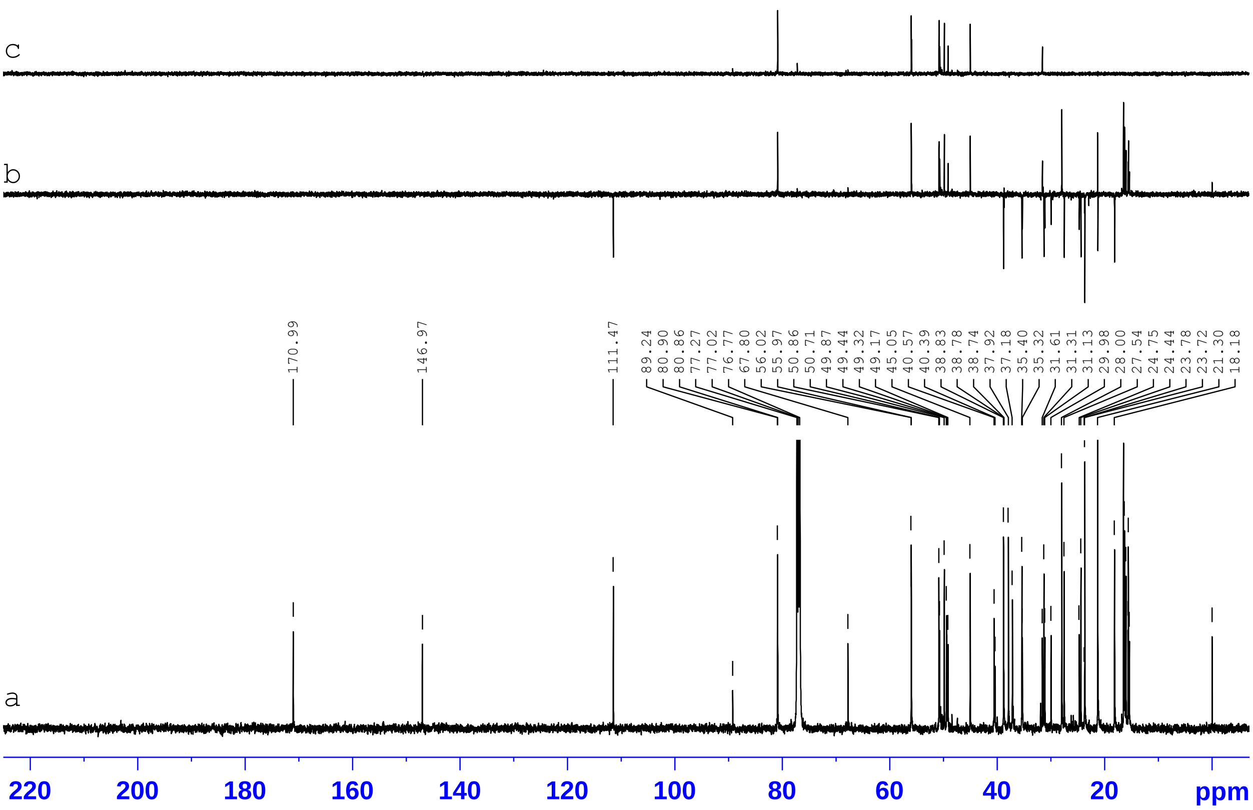

Figure S36. $^{13}C\{^{1}H\}$ and DEPT-135, DEPT-90 spectra of mixture of compounds **6 and 7** in $CDCl_3$, 125 MHz.

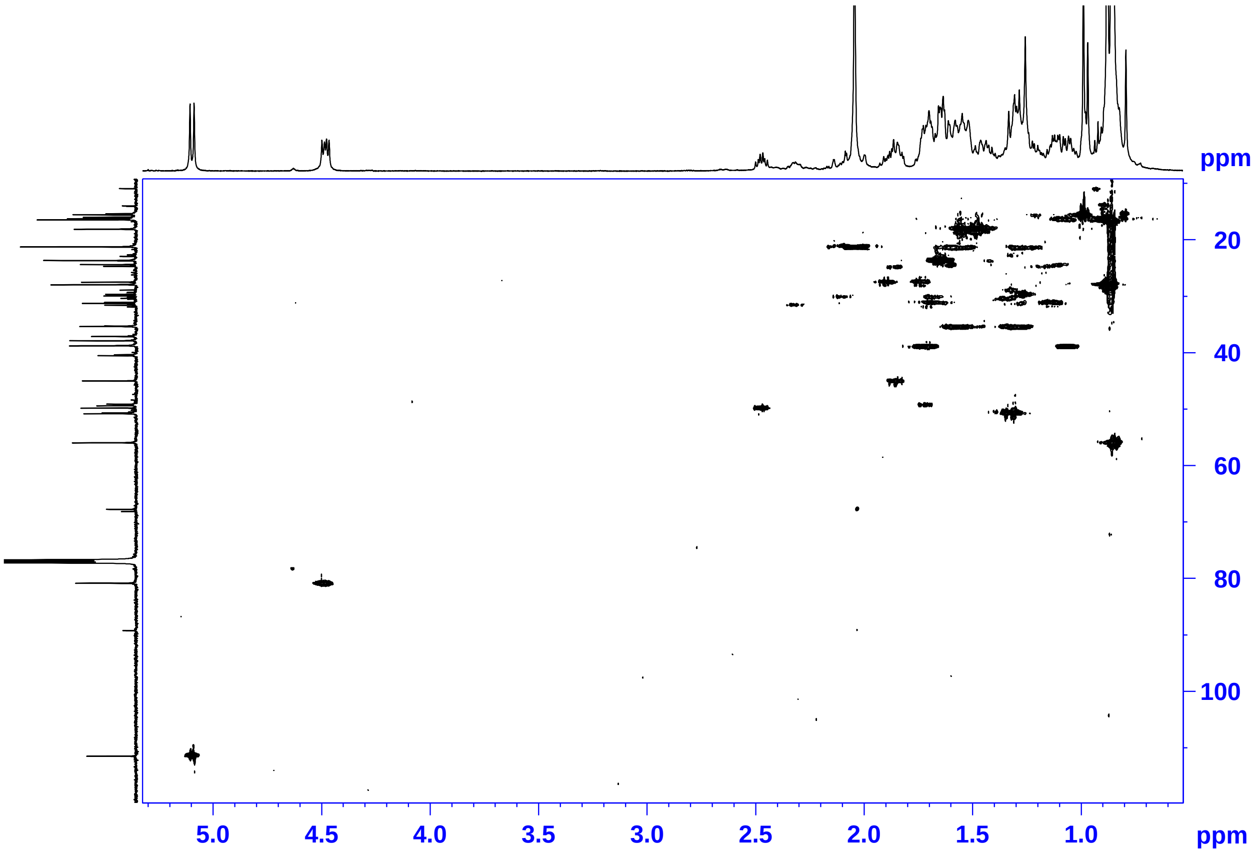

Figure S37. {^{1}H, ^{13}C} HSQC spectrum of mixture of compounds **6 and 7** in $CDCl_3$, 500 MHz.

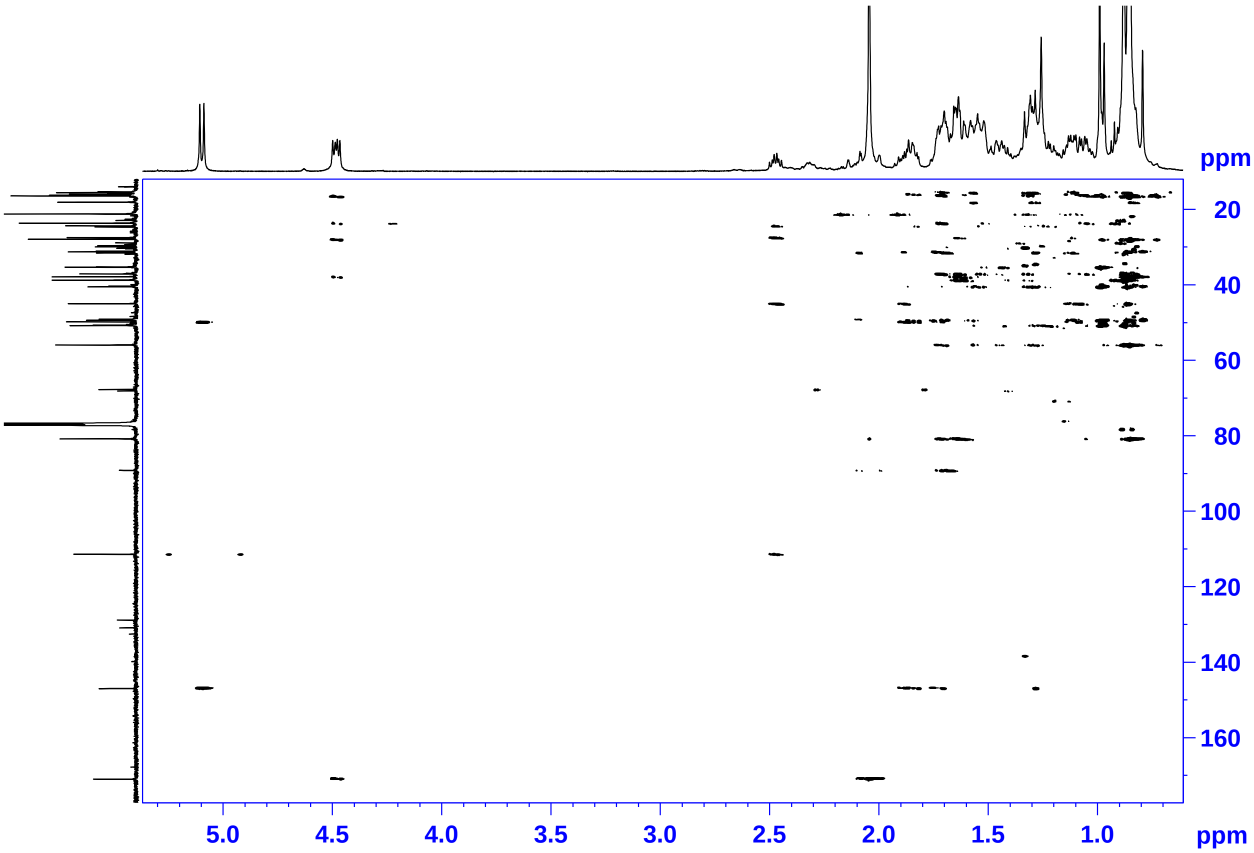

Figure S38. {^{1}H, ^{13}C} HMBC spectrum of mixture of compounds **6 and 7** in $CDCl_3$, 500 MHz.

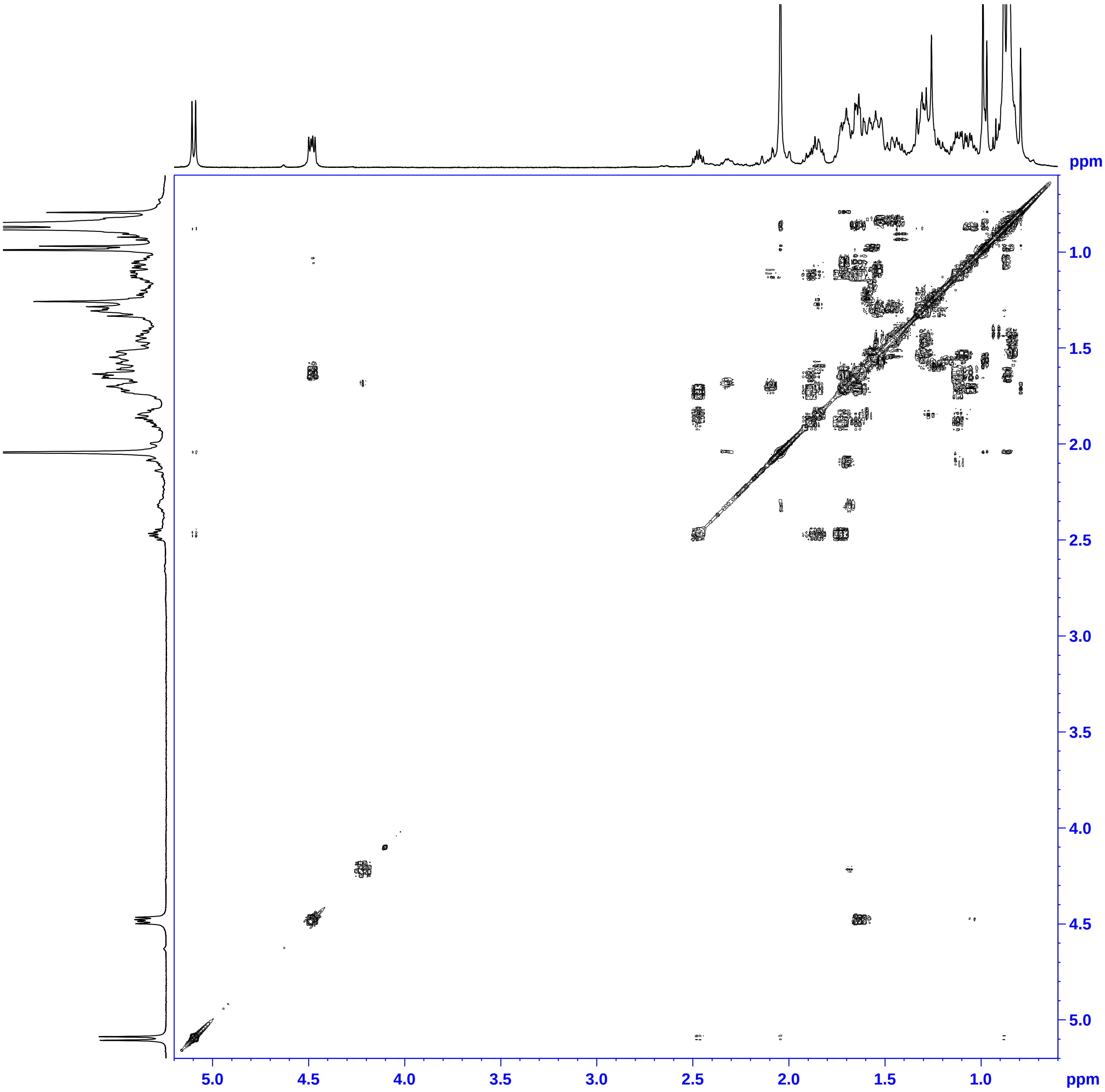

Figure S39. {^{1}H, ^{1}H} COSY spectrum of mixture of compounds **6 and 7** in $CDCl_3$, 500 MHz.

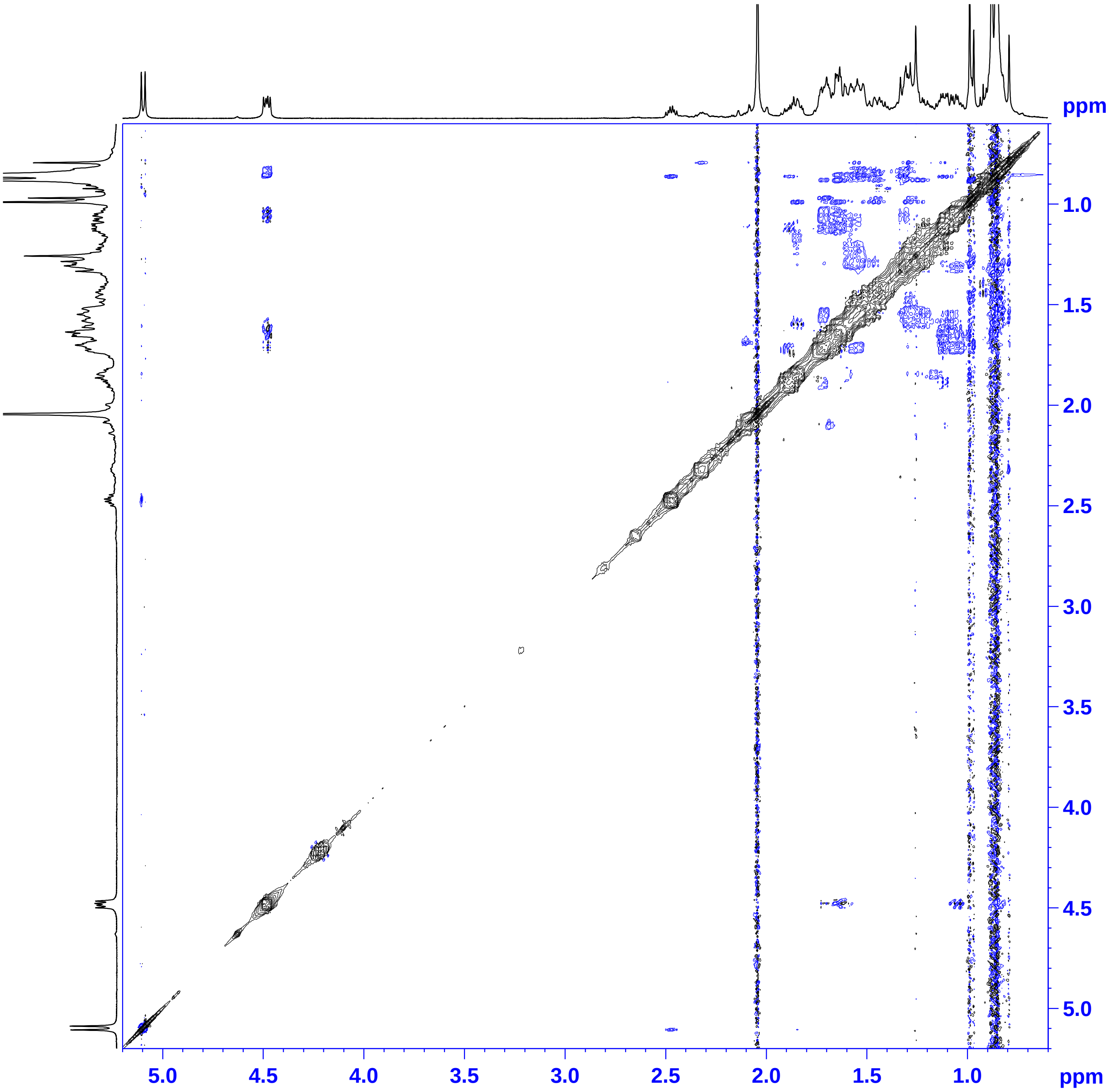

Figure S40. {^{1}H, ^{1}H} NOESY spectrum of mixture of compounds **6 and 7** in $CDCl_3$, 500 MHz.

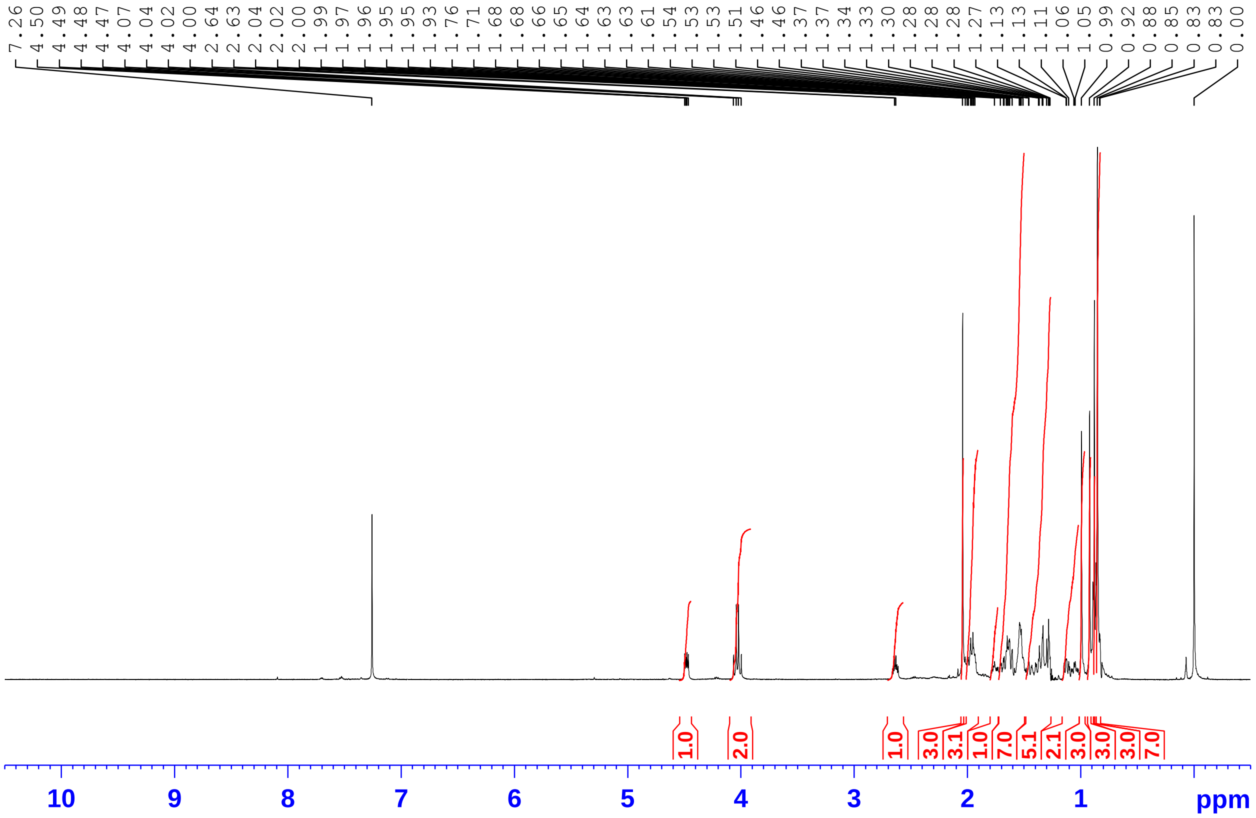

Figure S41. Complete ^{1}H NMR spectrum of compound **8** in $CDCl_3$, 500MHz.

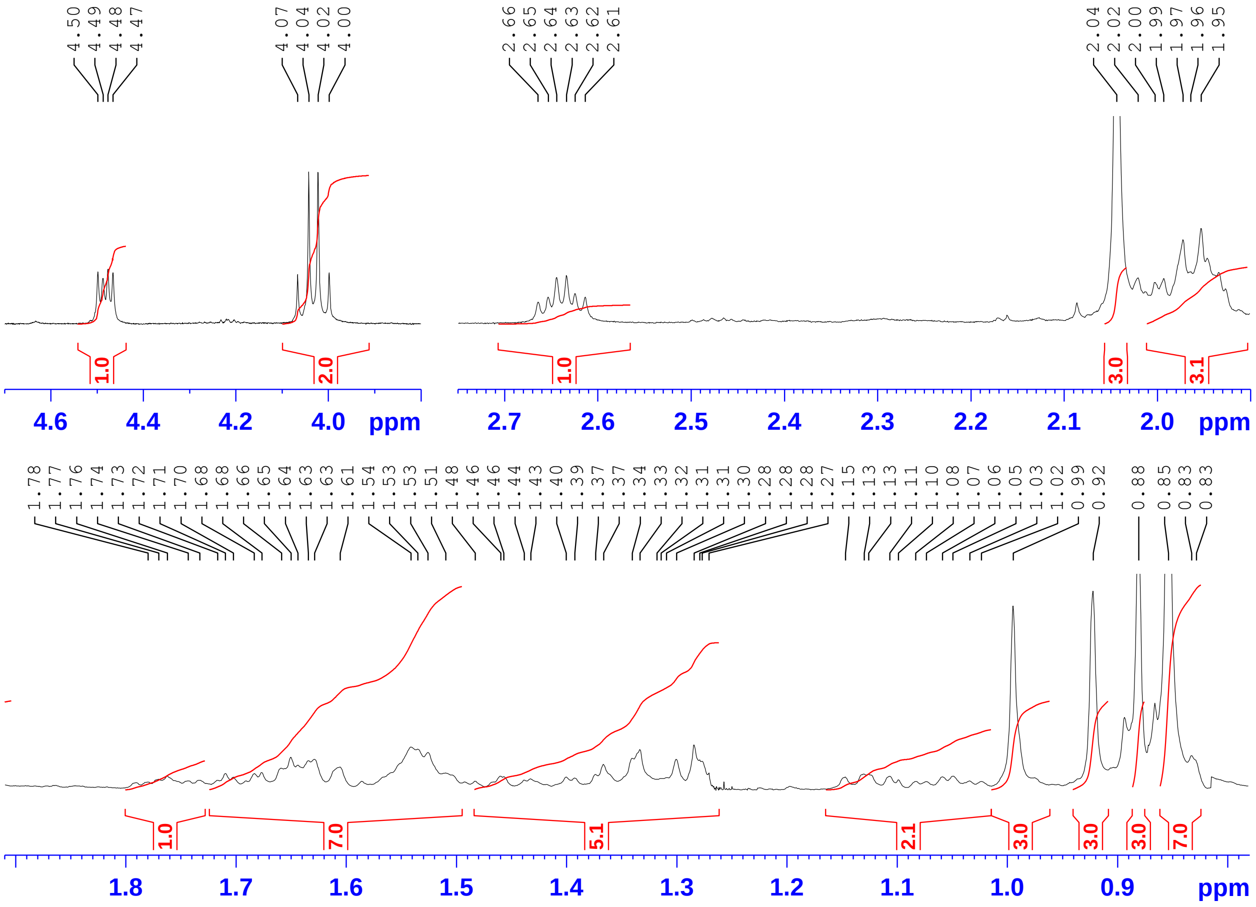

Figure S42. Expanded ^{1}H NMR spectrum of compound **8** in $CDCl_3$, 500MHz.

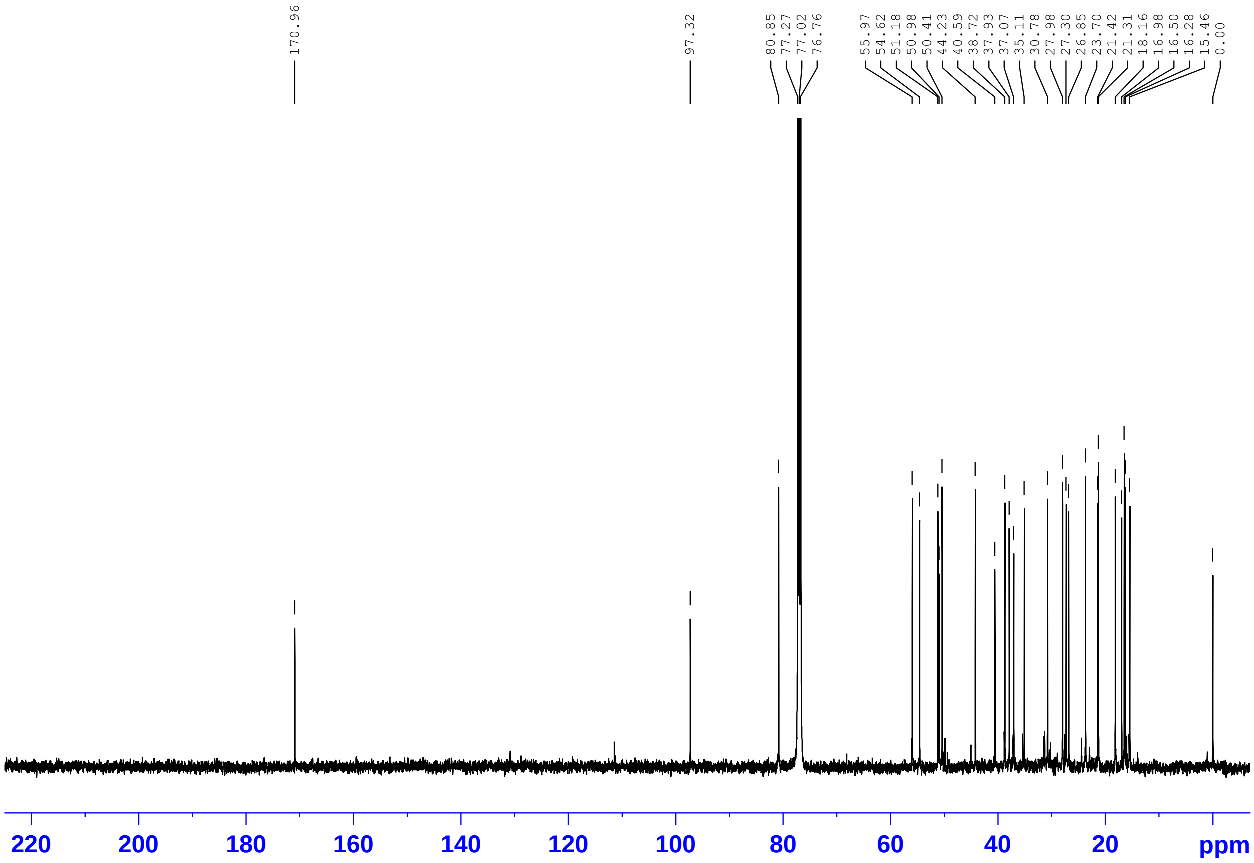

Figure S43. Complete $^{13}C\{^{1}H\}$ spectrum of compound **8** in $CDCl_3$, 125 MHz.

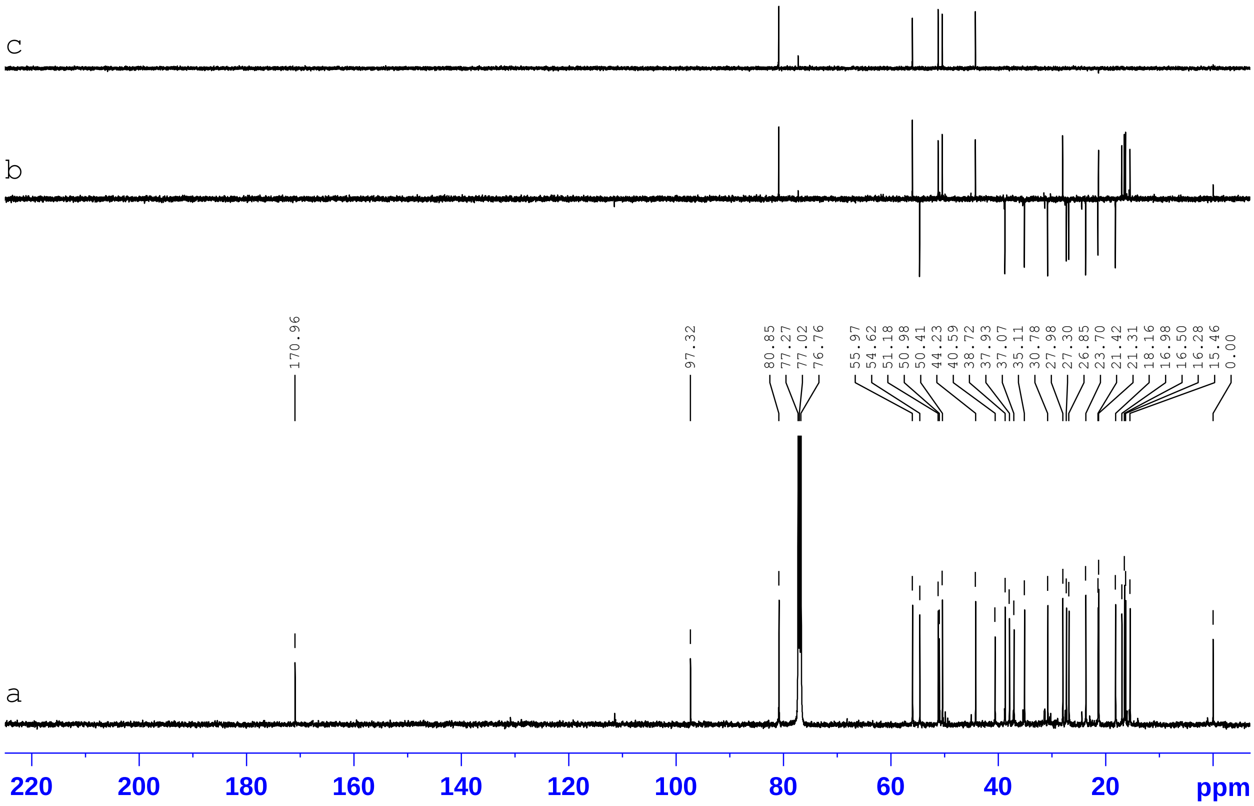

Figure S44. $^{13}C\{^{1}H\}$ and DEPT-135, DEPT-90 spectra of compound **8** in $CDCl_3$, 125 MHz.

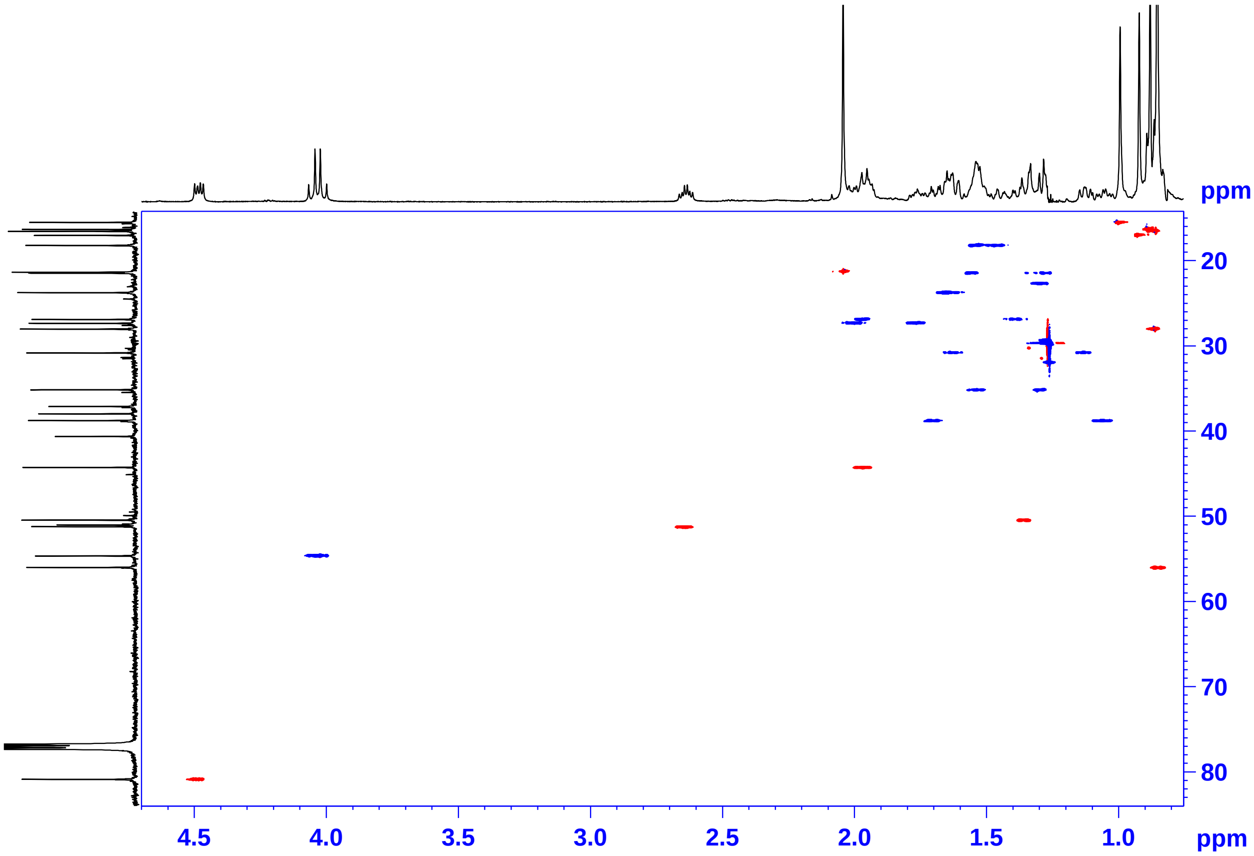

Figure S45. {^{1}H, ^{13}C} HSQCed spectrum of compound **8** in $CDCl_3$, 500 Hz.

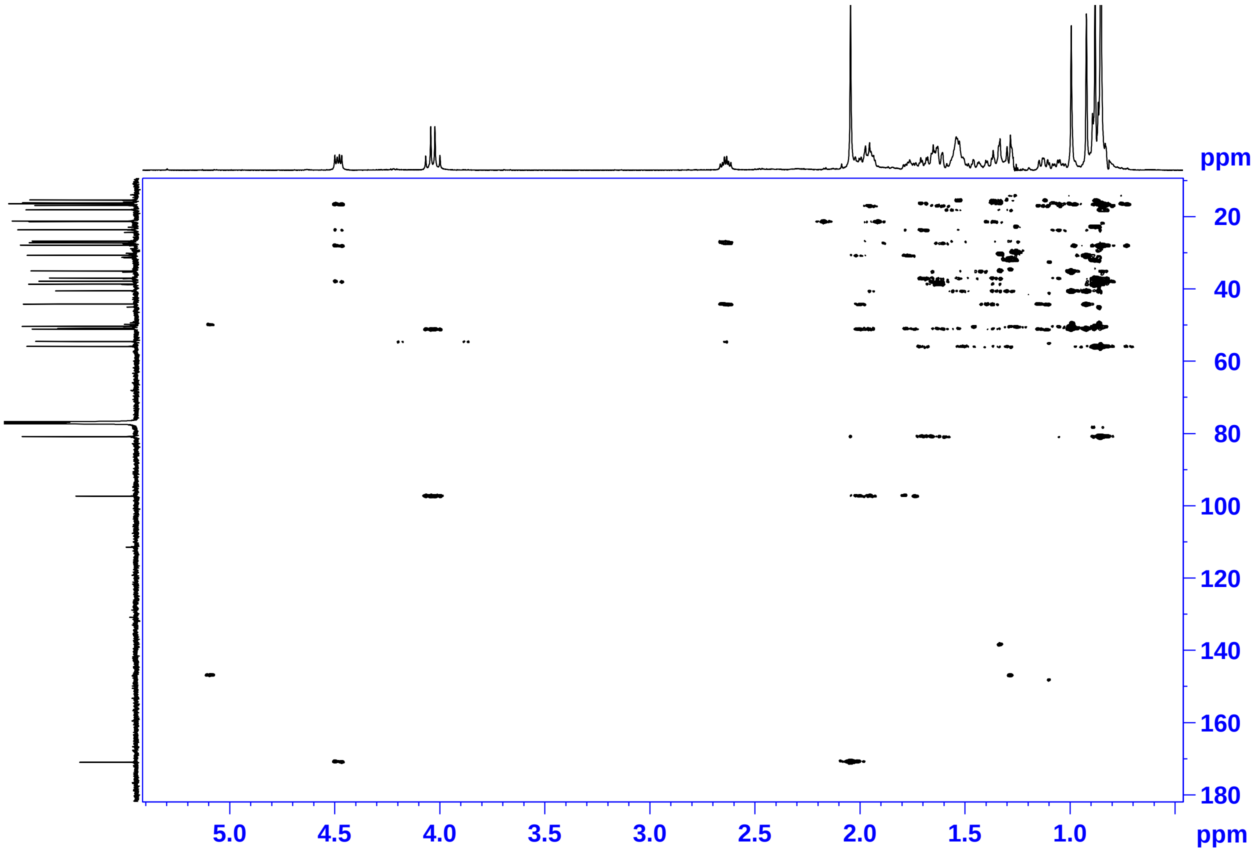

Figure S46. {^{1}H, ^{13}C} HMBC spectrum of compound **8** in $CDCl_3$, 500 MHz.

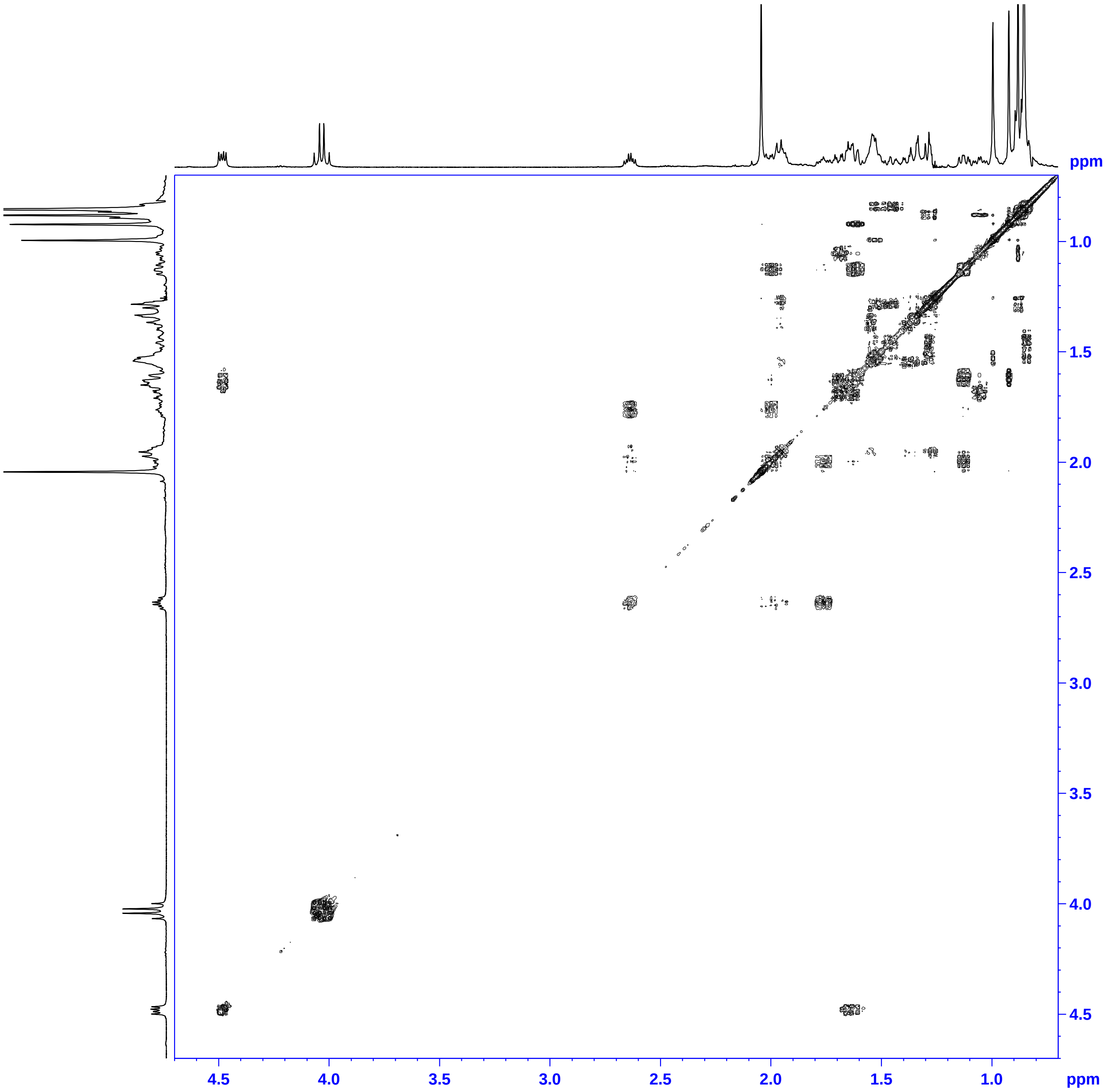

Figure S47. {¹H, ¹H} COSY spectrum of compound **8** in $CDCl_3$, 500 MHz.

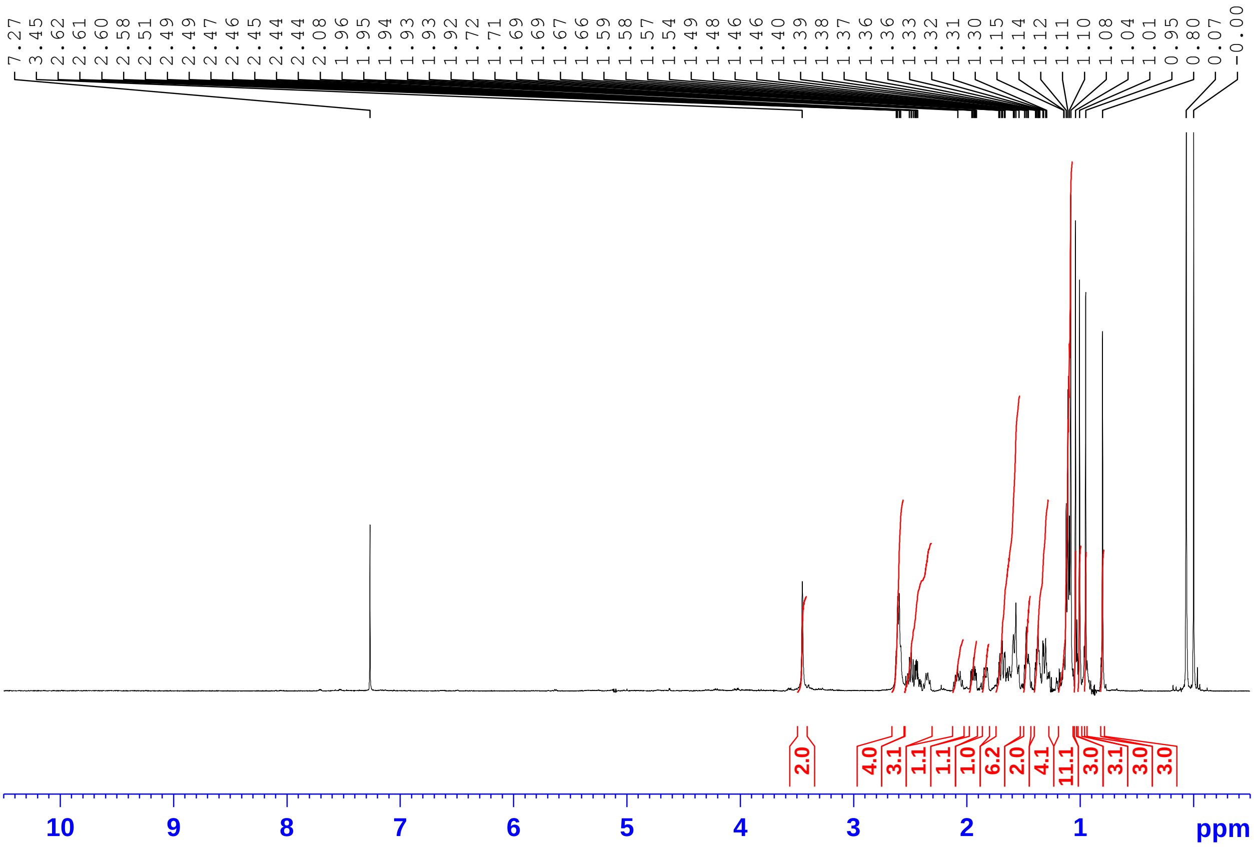

Figure S48. Complete $^{13}C\{^{1}H\}$ spectrum of compound **9a** in $CDCl_3$, 125 MHz.

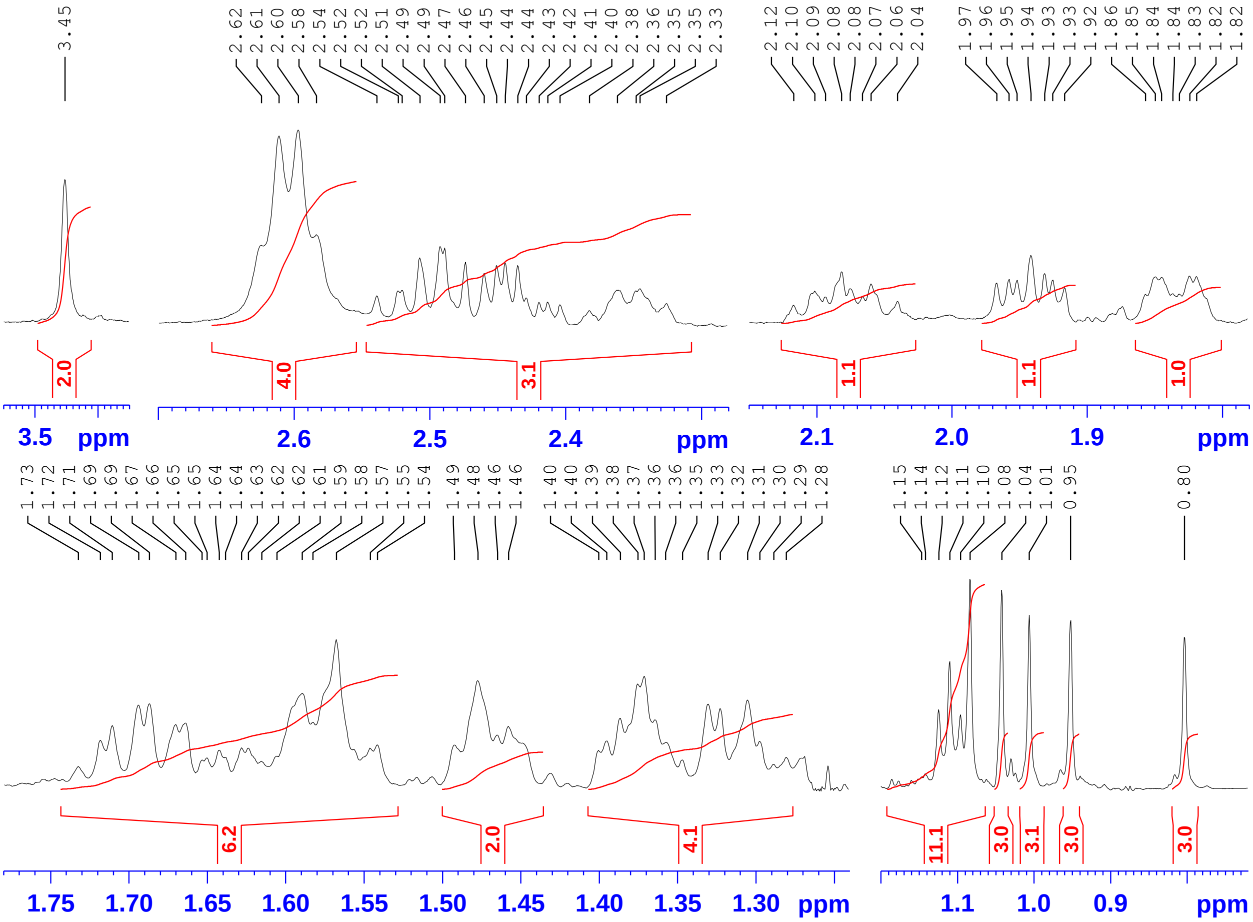

Figure S49. Expanded ^{1}H NMR spectrum of compound **9a** in $CDCl_3$, 500MHz.

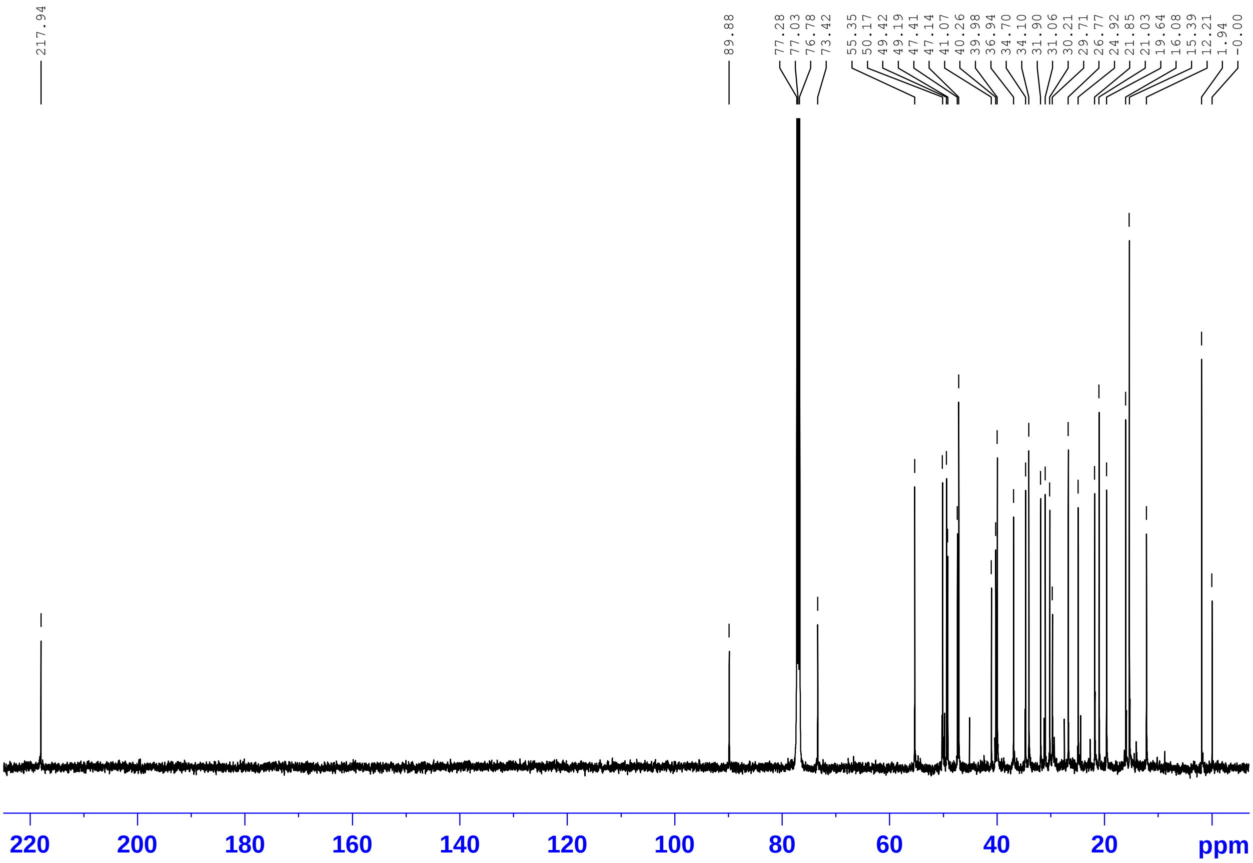

Figure S50. Complete $^{13}C\{^{1}H\}$ spectrum of compound **9a** in $CDCl_3$, 125 MHz.

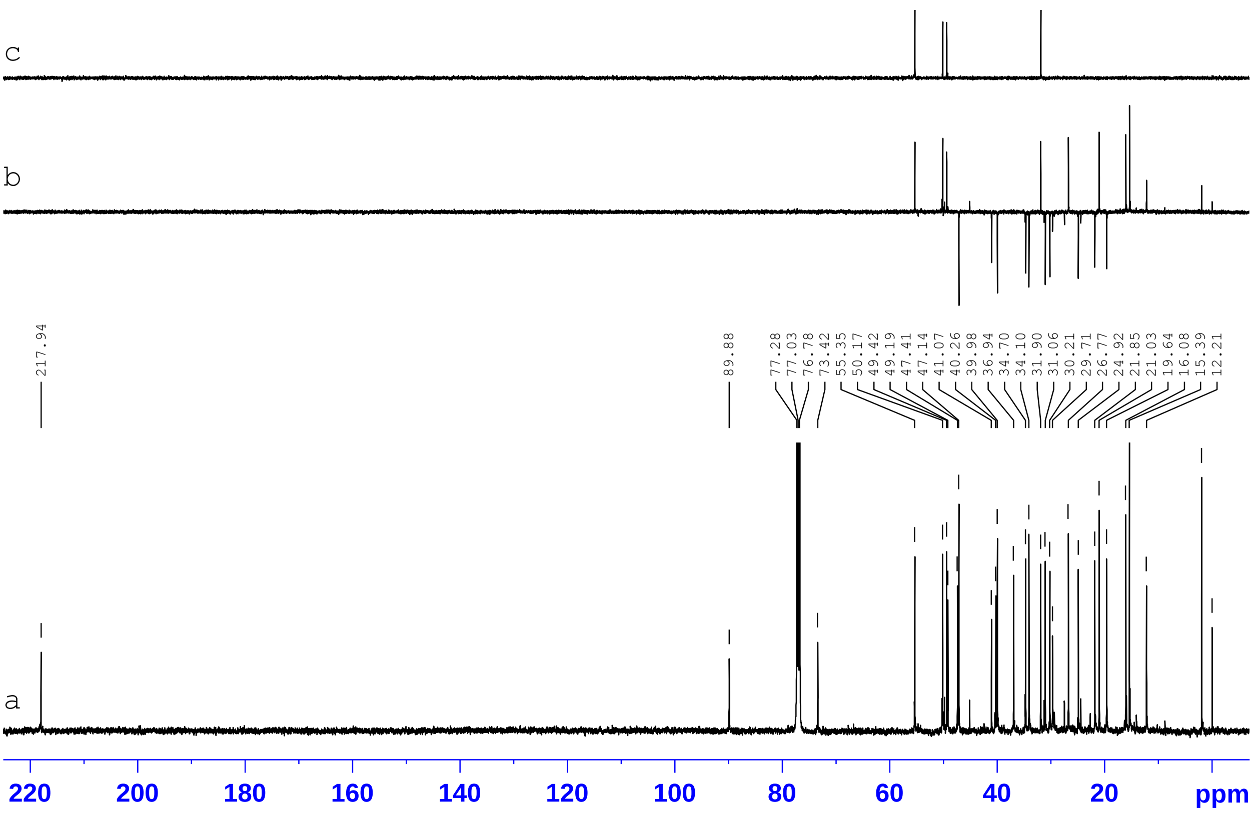

Figure S51. $^{13}C\{^{1}H\}$ (a), DEPT-135 (b) and DEPT-90 (c) spectra of compound **9a** in $CDCl_3$, 125 MHz.

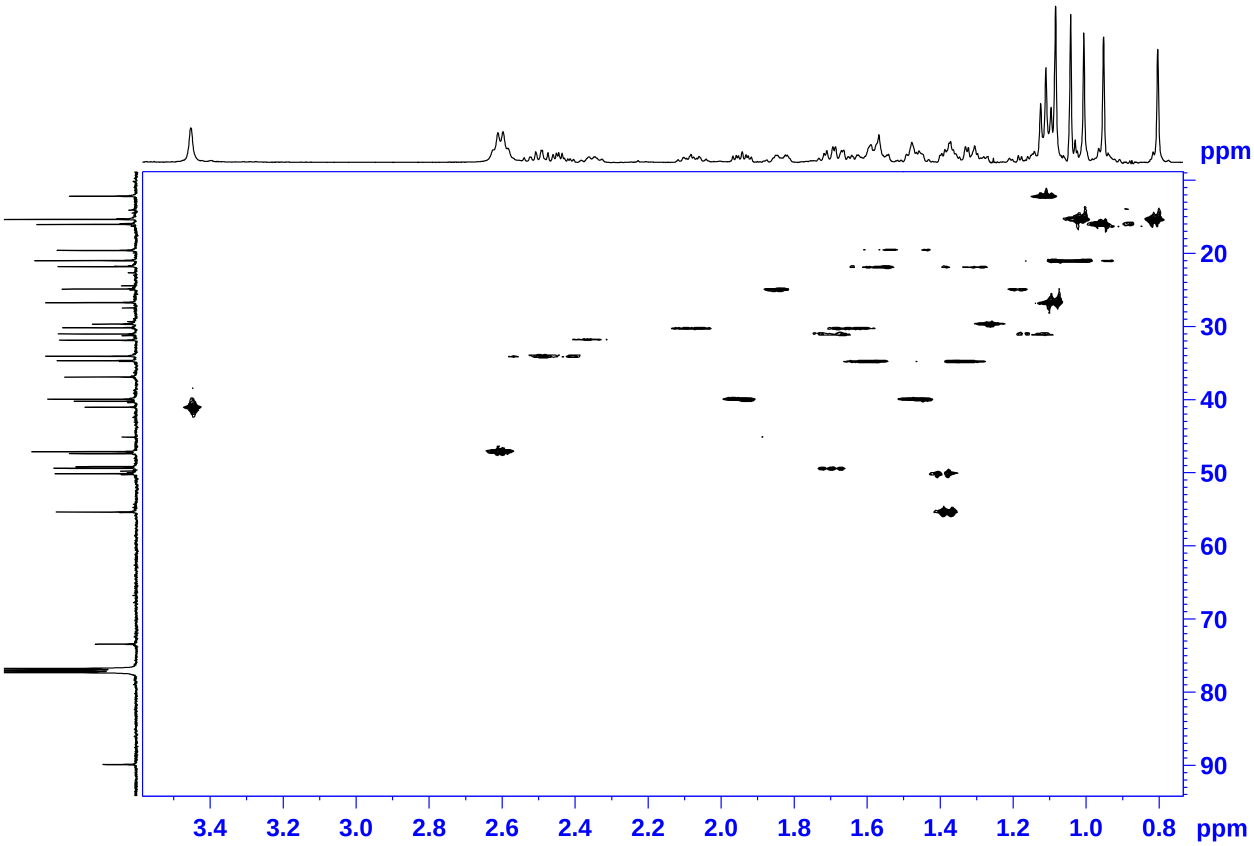

Figure S52. {^{1}H, ^{13}C} HSQC spectrum of compound **9a** in $CDCl_3$, 500 MHz.

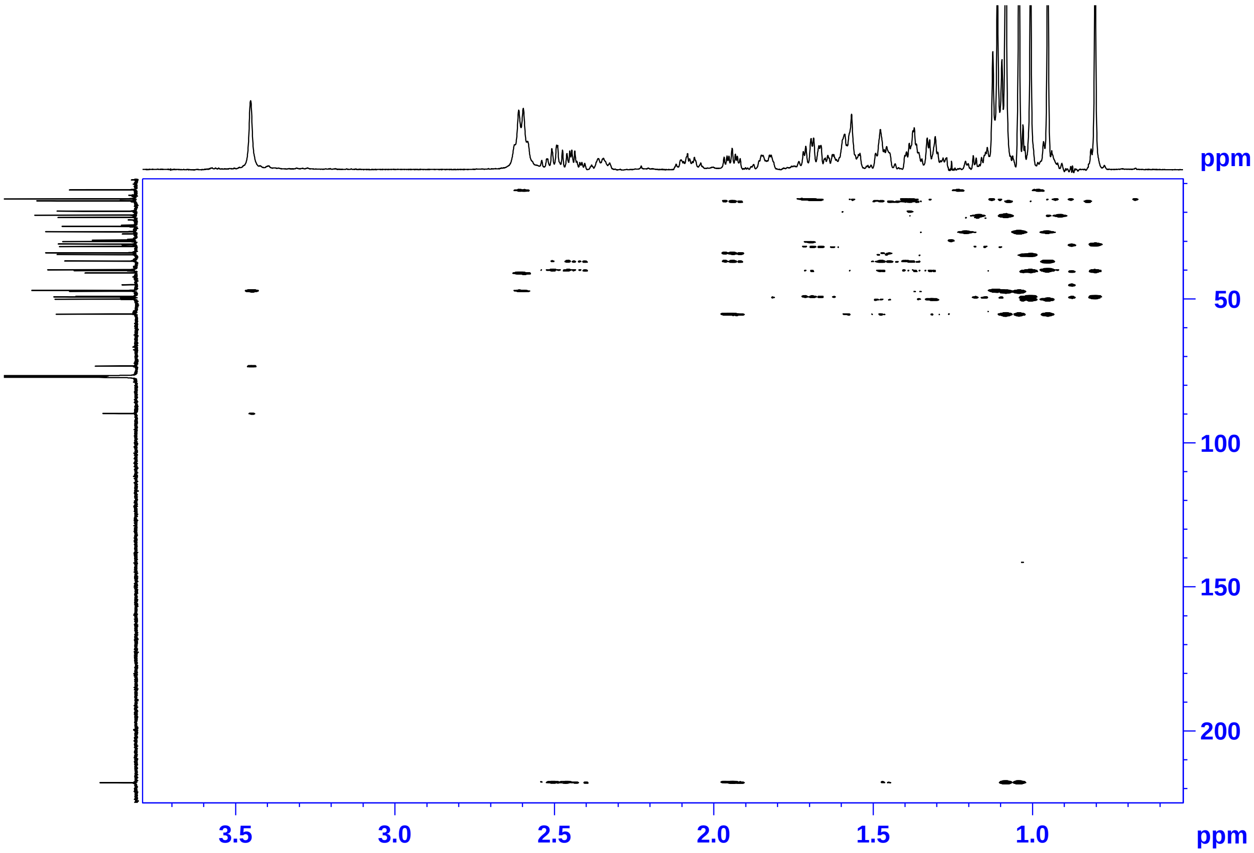

Figure S53. {^{1}H, ^{13}C} HMBC spectrum of compound **9a** in $CDCl_3$, 500 MHz.

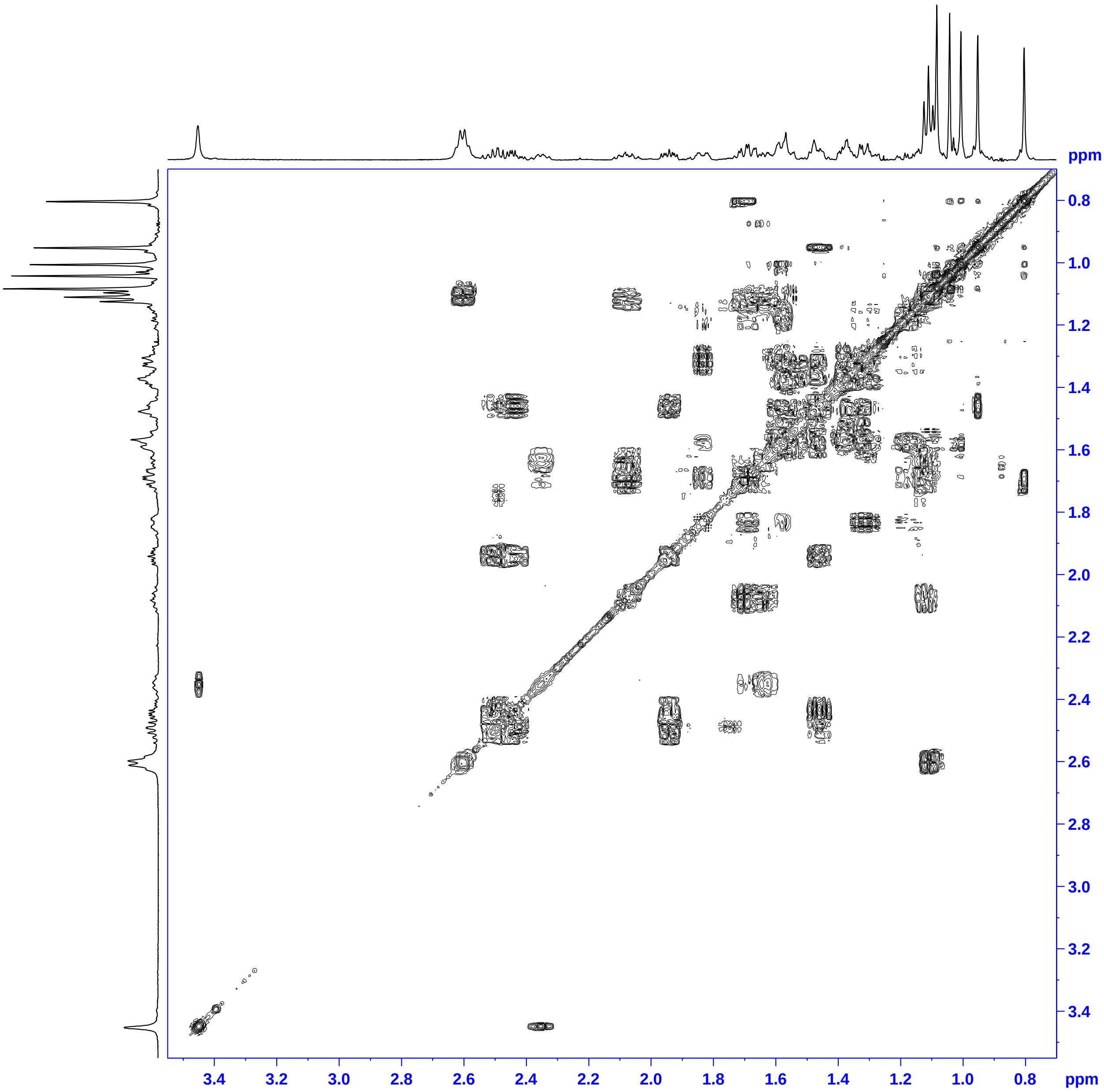

Figure S54. {^{1}H, ^{1}H} COSY spectrum of compound **9a** in $CDCl_3$, 500 MHz.

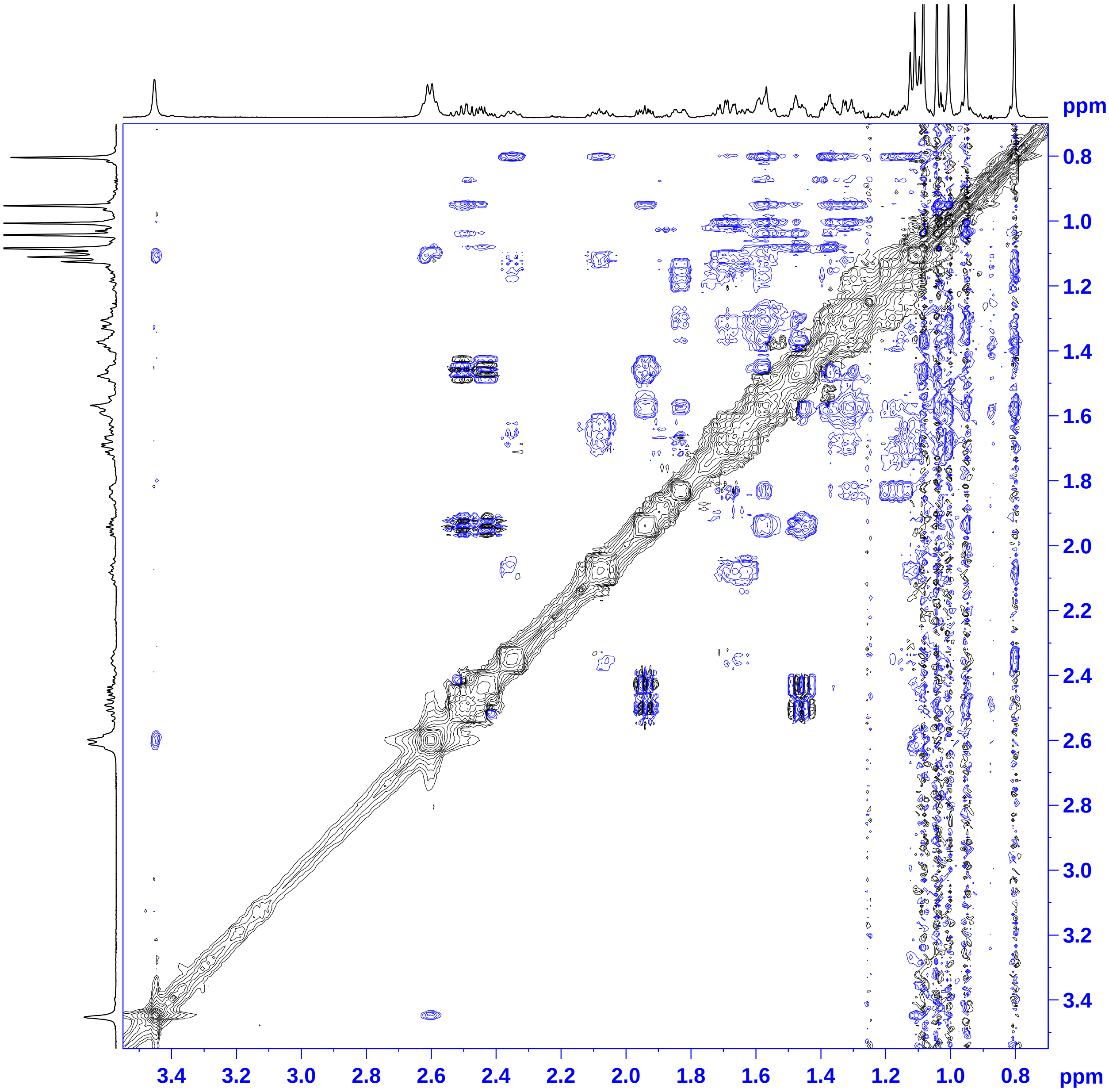

Figure S55. {^{1}H, ^{1}H} NOESY spectrum of compound **9a** in $CDCl_3$ in $CDCl_3$, 500 MHz.

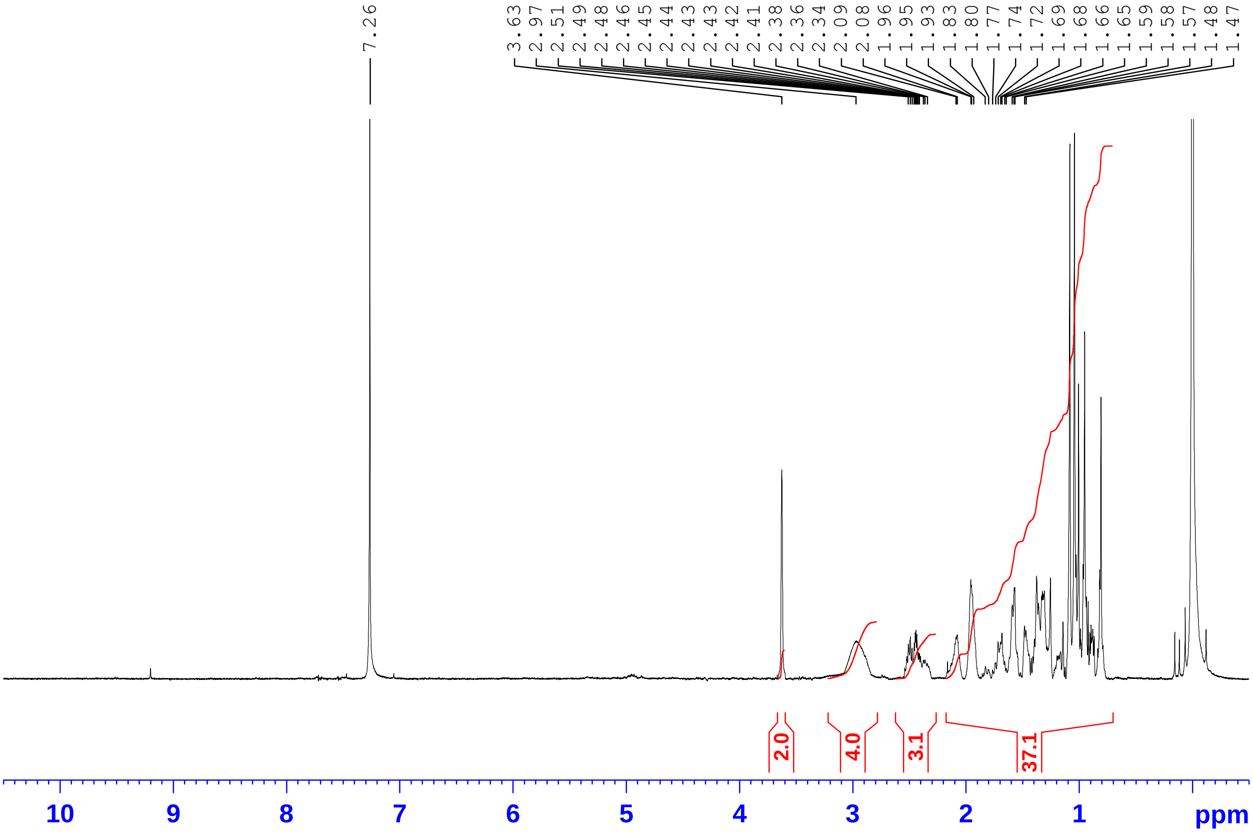

Figure S56. Complete ¹H NMR spectrum of compound **9b** in $CDCl_3$, 500MHz.

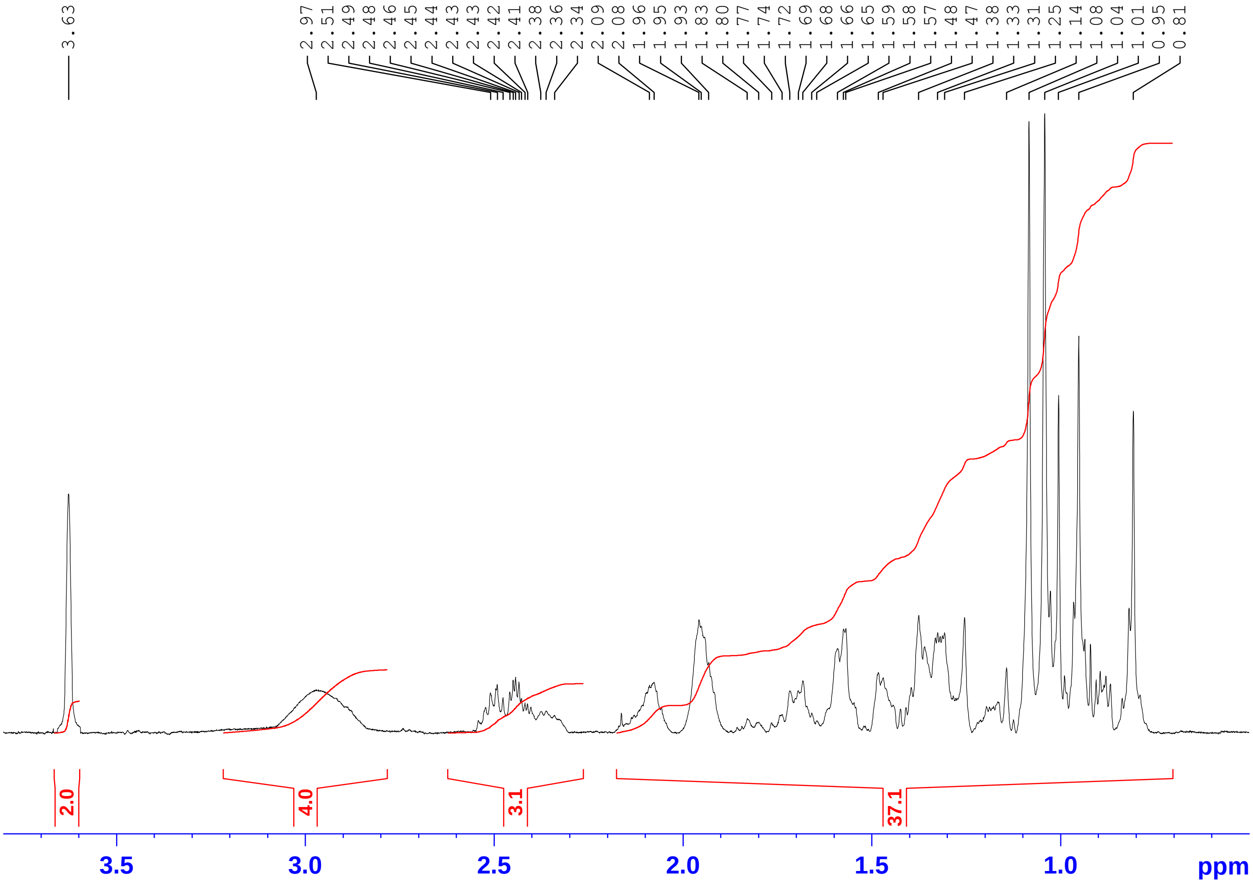

Figure S57. Expanded ¹H NMR spectrum of compound **9b** in $CDCl_3$, 500MHz.

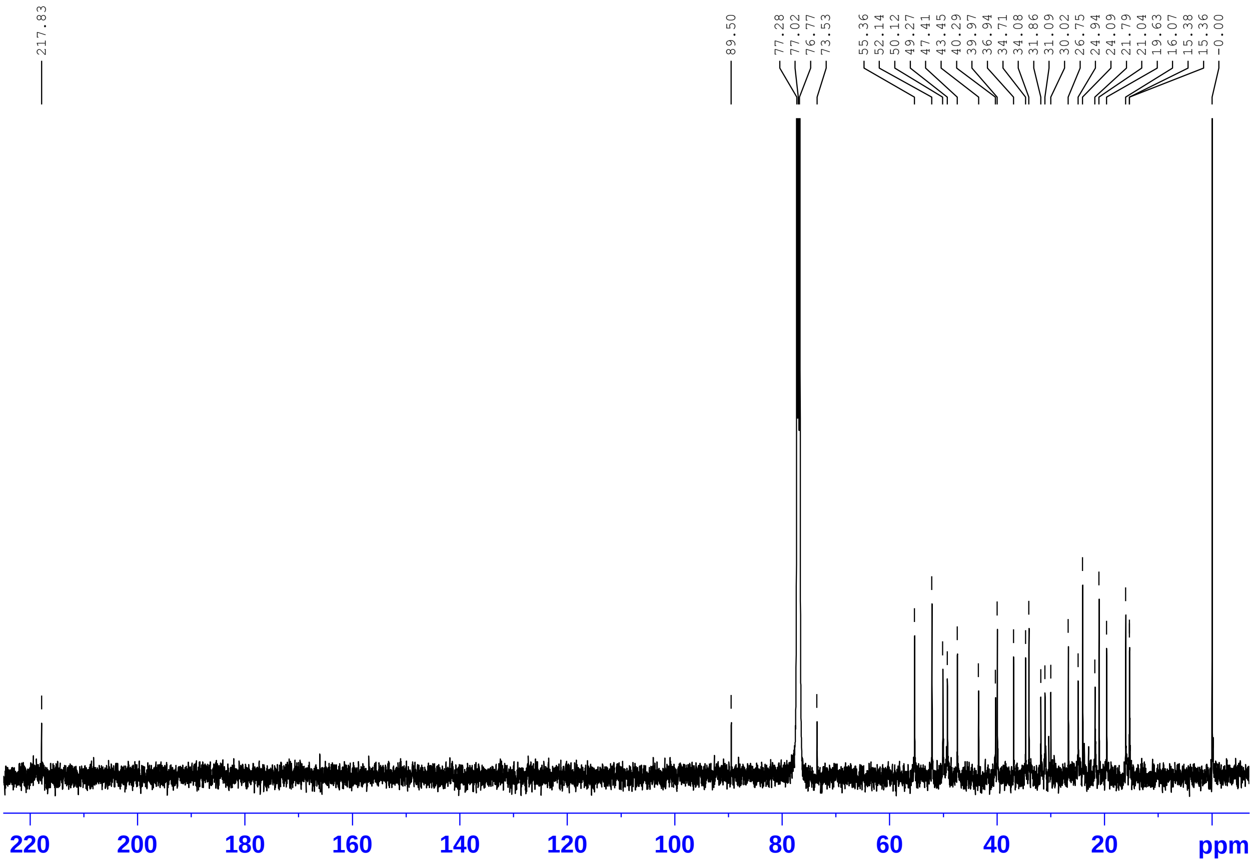

Figure S58. Complete $^{13}C\{^{1}H\}$ spectrum of compound **9b** in $CDCl_3$, 125 MHz.

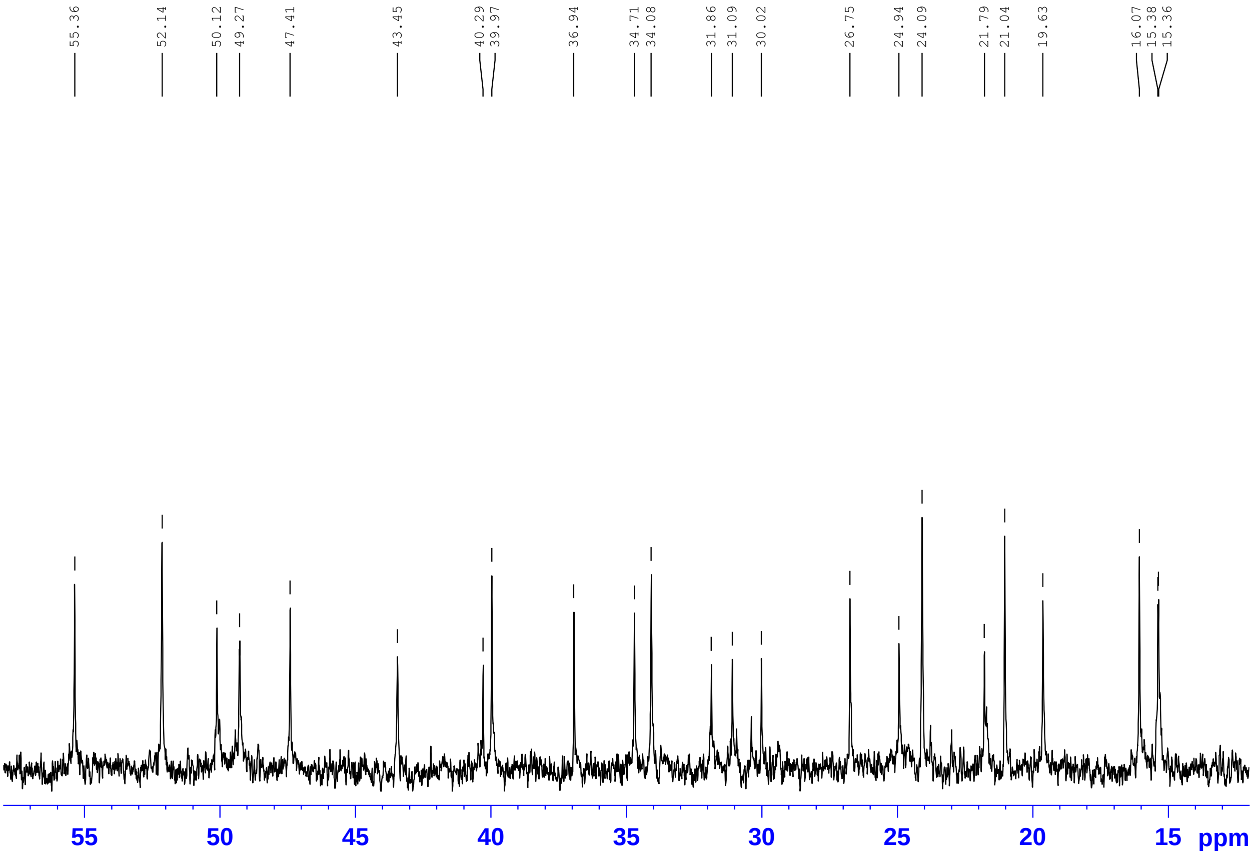

Figure S59. Expanded $^{13}C\{^{1}H\}$ spectrum of compound **9b** in $CDCl_3$, 125 MHz.

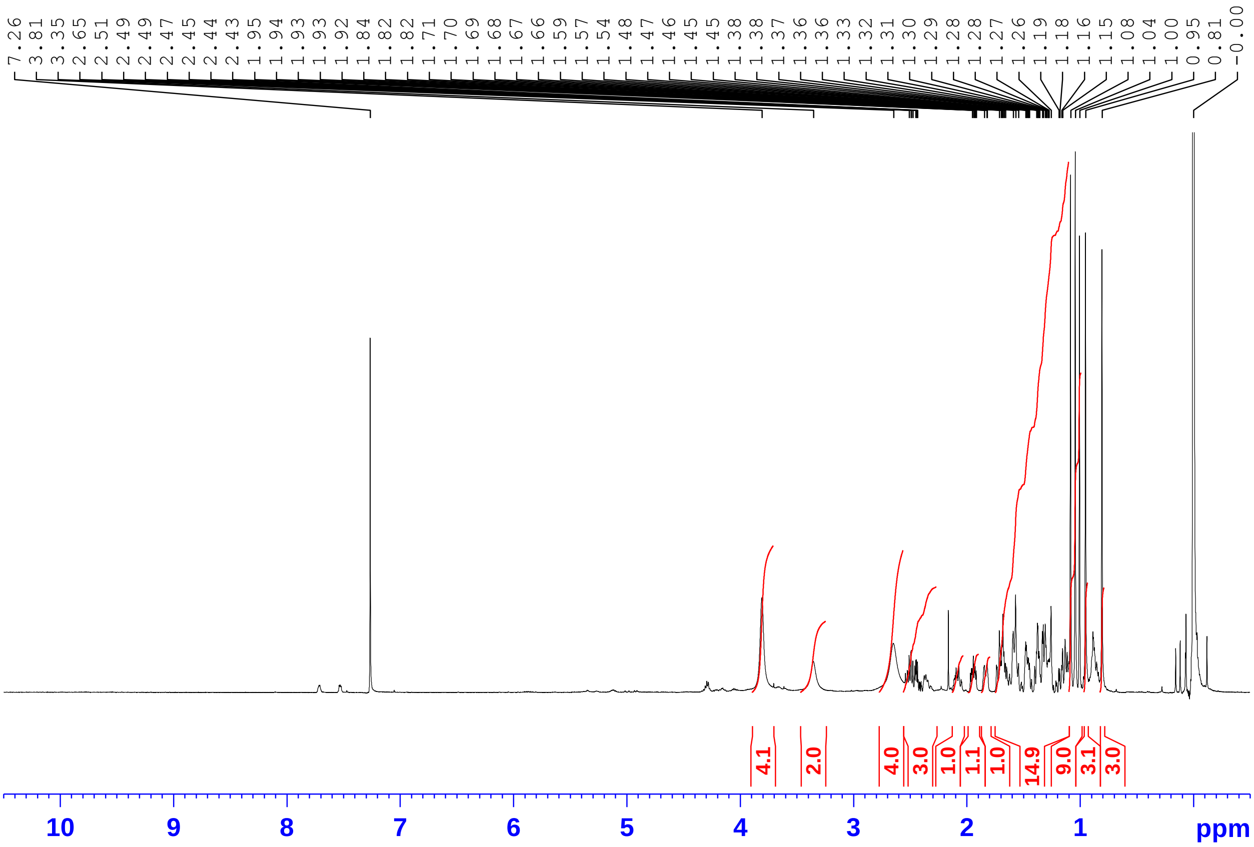

Figure S60. Complete ^{1}H NMR spectrum of compound **9c** in $CDCl_3$, 500MHz.

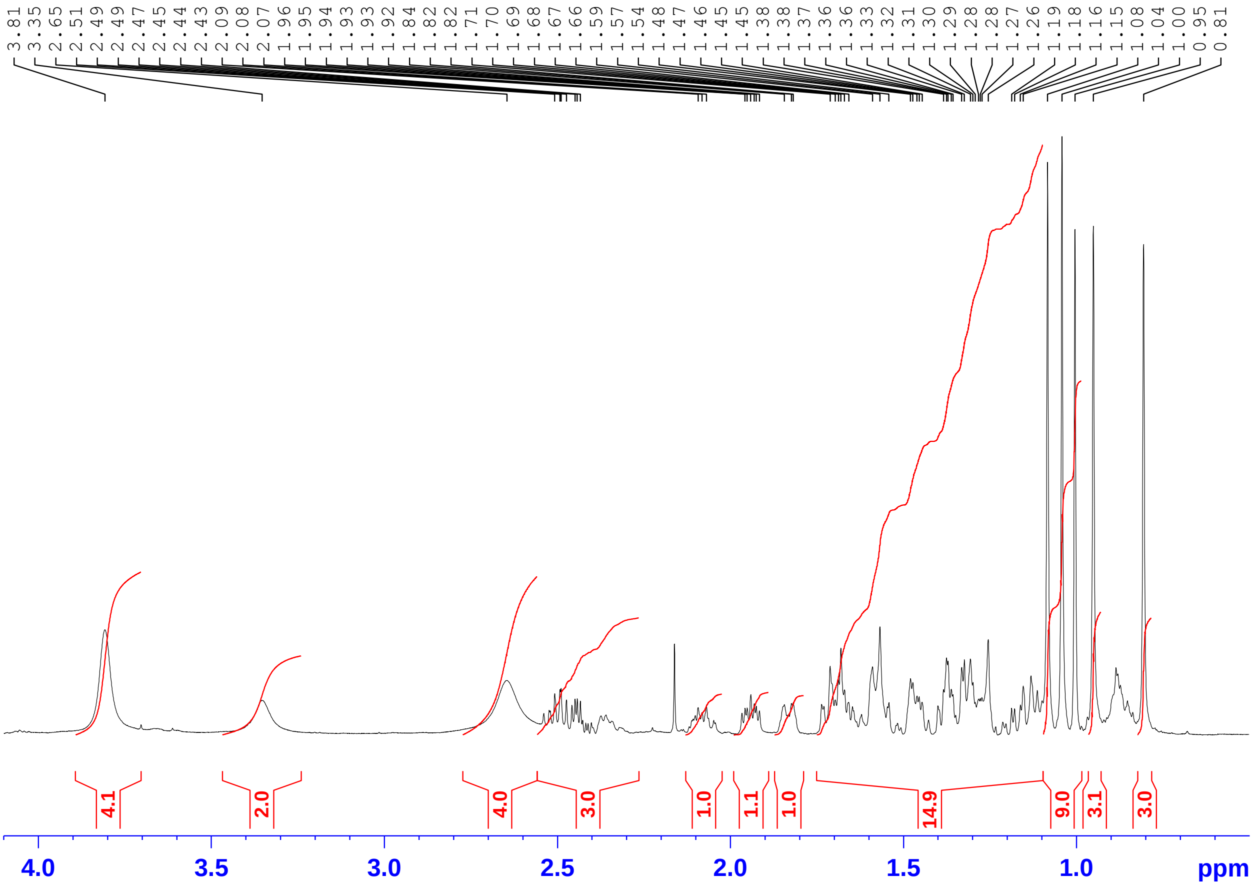

Figure S61. Expanded ^{1}H NMR spectrum of compound **9c** in $CDCl_3$, 500MHz.

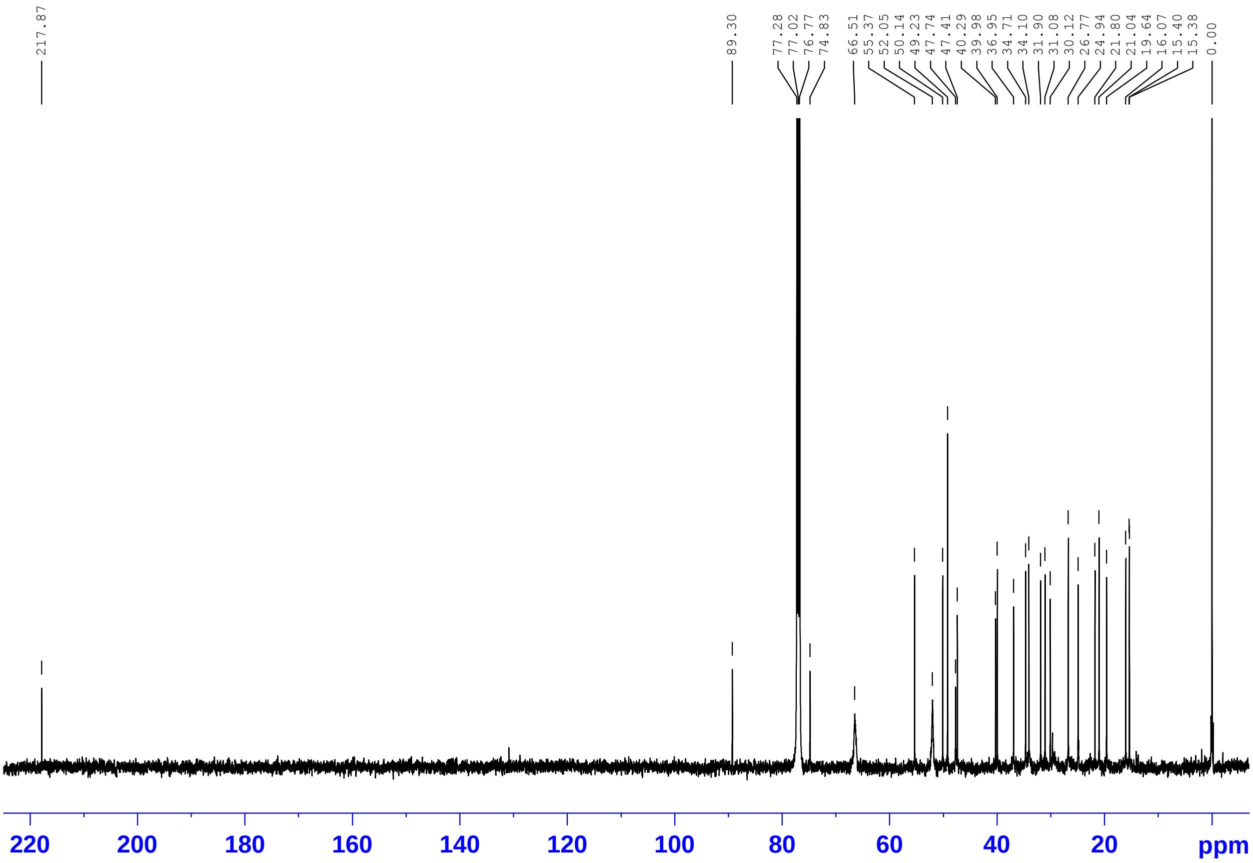

Figure S62. Complete $^{13}C\{^{1}H\}$ spectrum of compound **9c** in $CDCl_3$, 125 MHz.

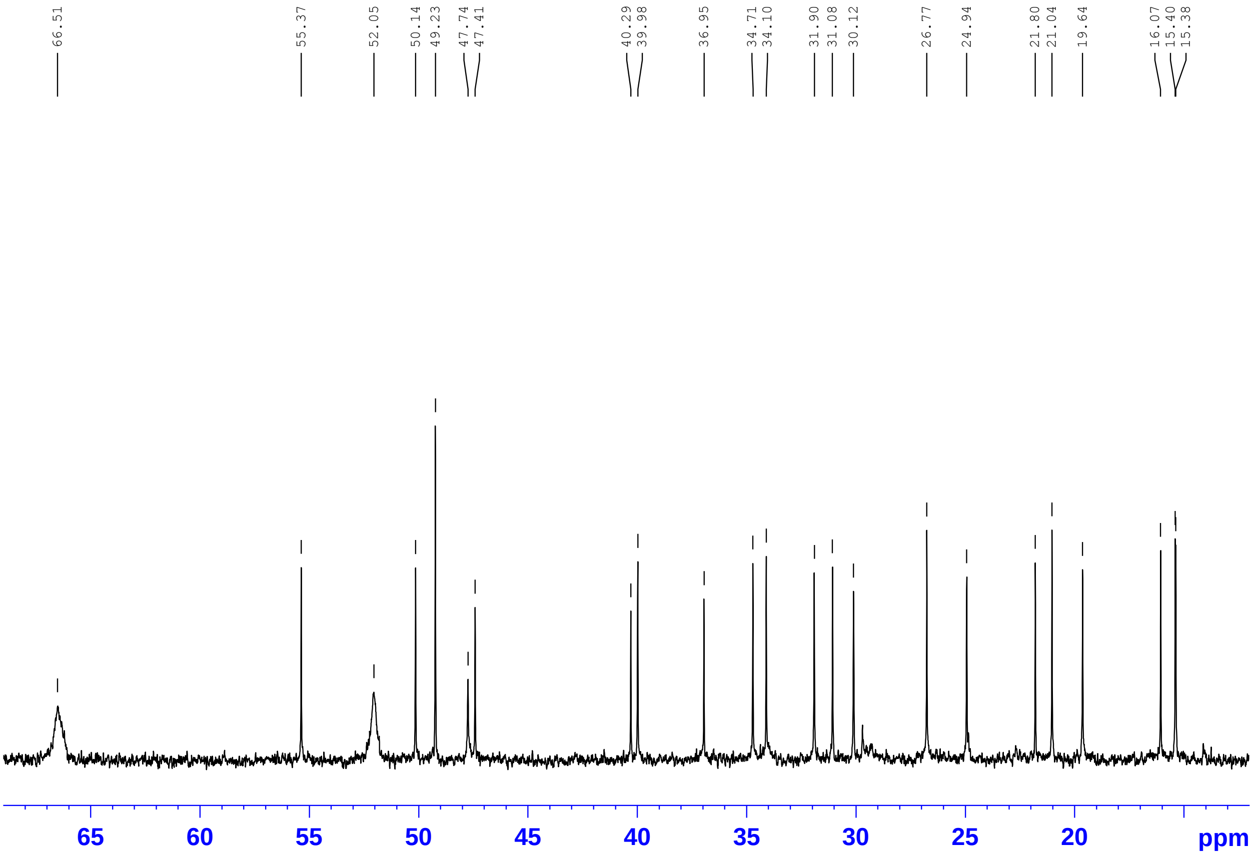

Figure S63. Expanded $^{13}C\{^{1}H\}$ spectrum of compound **9c** in $CDCl_3$, 125 MHz.

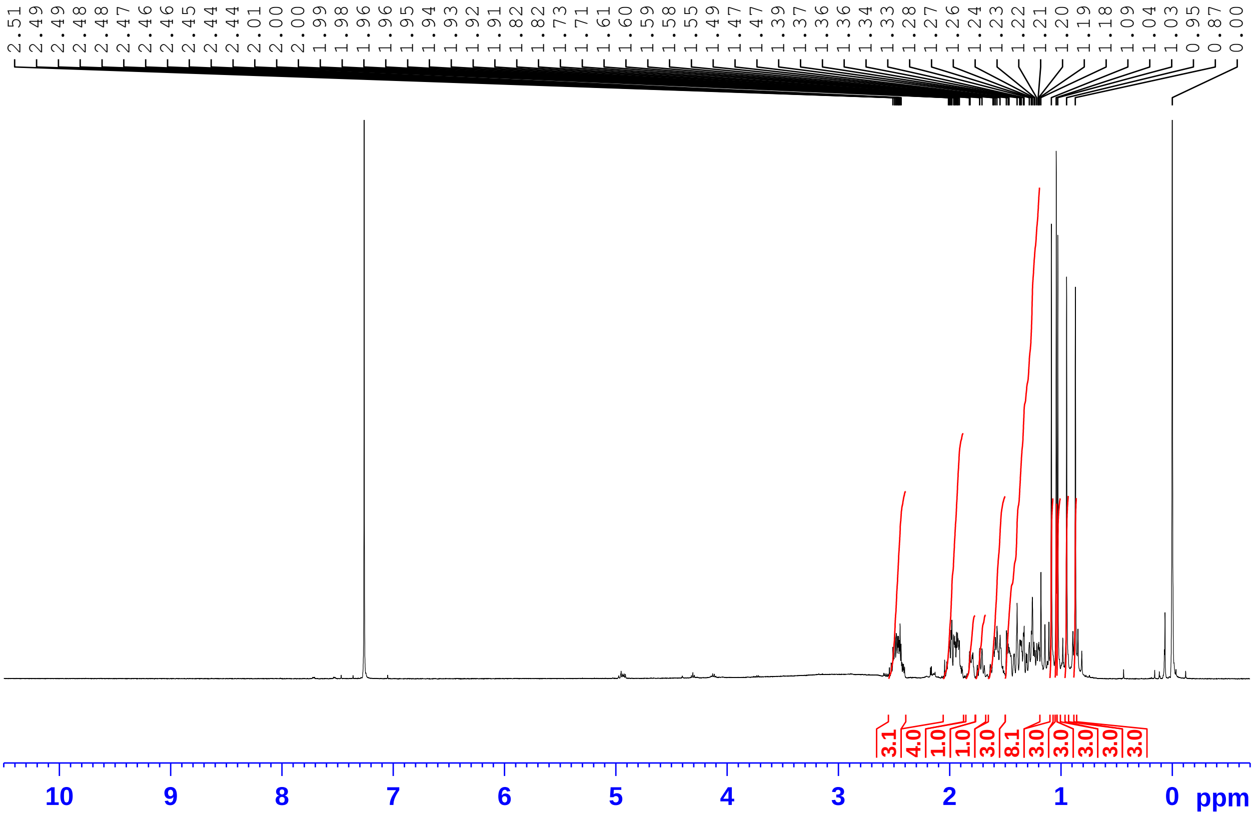

Figure S64. Complete $^{13}C\{^{1}H\}$ spectrum of compound **10** in $CDCl_3$, 125 MHz.

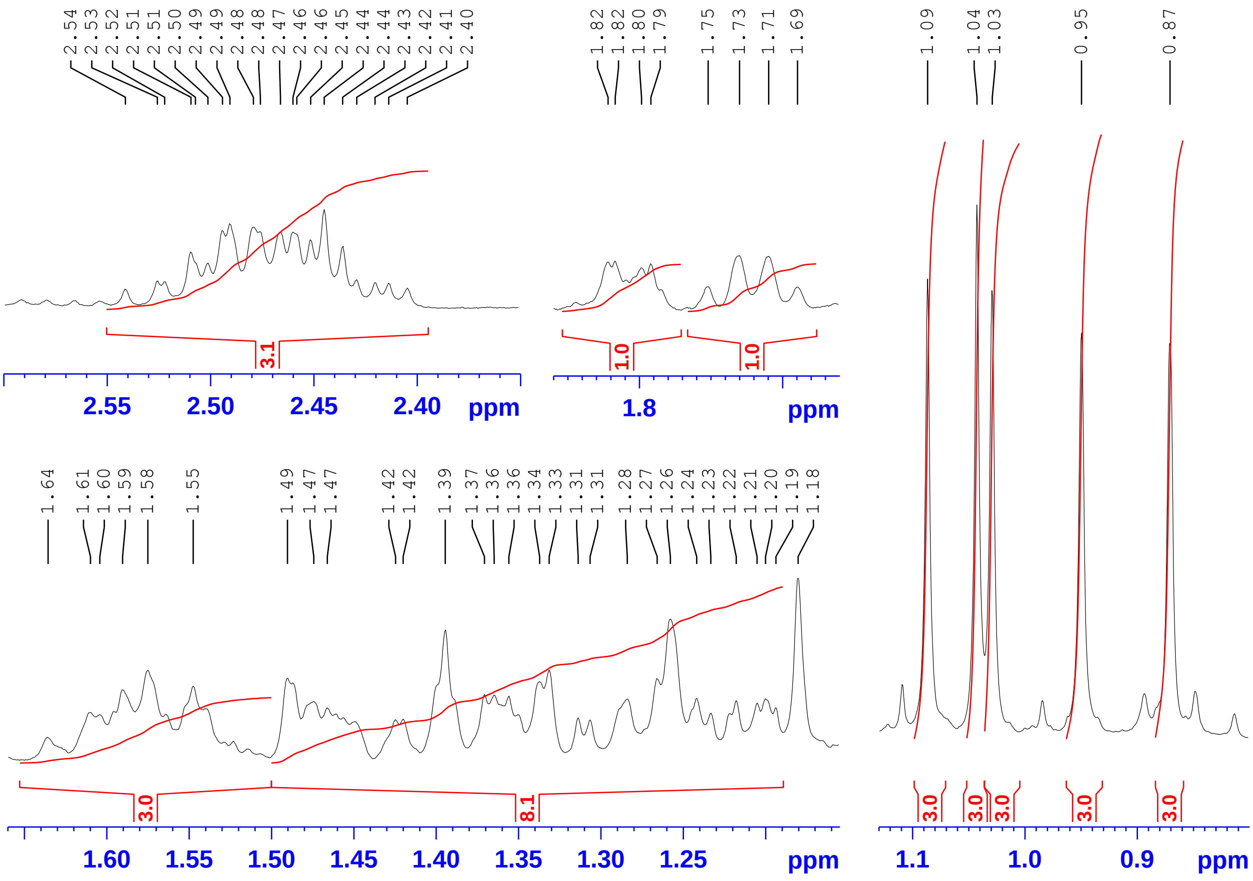

Figure S65. Expanded ^{1}H NMR spectrum of compound **10** in $CDCl_3$, 500MHz.

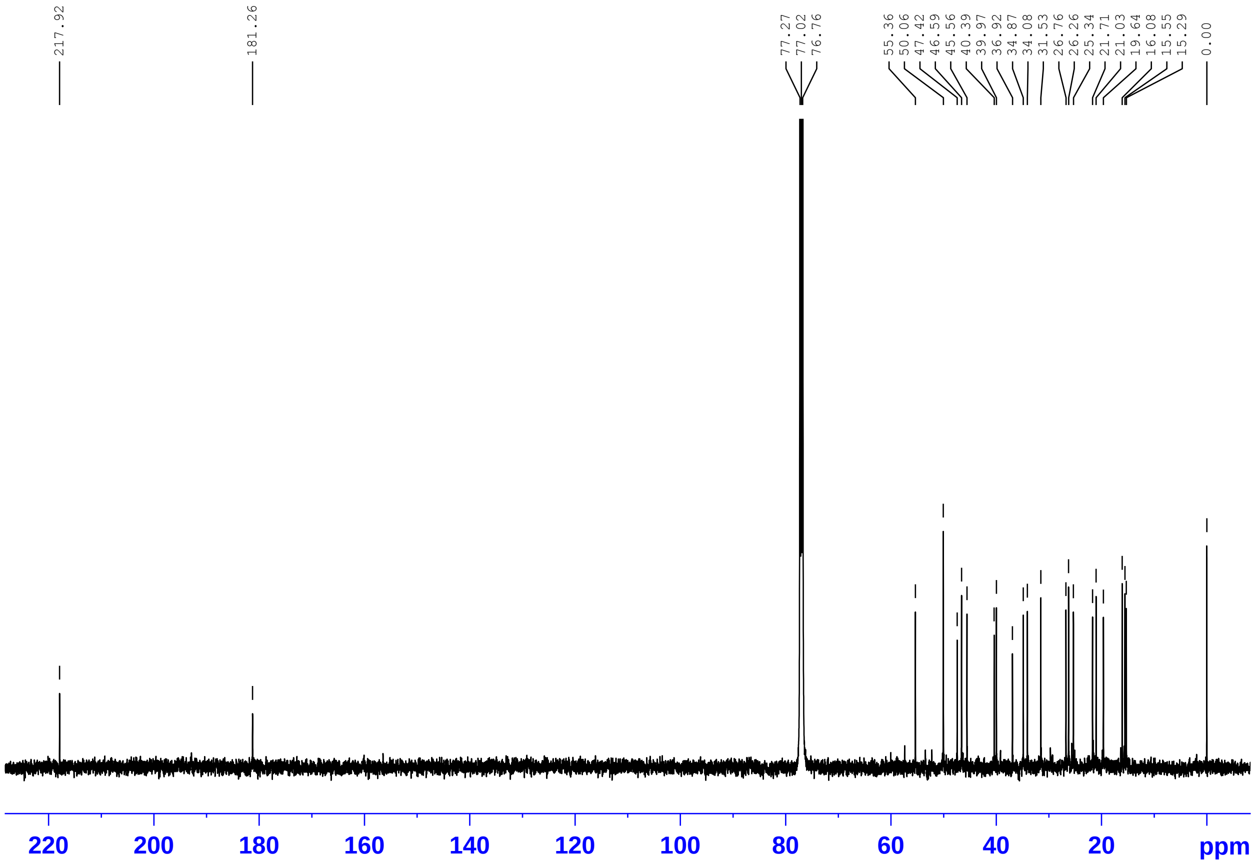

Figure S66. Complete $^{13}C\{^1H\}$ spectrum of compound **10** in $CDCl_3$, 125 MHz.

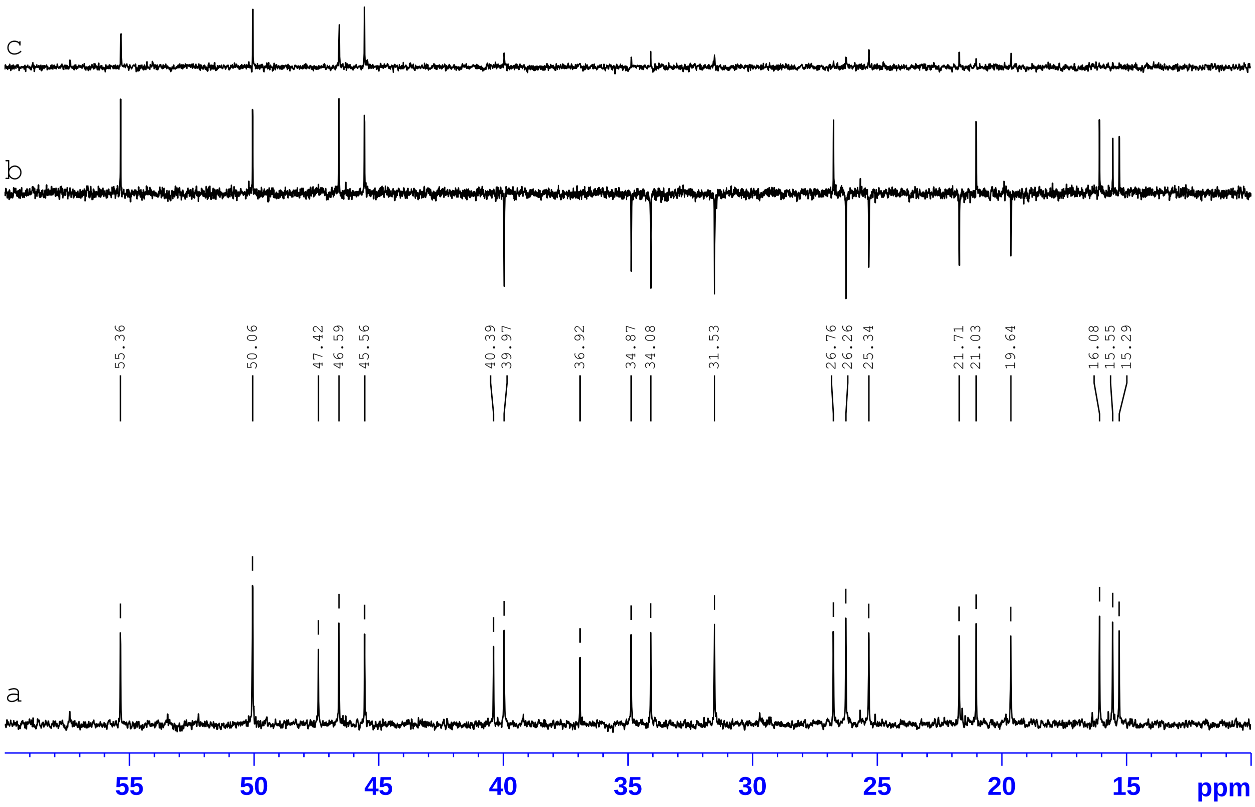

Figure S67. $^{13}C\{^1H\}$ (a), DEPT-135 (b) and DEPT-90 (c) spectra of compound **10** in $CDCl_3$, 125 MHz.

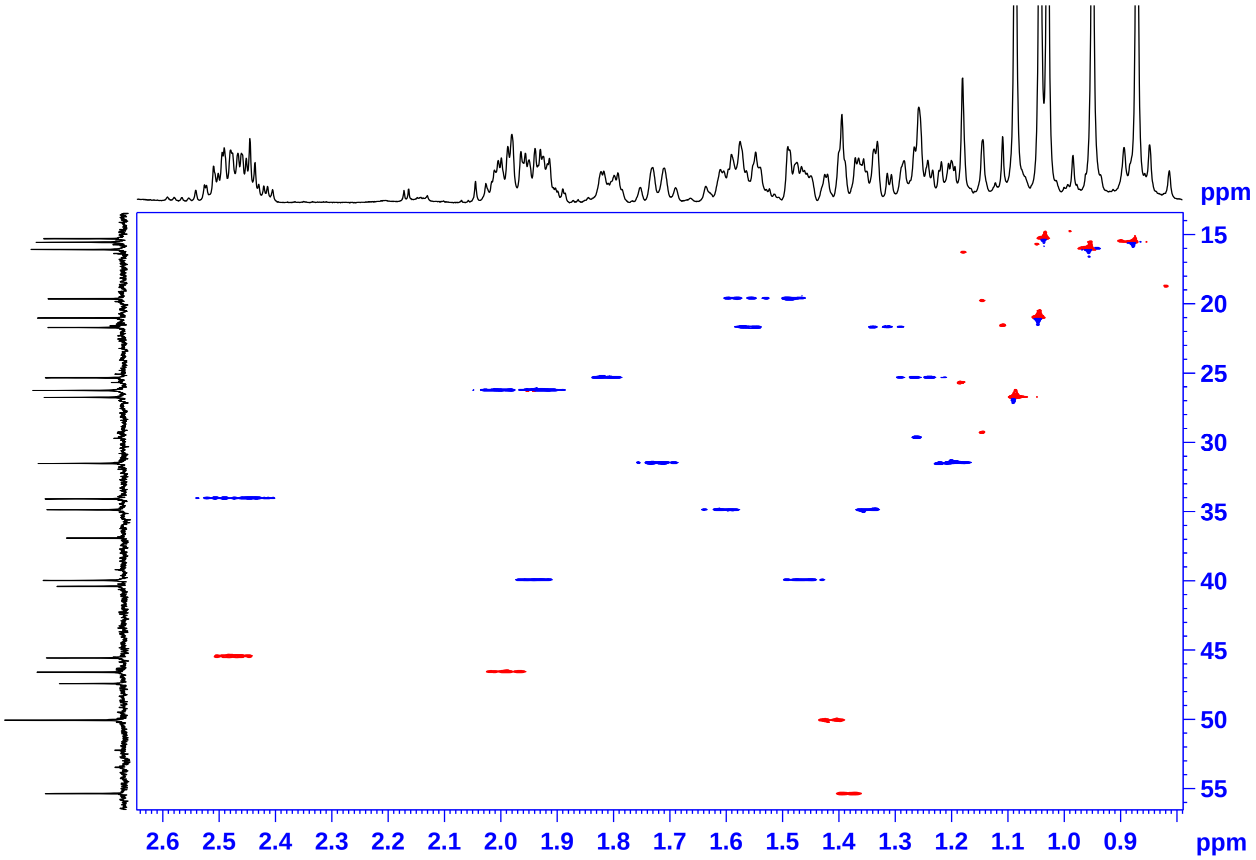

Figure S68. {^{1}H, ^{13}C} HSQC-edited spectrum of compound **10** in $CDCl_3$, 500 MHz.

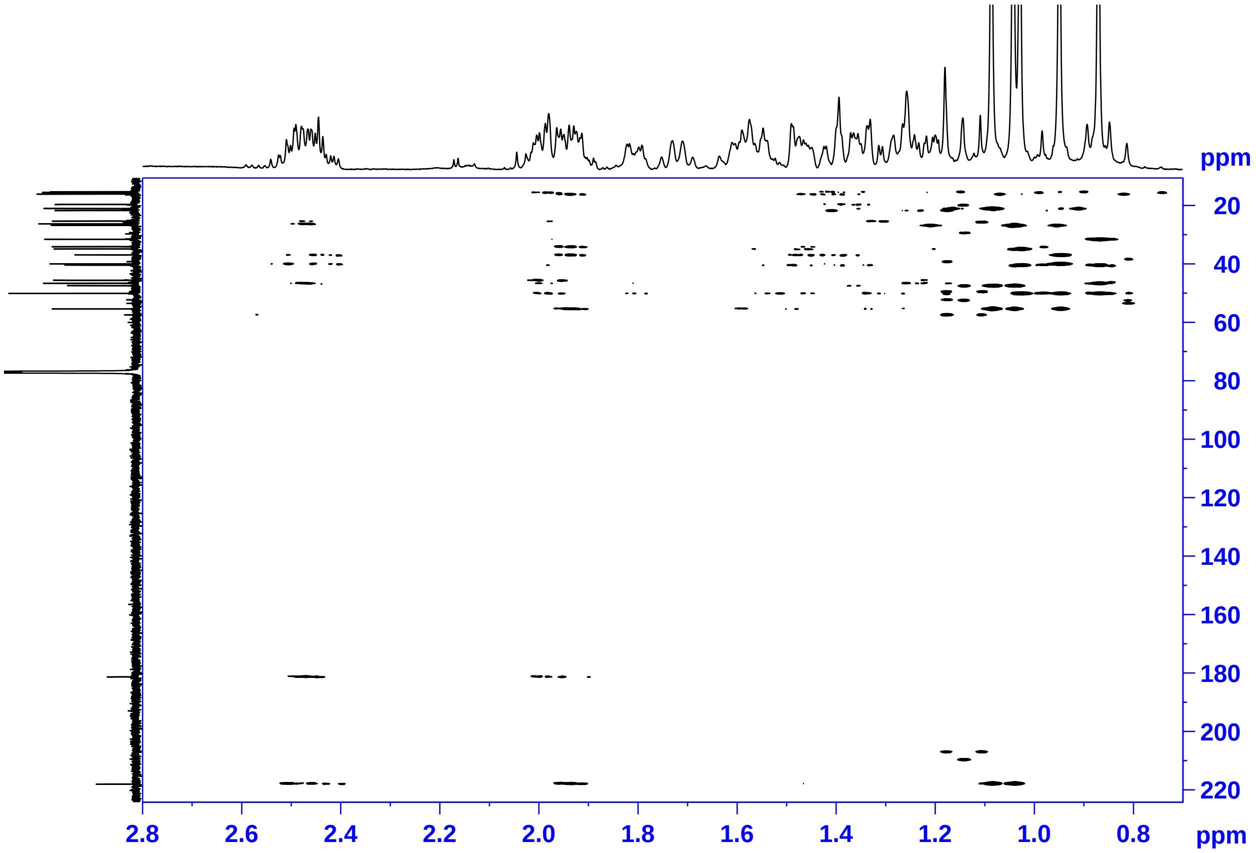

Figure S69. {^{1}H, ^{13}C} HMBC spectrum of compound **10** in $CDCl_3$, 500 MHz.

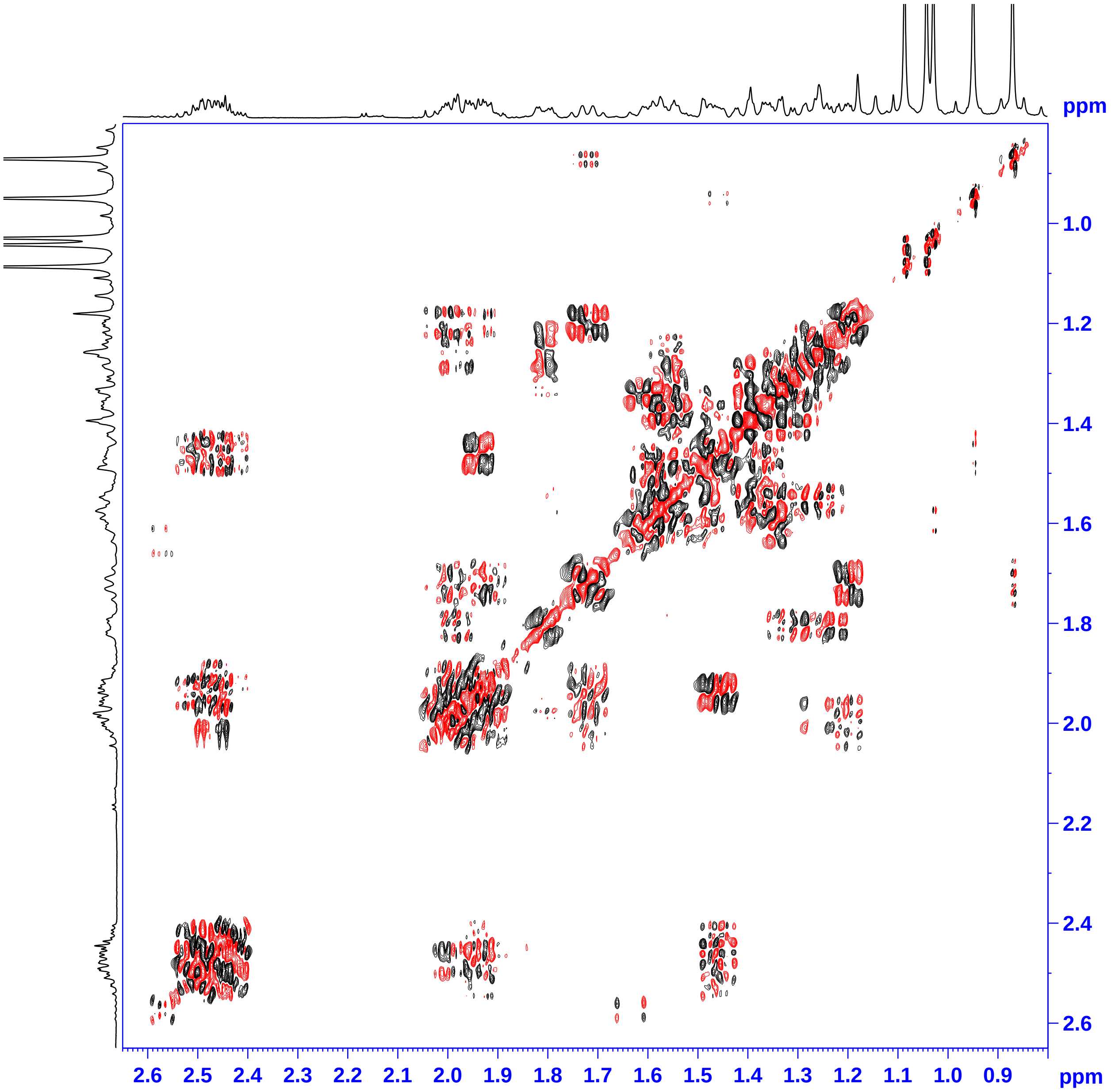

Figure S70. {^{1}H, ^{1}H} COSY spectrum of compound **10** in $CDCl_3$, 500 MHz.

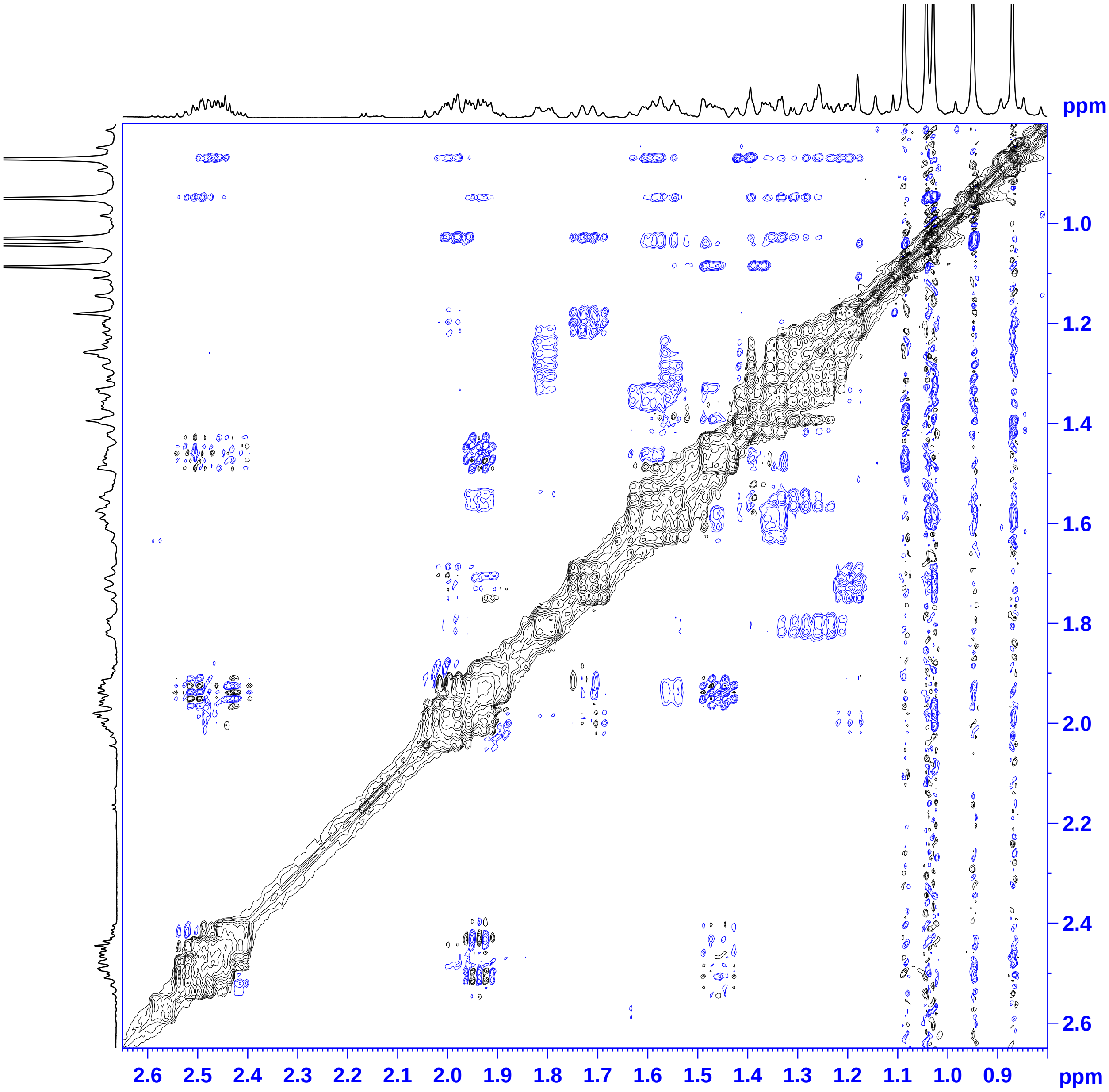

Figure S71. {1H, 1H} NOESY spectrum of compound **10** in $CDCl_3$, 500 MHz.

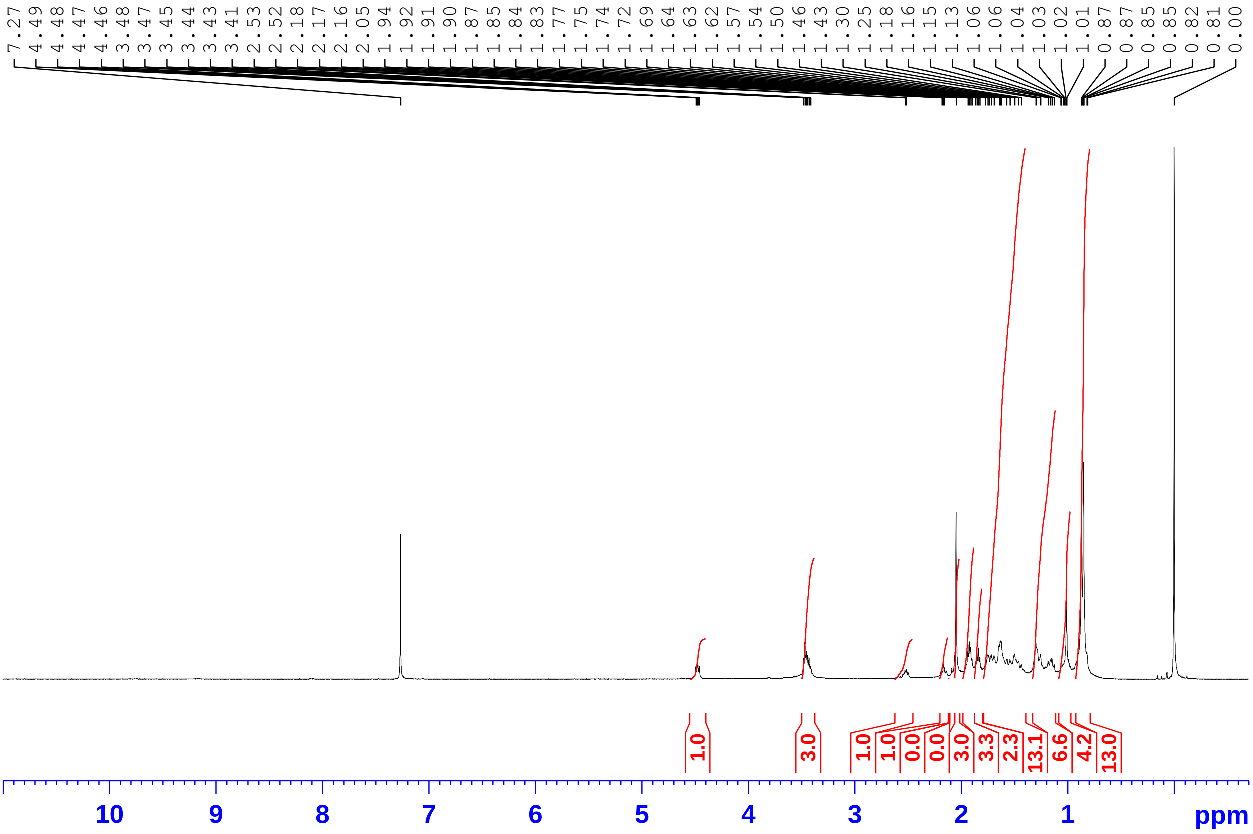

Figure S72. Complete $^{13}C\{^{1}H\}$ spectrum of compound **12** in $CDCl_3$, 125 MHz.

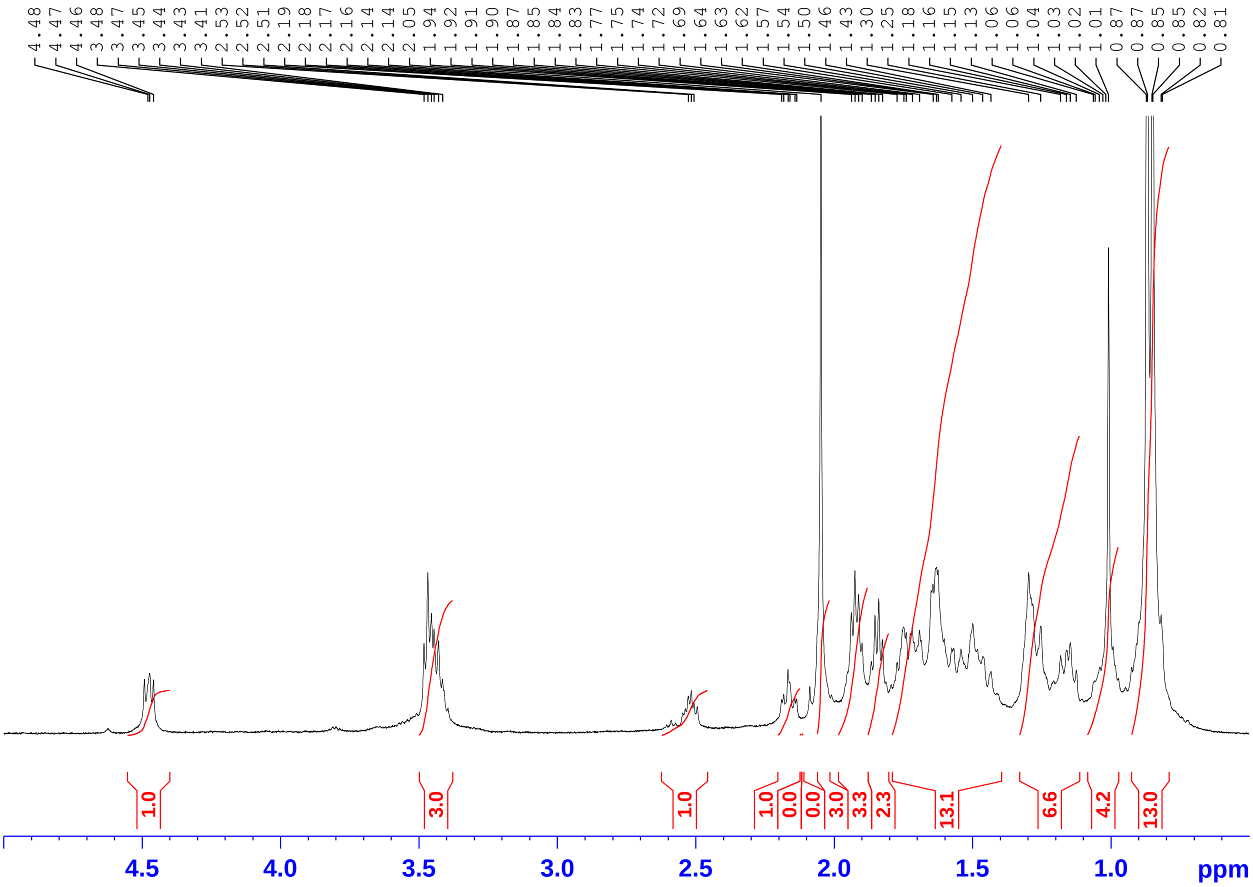

Figure S73. Expanded ^{1}H NMR spectrum of compound **12** in $CDCl_3$, 500MHz.

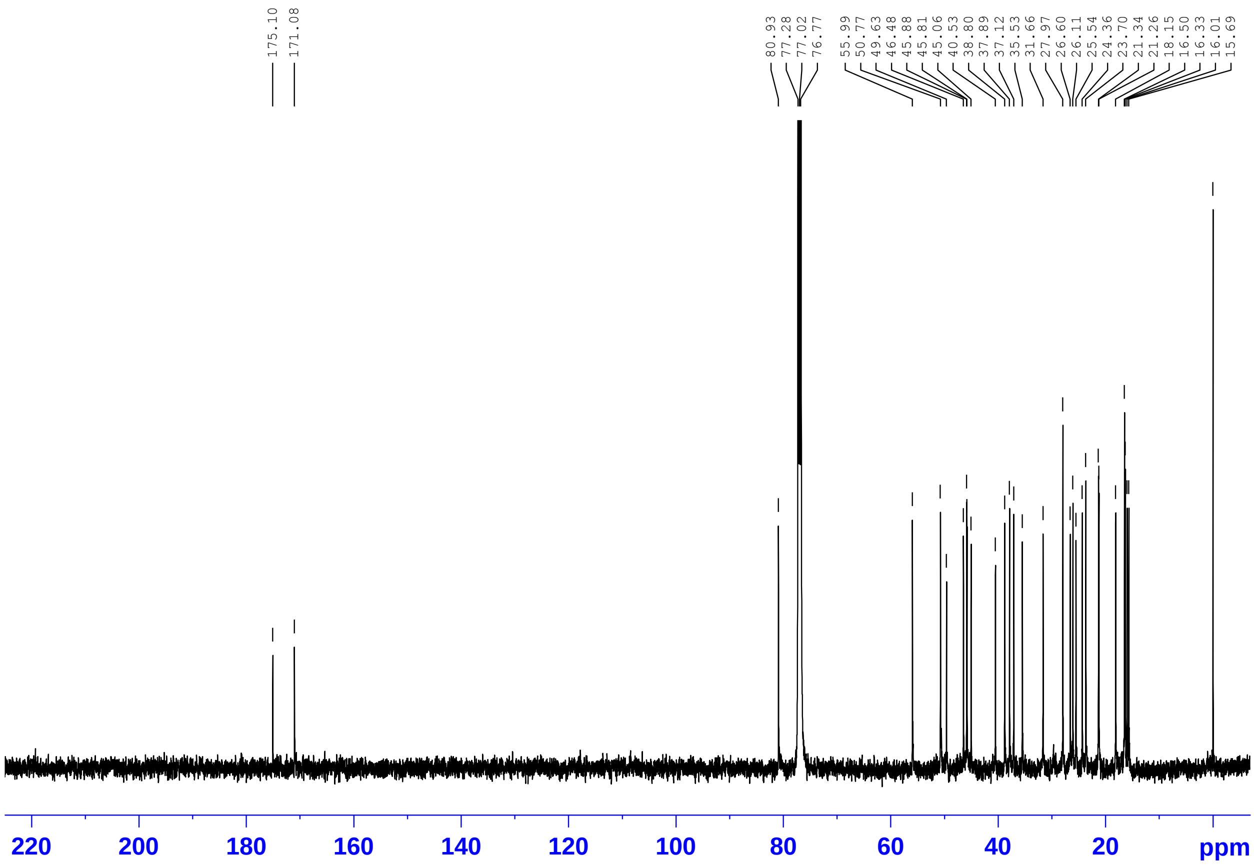

Figure S74. Complete $^{13}C\{^1H\}$ spectrum of compound **12** in $CDCl_3$, 125 MHz.

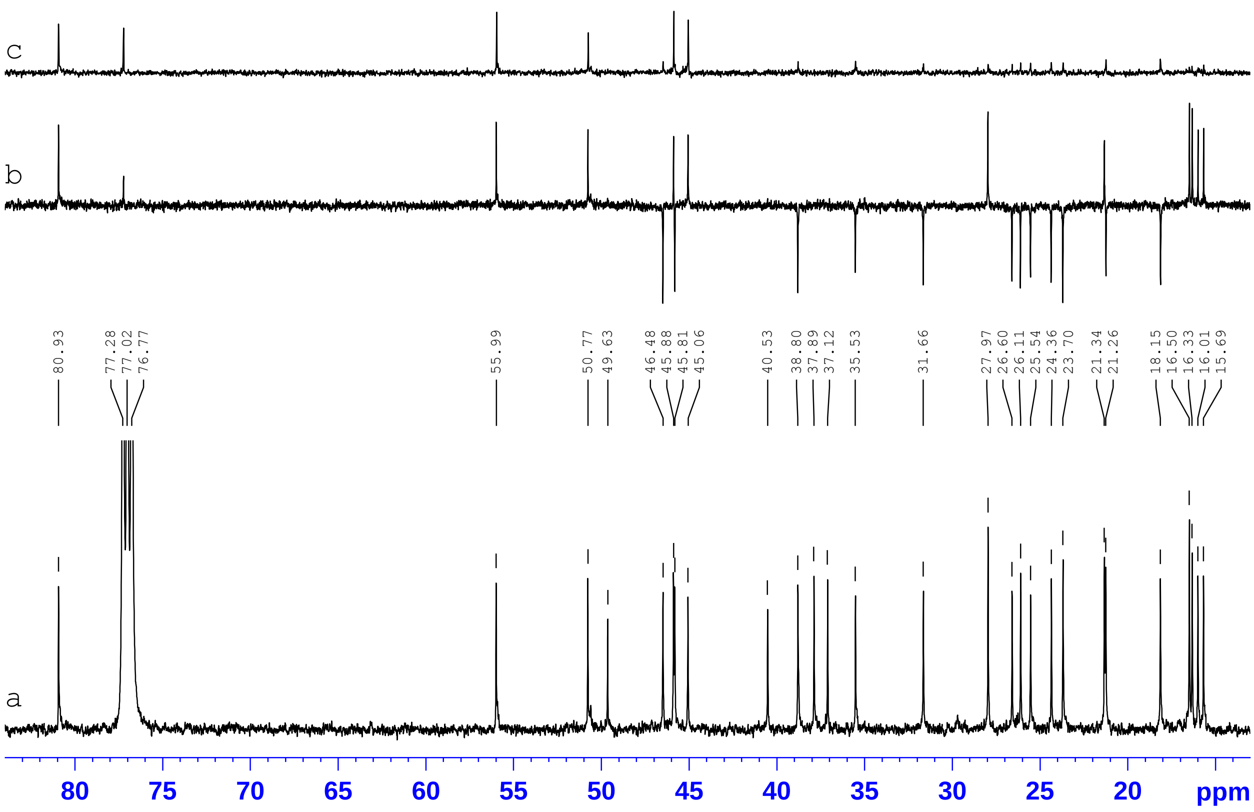

Figure S75. $^{13}C\{^1H\}$ (a), DEPT-135 (b) and DEPT-90 (c) spectra of compound **12** in $CDCl_3$, 125 MHz.

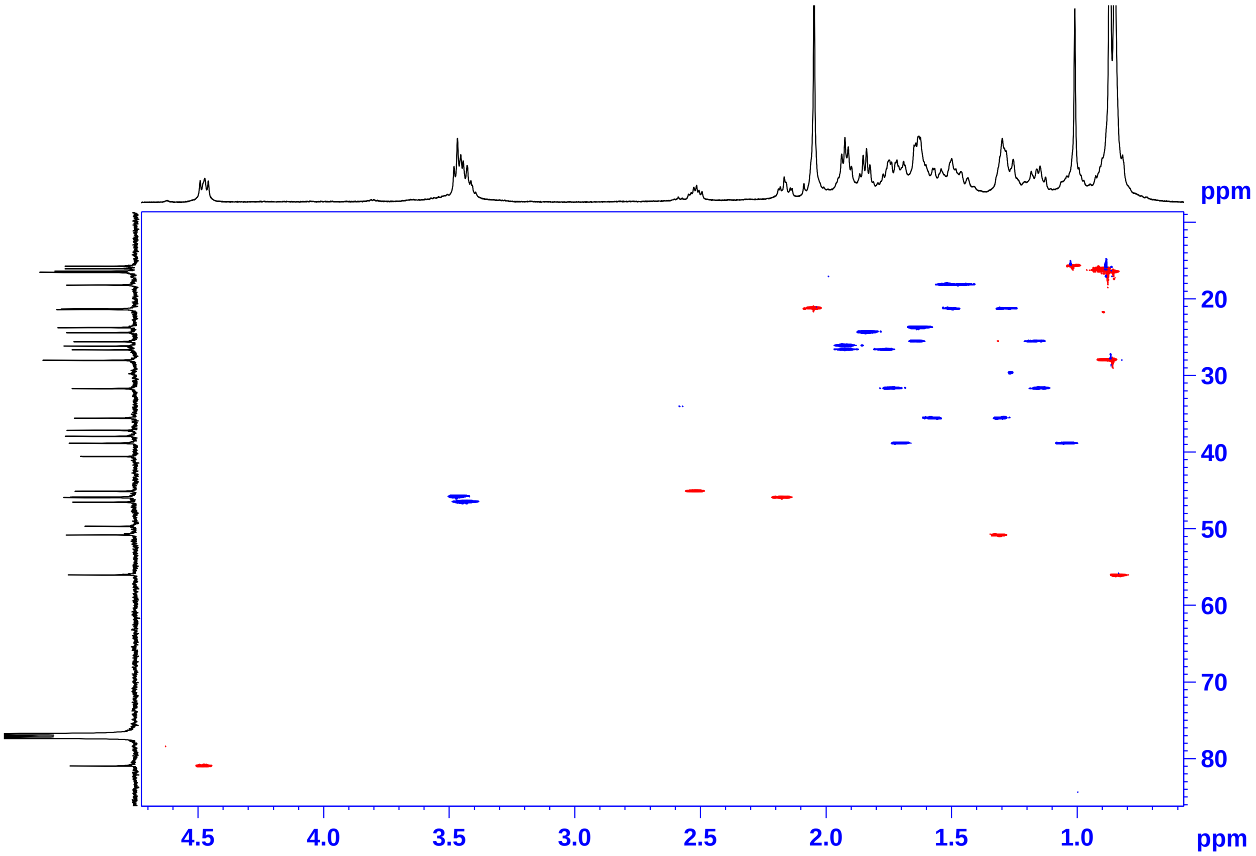

Figure S76. {^{1}H, ^{13}C} HSQC-edited spectrum of compound **12** in $CDCl_3$, 500 MHz.

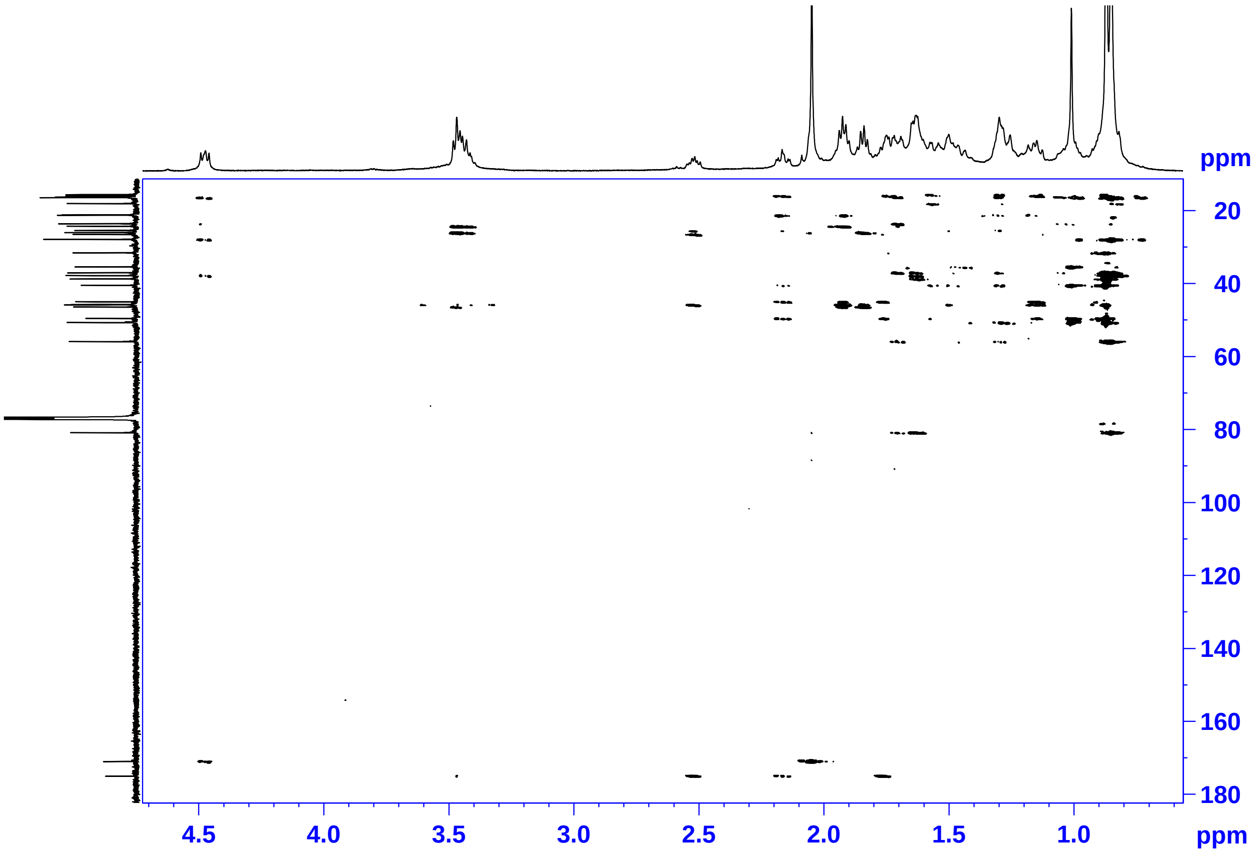

Figure S77. {^{1}H, ^{13}C} HMBC spectrum of compound **12** in $CDCl_3$, 500 MHz.

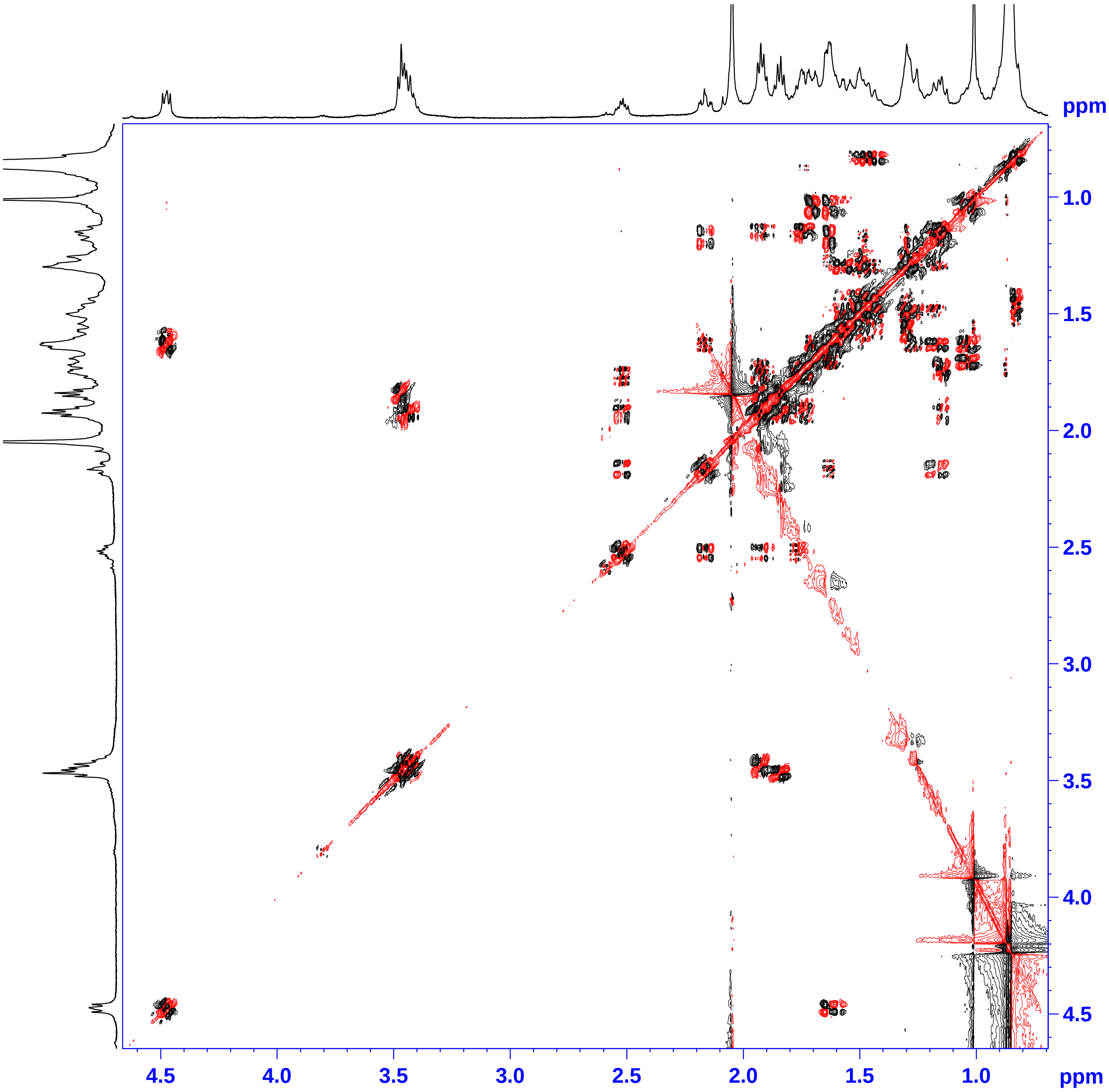

Figure S78. {^{1}H, ^{1}H} COSY spectrum of compound **12** in $CDCl_3$, 500 MHz.

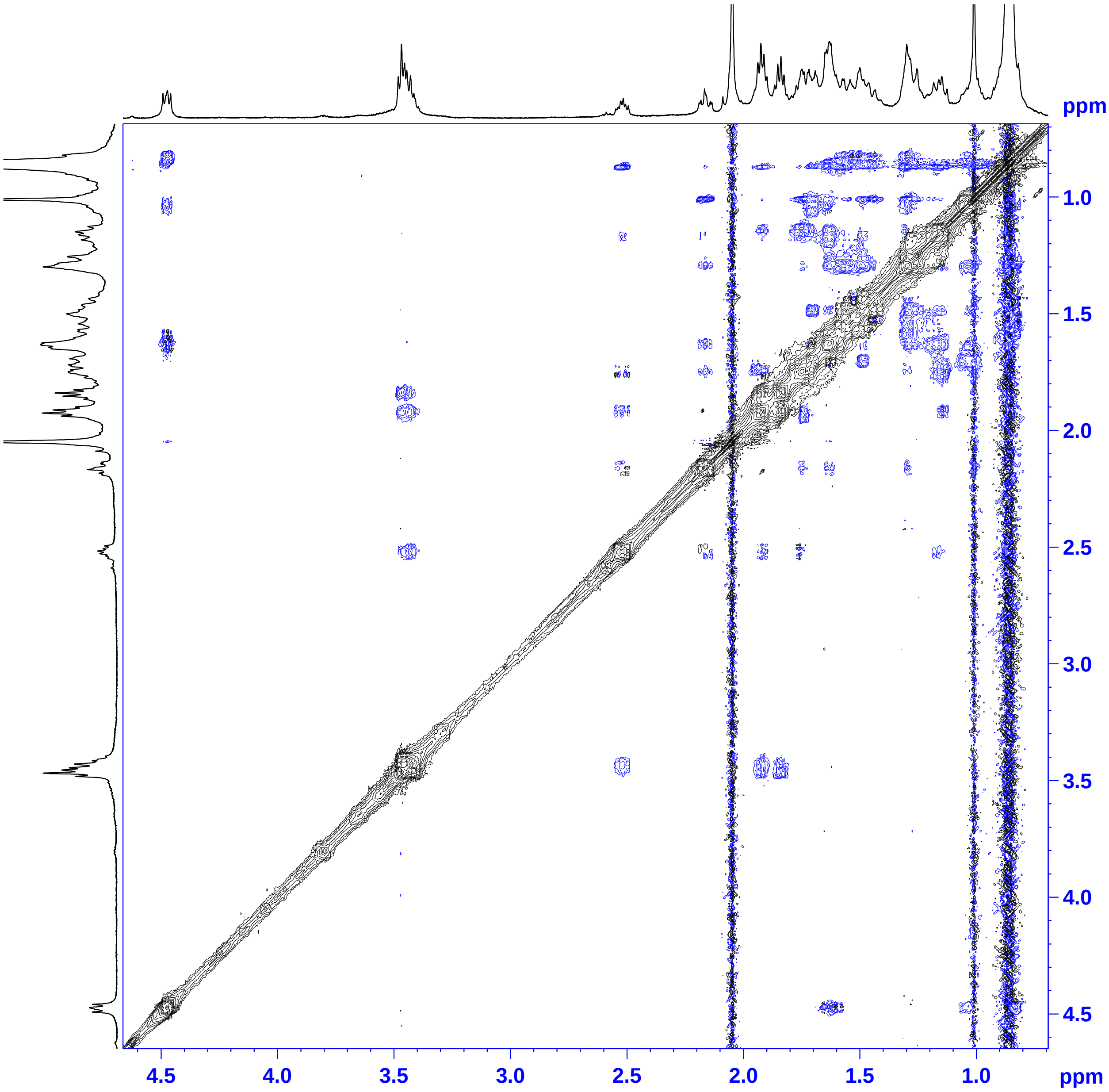

Figure S79. {^{1}H, ^{1}H} NOESY spectrum of compound **12** in $CDCl_3$, 500 MHz.
